# Peripheral amyloid-β clearance mediates cognitive impairment in non-alcoholic fatty liver disease

**DOI:** 10.1016/j.ebiom.2024.105079

**Published:** 2024-03-19

**Authors:** Xiaobo Peng, Xing Zhang, Zihui Xu, Linyan Li, Xiaoxing Mo, Zhao Peng, Zhilei Shan, Hong Yan, Jian Xu, Liegang Liu

**Affiliations:** a Department of Nutrition and Food Hygiene, Hubei Key Laboratory of Food Nutrition and Safety, School of Public Health, Tongji Medical College, Huazhong University of Science & Technology, Wuhan 430030, China; b Ministry of Education Key Lab of Environment and Health, School of Public Health, Tongji Medical College, Huazhong University of Science & Technology, Wuhan 430030, China; c Department of Elderly Health Management, Shenzhen Center for Chronic Disease Control, Shenzhen 518000, China

**Keywords:** NAFLD, Peripheral Aβ clearance, LRP-1, rs1799986 polymorphism, Aβ accumulation, Cognitive impairment

## Abstract

**Background:**

Non-alcoholic fatty liver disease (NAFLD) is a prevalent risk factor for cognitive impairment. Cerebral amyloid-β (Aβ) accumulation, as an important pathology of cognitive impairment, can be caused by impaired Aβ clearance in the periphery. The liver is the primary organ for peripheral Aβ clearance, but the role of peripheral Aβ clearance in NAFLD-induced cognitive impairment remains unclear.

**Methods:**

We examined correlations between NAFLD severity, Aβ accumulation, and cognitive performance in female Sprague–Dawley rats. The impact of NAFLD on hepatic Aβ clearance and the involvement of low-density lipoprotein receptor-related protein 1 (LRP-1) were assessed in rat livers and cultured hepatocytes. Additionally, a case–control study, including 549 NAFLD cases and 549 controls (782 males, 316 females), investigated the interaction between NAFLD and *LRP-1* rs1799986 polymorphism on plasma Aβ levels.

**Findings:**

The severity of hepatic steatosis and dysfunction closely correlated with plasma and cerebral Aβ accumulations and cognitive deficits in rats. The rats with NAFLD manifested diminished levels of LRP-1 and Aβ in liver tissue, with these reductions inversely proportional to plasma and cerebral Aβ concentrations and cognitive performance. In vitro, exposure of HepG2 cells to palmitic acid inhibited LRP-1 expression and Aβ uptake, which was subsequently reversed by a peroxisome proliferator-activated receptor α (PPARα) agonist. The case–control study revealed NAFLD to be associated with an increment of 8.24% and 10.51% in plasma Aβ40 and Aβ42 levels, respectively (both *P* < 0.0001). Moreover, the positive associations between NAFLD and plasma Aβ40 and Aβ42 levels were modified by the LRP-1 rs1799986 polymorphism (*P* for interaction = 0.0017 and 0.0015, respectively).

**Interpretation:**

LRP-1 mediates the adverse effect of NAFLD on peripheral Aβ clearance, thereby contributing to cerebral Aβ accumulation and cognitive impairment in NAFLD.

**Funding:**

Major International (Regional) Joint Research Project, 10.13039/501100012166National Key Research and Development Program of China, 10.13039/501100001809National Natural Science Foundation of China, and the Angel Nutrition Research Fund.

**Research in context**

**Evidence before this study**

Non-alcoholic fatty liver disease (NAFLD) is a prevalent risk factor for cognitive impairment, but the precise underlying mechanisms remain elusive. Recently, an increasing body of literature highlights the pivotal role of peripheral amyloid-β (Aβ) clearance in the development of cerebral Aβ accumulation and cognitive dysfunction. The liver is the primary organ for peripheral Aβ clearance, and clinical evidence has shown elevated circulating Aβ levels in patients with liver diseases such as hepatocellular carcinoma and cirrhosis. Previous animal study has also found increased Aβ accumulation in the brain of mice with NAFLD. These studies prompted us to hypothesize that NAFLD may exacerbate cerebral Aβ accumulation by attenuating peripheral Aβ clearance, further leading to cognitive impairment. In addition, low-density lipoprotein receptor-related protein 1 (LRP-1) plays a vital role in Aβ clearance and is highly expressed in hepatocytes. Moreover, a reduction of LRP-1 expression has been reported in the liver of rodents with NAFLD. Whether LRP-1 mediates the effect of NAFLD on peripheral Aβ clearance requires further investigation.

**Added value of this study**

By establishing a long-term NAFLD rat model, we elucidated positive correlations between the severity of hepatic steatosis and dysfunction, plasma and cerebral Aβ accumulations, and cognitive deficits. In the in vivo and in vitro studies, we further demonstrated the adverse effect of NAFLD on hepatic Aβ clearance through inhibiting LRP-1-mediated Aβ uptake. In the population-based study, we revealed NAFLD to be associated with an increment of 8.24% and 10.51% in plasma Aβ40 and Aβ42 levels, respectively. Moreover, the positive associations between NAFLD and plasma Aβ40 and Aβ42 levels were modified by the *LRP-1* rs1799986 polymorphism. Our findings suggest that LRP-1 mediates the adverse effect of NAFLD on peripheral Aβ clearance, thereby contributing to cerebral Aβ accumulation and cognitive impairment in NAFLD.

**Implications of all the available evidence**

Our study highlights the importance of peripheral Aβ clearance in NAFLD-induced cognitive impairment. Enhancing peripheral Aβ clearance may be a potential therapy for NAFLD-induced cognitive dysfunction and hepatic LRP-1 may be a key target for intervention.

## Introduction

Non-alcoholic fatty liver disease (NAFLD) has emerged as a prevailing chronic liver condition worldwide, affecting an estimated 25% of the general population.^1^ Given its multisystem nature, NAFLD is associated with various extrahepatic complications, including type 2 diabetes, cardiovascular disease (CVD), and chronic kidney disease.^2^^,^^3^ Moreover, mounting evidence suggests a connection between NAFLD and brain health. Individuals with NAFLD exhibit an increased susceptibility to cognitive impairment, Alzheimer’s disease (AD), and dementia, even when concurrent metabolic comorbidities are taken into account.^4^, ^5^, ^6^, ^7^ Nevertheless, the precise underlying mechanisms remain elusive.

Amyloid-β (Aβ), consisting mainly of Aβ40 and Aβ42, represents a protein byproduct primarily produced within the central nervous system (CNS). Excessive accumulation of Aβ in the brain, resulting from an imbalance between its production and clearance, has been implicated in various cognitive disorders.^8^, ^9^, ^10^ Intriguingly, about 40–60% of brain-derived Aβ is conveyed into the bloodstream and eliminated through the peripheral system,^11^, ^12^, ^13^ underscoring the crucial role of the peripheral system in clearing Aβ from the CNS. The liver, serving as the principal organ for Aβ clearance in the periphery, accounts for over 60% of peripheral Aβ clearance.^14^ Elevated circulating Aβ levels have been observed in patients with liver diseases such as hepatocellular carcinoma and cirrhosis.^15^^,^^16^ A recent epidemiologic study has also demonstrated a significant association between liver function and cerebral Aβ accumulation.^17^ Furthermore, mice with NAFLD exhibit increased Aβ accumulation in the brain.^18^ These intriguing findings prompted us to hypothesize that NAFLD may exacerbate cerebral Aβ accumulation by attenuating peripheral Aβ clearance, further leading to cognitive impairment.

Low-density lipoprotein receptor-related protein 1 (LRP-1) plays a vital role in Aβ clearance.^19^ Previous genetic studies have linked a specific *LRP-1* allele (rs1799986) to cerebral Aβ accumulation and AD in humans.^20^ Importantly, LRP-1 is highly expressed in hepatocytes, facilitating hepatic uptake of circulating Aβ.^21^ Hepatic LPR-1 expression tends to decline with age, leading to impaired peripheral Aβ clearance in aged rats.^21^ The high-fat diet (HFD), a major inducer of NAFLD, has been reported to inhibit LRP-1 expression in the liver.^22^^,^^23^ However, whether LRP-1 mediates the effects of NAFLD on peripheral Aβ clearance requires further investigation.

Therefore, we examined the impact of NAFLD on Aβ accumulation and cognitive impairment in rodent models, investigating the underlying mechanisms both in vivo and in vitro, with a specific focus on LPR-1 mediated peripheral Aβ clearance. Furthermore, we explored the interaction between NAFLD and *LRP-1* rs1799986 on plasma Aβ levels in a case–control study.

## Methods

### Animals

Twenty-four female specific pathogen-free Sprague–Dawley rats (200–280 g, 3-month-old) were obtained from the Vital River Laboratory Animal Center (Beijing, China). Female rats were chosen because of their higher propensity for cognitive impairment with ageing than male rats.^24^ After a 1-month acclimatization period, the rats were randomly assigned to two groups: control and HFD (*n* = 12 per group) with the use of the RAND function in Microsoft Excel software. They were fed either a chow diet (10 kcal% fat; AIN-93M, Research Diets, New Brunswick, NJ, USA) or HFD (45 kcal% fat; D12451, Research Diets) for 12 months. The rats were housed in a specific pathogen-free environment with controlled temperature (22 ± 2 °C) and a 12-h light–dark cycle. They had unrestricted access to food and water throughout the study. Changes in the body weight, food intake, and energy intake of the rats are shown in Supplementary Fig. S1. One rat from the HFD group succumbed to a pituitary tumour after a 10-month feeding period, resulting in a total of 23 rats being included in subsequent experiments. Animals received human care and the experimental procedure received approval from the Experimental Animal Ethics Committee of Tongji Medical College, Huazhong University of Science & Technology (Permission ID: 2518).

### Behavioural tests

After the 12-month feeding period, all rats underwent a series of behavioural tests, including the open field test, new object recognition (NOR) test, elevated plus-maze test, and Morris water maze (MWM) test. These assessments were conducted in a dimly lit room with rats acclimated to the testing conditions for 1 h before each test. The apparatuses, video tracking systems, and analysis software for behavioural tests were acquired from AniLab Scientific Instruments (Ningbo, China).

Spontaneous locomotor activity was evaluated by the open field test, using an apparatus divided into central and marginal areas. The rats were placed in the central area and allowed 5 min of free exploration. Parameters such as the time spent in the central area (s), ambulatory time (s), resting time (s), moving distance (cm), and moving speed (cm/s) were quantified.

The NOR test was conducted in a circular area to assess short-term working memory, comprising three phases with a 24-h interval: habituation, familiarization, and test phases. The rats were given 5 min to explore a circular area during the habituation phase. In the subsequent familiarization phase, the rats explored the arena containing two identical objects for 5 min. Moving to the test phase, the rats were placed in the arena with one of the original objects and a new object, freely exploring for 5 min. The time spent on both the original and new objects (s) was recorded. The recognition index and discrimination index were calculated as follows: recognition index = time spent on the new object ÷ total exploration time; discrimination index = (time spent on the new object − time spent on the original object) ÷ total exploration time.

The elevated plus-maze test was used to assess spontaneous anxiety-like behaviour and was conducted using an apparatus comprising two open arms and two closed arms. The rats were placed in the central area and allowed 5 min of free exploration. The number of entries into the open and closed arms and the time spent in both types of arms were recorded.

The MWM test is a widely utilized method to evaluate spatial learning and memory in rodent models. The MWM apparatus was a circular pool filled with non-toxic carbon-opacified water heated to around 25 °C. This circular pool was divided into four quadrants, with a hidden platform in the first quadrant. The test comprised a 5-day training phase followed by a 1-day probe trial with a 24-h interval. During training, the rats were placed in the water in the quadrant without the platform and trained to locate the hidden platform three times daily. Rats that failed to find the platform within 60 s were guided to it and kept there for 15 s. The time taken for each rat to locate the hidden platform was recorded. During the probe trial, rats were placed in the water in the fourth quadrant and allowed to swim freely for 60 s. Time (s) and distance (cm) spent in the target quadrant, number of platform crossings, and swimming speed (cm/s) were recorded.

### Histological examination

Seven rats (*n* = 4 for the control group; *n* = 3 for the HDF group) were anaesthetized and perfused with 4% ice-cold phosphate-buffered paraformaldehyde. Subsequently, both the brain and liver tissues were harvested and fixed in 4% phosphate-buffered paraformaldehyde for 24 h. For hematoxylin-eosin (H&E) staining, the fixed liver tissues were embedded in paraffin and sectioned. The sections underwent dewaxing, rehydration, and staining with hematoxylin for nuclei and eosin for cytoplasm visualization. In the case of Oil red O staining, the fixed liver tissues were embedded in O.C.T gel and sectioned. The sections were then stained with Oil Red O to visualize the lipid droplets, along with hematoxylin for nuclear staining. For immunohistochemistry, the fixed brain tissues were embedded in paraffin and sectioned. Following dewaxing, rehydration, and antigen retrieval, the sections were incubated with an anti-Aβ antibody (6E10, BioLegend Cat# 803001, RRID: AB_2564653) and biotinylated secondary antibody (Thermo Fisher Scientific Cat# 31800, RRID: AB_228305). Immuno-reactions were visualized using the SABC-HRP kit from Beyotime (Cat# P0612, Shanghai, China), and nuclei were counterstained with hematoxylin. All images were captured by Olympus Ⅸ-71 (Tokyo, Japan).

### Kit assays for animals

The levels of Aβ (Elabscience Cat# E-EL-R3030 and E-EL-R1402c, Wuhan, China) and LRP-1 (ELK Cat# ELK8974, Wuhan, China) in rat tissues were determined using ELISA kits with 30 mg of tissue. Hepatic TG, plasma ALT, and AST levels were detected using a colorimetric assay (Elabscience Cat# E-BC-K261-M, E-BC-K235-M, and E-BC-K236-M, respectively) with 30 mg of liver tissue or 5 μL of plasma. Plasma Aβ levels were measured in 25 μL of plasma using an ECLIA assay (Meso Scale Discovery Cat# K15199E, Rockville, MD, USA). All assays were performed with 8 rats per group following the manufacturer’s instructions, and the investigators were blinded to the grouping.

### Cell culture and treatment

The human hepatoma cell line HepG2 was acquired from the American Type Culture Collection (Rockville, MD, USA), and all cell culture reagents were sourced from Gibco (Grand Island, NY, USA). The cell line was validated by STR profiling and tested negative for mycoplasma. HepG2 cells were cultured in minimal essential medium (Cat# 41090036) supplemented with 10% (v/v) fetal bovine serum (Cat# 10091155), 1% (v/v) non-essential amino acids (Cat# 11140050), 1 mM sodium pyruvate (Cat# 11360070), and 1% (v/v) penicillin-streptomycin (Cat# 10378016). Cells were maintained in a humidified atmosphere containing 5% CO_2_ at 37 °C. To induce noticeable steatosis without significantly affecting the cell viability (Supplementary Fig. S2), HepG2 cells were treated with 400 μM palmitic acid (PA; Sigma Cat# P0500, St Louis, MO, USA) for 24 h. In separate experiments, HepG2 cells were co-incubated with 400 μM PA and peroxisome proliferator-activated receptor α (PPARα) agonist (WY14643; MedChemExpress Cat# HY-16995, Monmouth Junction, NJ, USA) or PPARγ inhibitor (T0070907; MedChemExpress Cat# HY-13202) for 24 h.

### Cell viability assay

HepG2 cells in a 96-well plate were treated with varying concentrations of PA (0, 200, 400, 600, 800, or 1000 μM) or co-treated with 400 μM PA and different doses of WY14643 (0, 5, 10, 25, or 50 μM) or T0070907 (0, 1, 5, 10, or 15 μM) for 24 h. Subsequently, cell viability was determined using a Cell Counting Kit-8 (Dojindo Cat# CK04, Kyushu Island, Japan).

### Aβ uptake assay

After treatment, HepG2 cells in a 12-well plate were washed three times with PBS and incubated in a complete medium containing 500 nM Cy5-labelled Aβ40 or Aβ42 (QYAOBIO, Shanghai, China) for 1 h. Following incubation, the cells were washed three times with ice-cold PBS and collected for fluorescence measurement using a FACS Caliber (BD Biosciences, Franklin Lakes, NJ, USA). The mean fluorescence intensity was quantified using the FlowJo software version 10.

### Western blotting

Rat tissues and HepG2 cells were homogenized in ice-cold RIPA lysis buffer (Beyotime Cat# P0013B) containing 1% (v/v) phenylmethanesulfonyl fluoride (Beyotime Cat# ST505). The total protein concentration was determined using a BCA assay kit (Beyotime Cat# P0010). Protein samples were then incubated with primary and secondary antibodies (Cell Signalling Technology Cat# 7074, RRID: AB_2099233) and protein bands were visualized using an ECL system (Syngene, UK). Protein bands were analyzed using Image J software, and the expression of target proteins was normalized to that of GAPDH. Detailed information regarding the primary antibodies used was as follows: APP (Proteintech Cat# 25524-1-AP, RRID: AB_2880118); IDE (Abcam Cat# ab32216, RRID: AB_775686); NEP (Abcam Cat# ab256494, RRID: AB_2894853); PPARα (Abcam Cat# ab227074, RRID: AB_3083737); PPARγ (Abcam Cat# ab209350, RRID: AB_2890099); LRP-1 (Cell Signalling Technology Cat# 64099S, RRID: AB_2799654), and GAPDH (Cell Signalling Technology Cat# 2118, RRID: AB_561053).

### Quantitative real-time (qRT)-PCR

Total RNA was extracted from rat tissues and HepG2 cells using a kit from Tiangen (Cat# DP419, Beijing, China) and applied to synthesize cDNA by Quantscript RT kit (Tiangen Cat# KR103). The synthesized cDNA was then amplified by qRT-PCR using the RealUniversal Colour PreMix (Tiangen Cat# FP201). The expression of target mRNA was normalized to that of GAPDH. The primers used in qRT-PCR were as follows: APP (forward, ATTGCCACCACTACCACAA; reverse, CACATCCGCCGTAAAAGA); IDE (forward, TTACCAGCGGAGAACACACC; reverse, GCATCAAACAAGGGGCACAG); NEP (forward, CTTTAGTGCTCGGCAGTCCA; reverse, CACCAGTCAACGAGGTCTCC); LRP-1 (forward, CAACACCACCTGCTACGAGT; reverse, GCTCTCGGGCATCATAGTCC); and GAPDH (forward, ACTCCCATTCTTCCACCTTTG; reverse, GGCCTCTCTCTTGCTCTCAGT).

### Study population

The case–control study was conducted within the Tongji-Ezhou cohort (NCT03845868) and comprised 549 NAFLD cases and 549 controls without NAFLD. A flowchart illustrating participant recruitment and case–control selection is provided in Supplementary Fig. S3. Participants were recruited from Echeng Steel and received healthcare at Echeng Steel Hospital (Ezhou, China) from April to July 2013. We included participants who met conditions as follows: age ≥ 30 years, body mass index (BMI) < 40 kg/m^2^, no history of certain chronic hepatitis (Wilson disease, hepatitis A, B, and C), autoimmune liver disease, drug-induced liver disease, cerebrovascular disease, or kidney disease, and no pharmacological treatment for diabetes or hyperlipidaemia. Individuals with excessive alcohol consumption (>140 g/week for men and >70 g/week for women) were also excluded.

Abdominal ultrasound scans were performed by experienced sonographers and fatty liver was diagnosed according to the criteria recommended by the Chinese Society of Hepatology in 2008.^25^ Cases of fatty liver were further classified as mild, moderate, or severe based on guidelines. Controls were randomly selected from participants without fatty liver disease and matched to cases 1:1 by age (±3 years) and sex. The study protocol was approved by the Ethics Committee of Tongji Medical College, Huazhong University of Science & Technology (Permission ID: A214), and conducted following the Declaration of Helsinki. All participants included in this study provided written informed consent.

### Data collection and laboratory measurements for humans

Sociodemographic characteristics including age, sex, lifestyle habits, medical history, and medication use were obtained through a standardized questionnaire. Anthropometric characteristics including height (m), weight (kg), and blood pressure (mmHg) were measured using standard methods. BMI was calculated by dividing weight by height squared. Definitions for diabetes, hyperlipidaemia, and hypertension were consistent with previous literature.^26^

Plasma levels of various biochemical parameters, including fasting plasma glucose, TG, total cholesterol, LDL cholesterol, HDL cholesterol, ALT, and creatinine, were determined using enzymatic reaction methods on a Hitachi 7180 automatic analyser. The glomerular filtration rate (GFR) was estimated using new equations based on creatinine levels.^27^

Simultaneously, plasma levels of Aβ40 and Aβ42 were quantified using an ECLIA assay (Meso Scale Discovery Cat# K15199E). The intra- and inter-assay coefficients of variation for Aβ40 and Aβ42 detection were <10%. All subjects had measurable plasma levels of Aβ40 and Aβ42 that were above the limit of detection.

The *LRP-1* rs1799986 polymorphism was assessed using the MassArray System (Agena, San Diego, CA, USA). Genomic DNA was isolated from blood samples using a kit from Tiangen (Cat# DP348). Subsequently, the DNA samples underwent multiplex PCR amplification, and the resulting products were subjected to a locus-specific single-base extension reaction. Alleles were identified using a MassARRAY Analyser (Agena). The genotyping success rate exceeded 99%, and the genotype distribution adhered to Hardy–Weinberg equilibrium.

### Statistical analysis

In vivo and in vitro data were presented as mean ± SD. All continuous data were tested for normality using the Shapiro–Wilk normality test. Normally distributed data were compared using unpaired Student’s *t*-test (equal variances) or Welch’s *t*-test (unequal variances) for two groups, one-way ANOVA with Dunnett’s multiple comparisons test for three or more groups, and two-way ANOVA for repeated measurements. Non-normally distributed data were compared using the Mann–Whitney *U* test for two groups and the Scheirer-Ray-Hare test for repeated measurements. Meanwhile, Spearman’s test and linear regression were used for correlation analyses.

For the population study, the normality assumption for quantitative data was assessed by Q–Q plots. The differences were assessed using unpaired Student’s *t*-test (equal variances) or Welch’s *t*-test (unequal variances) for normally distributed data, Mann–Whitney *U* test for non-normally distributed data, and χ^2^ test for categorical data. Linear regression was employed to evaluate the associations of NAFLD, plasma ALT levels, severity of NAFLD, history of diagnosed NAFLD, and rs1799986 polymorphism with plasma Aβ40 and Aβ42 levels. In linear regression analyses, plasma Aβ40 and Aβ42 levels were logarithmically transformed and multiplied by 100, interpreting the regression coefficients as symmetric percentage differences in the means of plasma Aβ.^28^^,^^29^ Multivariable models were adjusted for age, sex, BMI, current smoking status, current drinking status, physical activity, history of diabetes, history of hypertension, history of CVD, TG, total cholesterol, and eGFR, which were selected based on causal directed acyclic graph (Supplementary Fig. S4). Additionally, the associations between NAFLD and plasma Aβ40 and Aβ42 levels were stratified by rs1799986 polymorphisms (CC or CT + TT genotypes). The interaction between NAFLD and rs1799986 polymorphism was examined by determining the significance of multiplicative terms using the likelihood ratio test in multivariable linear regression models. Statistical power for the interaction tests was calculated using QUANTO 1.2.4 (http://hydra.usc.edu/gxe). According to the associations of NAFLD and rs1799986 genotype with plasma Aβ assessed by multivariable linear regression, we assumed regression coefficients of 8.24 (Aβ40) and 10.51 (Aβ42) for NAFLD and regression coefficients of −8.87 (Aβ40) and −12.12 (Aβ42) for CT + TT genotypes. Assuming a two-tailed alpha level of 0.05, our study had 80% power to determine interaction regression coefficients of at least −9.29 for plasma Aβ40 and -15.32 for Aβ42.

All analyses were conducted using the SAS software (version 9.4; SAS Institute, Inc., Cary, NC, USA). A two-tailed *P* value below 0.05 was considered statistically significant.

### Role of the funding source

The funding sources had no role in the design of study, collection, analysis, and interpretation of data, preparation of the manuscript, or decision to submit the paper for publication.

## Results

### Rats with NAFLD exhibited cognitive dysfunction

**Fig. 1:**
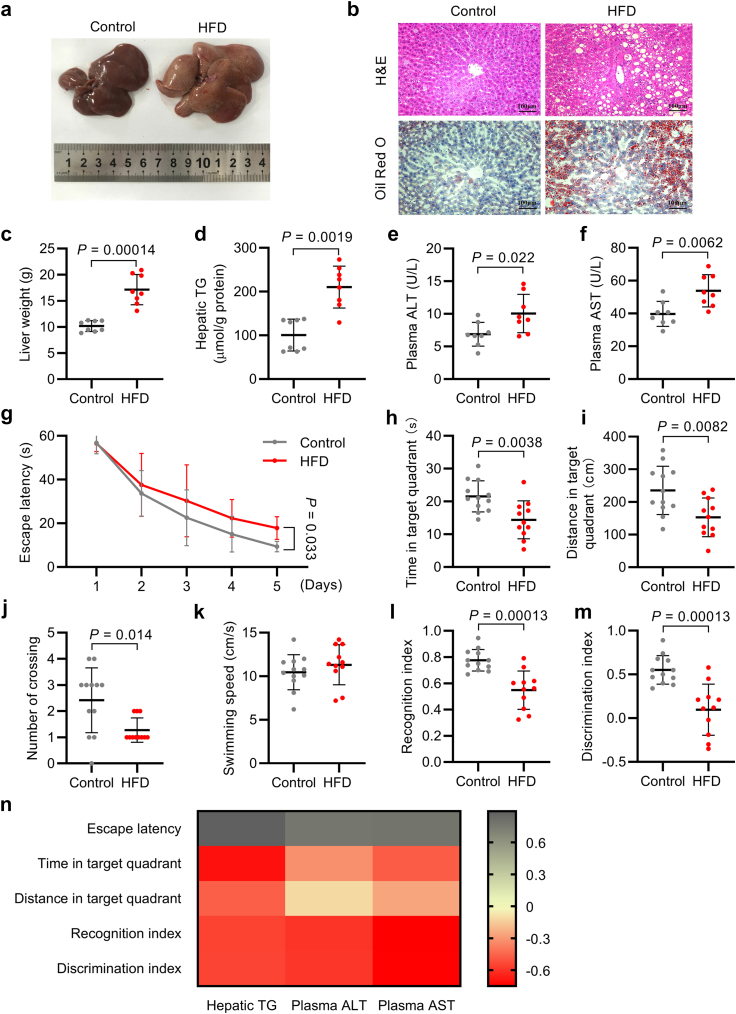
HFD caused NAFLD and cognitive impairment in rats. (a) Representative image of liver from control and HFD-fed rats. (b) H&E and Oil Red O staining for liver tissue of control and HFD-fed rats (*n* = 3–4 per group); scale bars indicate 100 μm. (c–f) Liver weight (c), hepatic TG (d), plasma ALT (e), and plasma AST (f) of control and HFD-fed rats (*n* = 8 per group). (g) Escape latency during the training phase in MWM test of control and HFD-fed rats. (h–k) Time spent in the target quadrant (h), swimming distance in the target quadrant (i), number of crossing the platform (j), and swimming speed (k) during the probe trial in MWM test of control and HFD-fed rats (*n* = 11–12 per group). (l–m) Recognition index (l) and discrimination index (m) in NOR test of control and HFD-fed rats (*n* = 11–12 per group). (n) Heatmap for correlations of hepatic TG content and liver function markers with cognitive performances by Spearman test; the intensity of colour represents Spearman correlation coefficient (*n* = 8 per group). Data were presented as mean ± SD. *P*-values were calculated by unpaired Student’s *t*-test (e, f, h, i, and k–m), Welch’s *t*-test (c), Mann–Whitney *U* test (j), or Scheirer-Ray-Hare test (g).

To confirm the induction of NAFLD by HFD, we examined liver tissues and observed evident hepatomegaly, hepatosteatosis, and a 69% increase in liver weight among HFD-fed rats (Fig. 1a–c and Supplementary Table S1). Hepatic TG levels, a major constituent of lipid droplets, exhibited a 116% increase in HFD-fed rats compared to those in the control group (Fig. 1d and Supplementary Table S1). Additionally, plasma ALT and AST levels, biomarkers for liver injury, were elevated in HDF-fed rats (Fig. 1e and f and Supplementary Table S1).

MWM and NOR tests were conducted to assess the alterations in cognitive function. In the MWM test, rats fed on HFD exhibited a prolonged escape latency during the training phase, indicating a decline in spatial learning ability (*P* = 0.033 for treatment, *P* < 0.0001 for training time, *P* = 0.46 for interaction; Scheirer-Ray-Hare test) (Fig. 1g). The HFD group also displayed reduced spatial memory, as evidenced by the decreased time and distance spent in the target quadrant, diminished number of platform crossings, and no significant alteration in swimming speed during the probe trial (Fig. 1h–k and Supplementary Table S1). In the NOR test, HFD-fed rats demonstrated lower recognition and discrimination indices, indicating impaired working memory (Fig. 1l and m and Supplementary Table S1). Additionally, open field and elevated plus-maze tests were conducted to rule out changes in spontaneous locomotor activity and anxiety-like behaviour. There was no significant difference between the two groups (Supplementary Fig. S5).

We further evaluated the correlation between the severity of hepatic steatosis and dysfunction and cognitive performance (Fig. 1n, Supplementary Table S1). Hepatic TG content exhibited negative correlations with spatial learning and memory as well as working memory. Meanwhile, plasma ALT and AST levels were inversely correlated with spatial learning ability and working memory. These findings suggest that NAFLD promotes the development of cognitive impairment.

### NAFLD was positively associated with cerebral and/or plasma Aβ accumulation in rats and human subjects

**Fig. 2:**
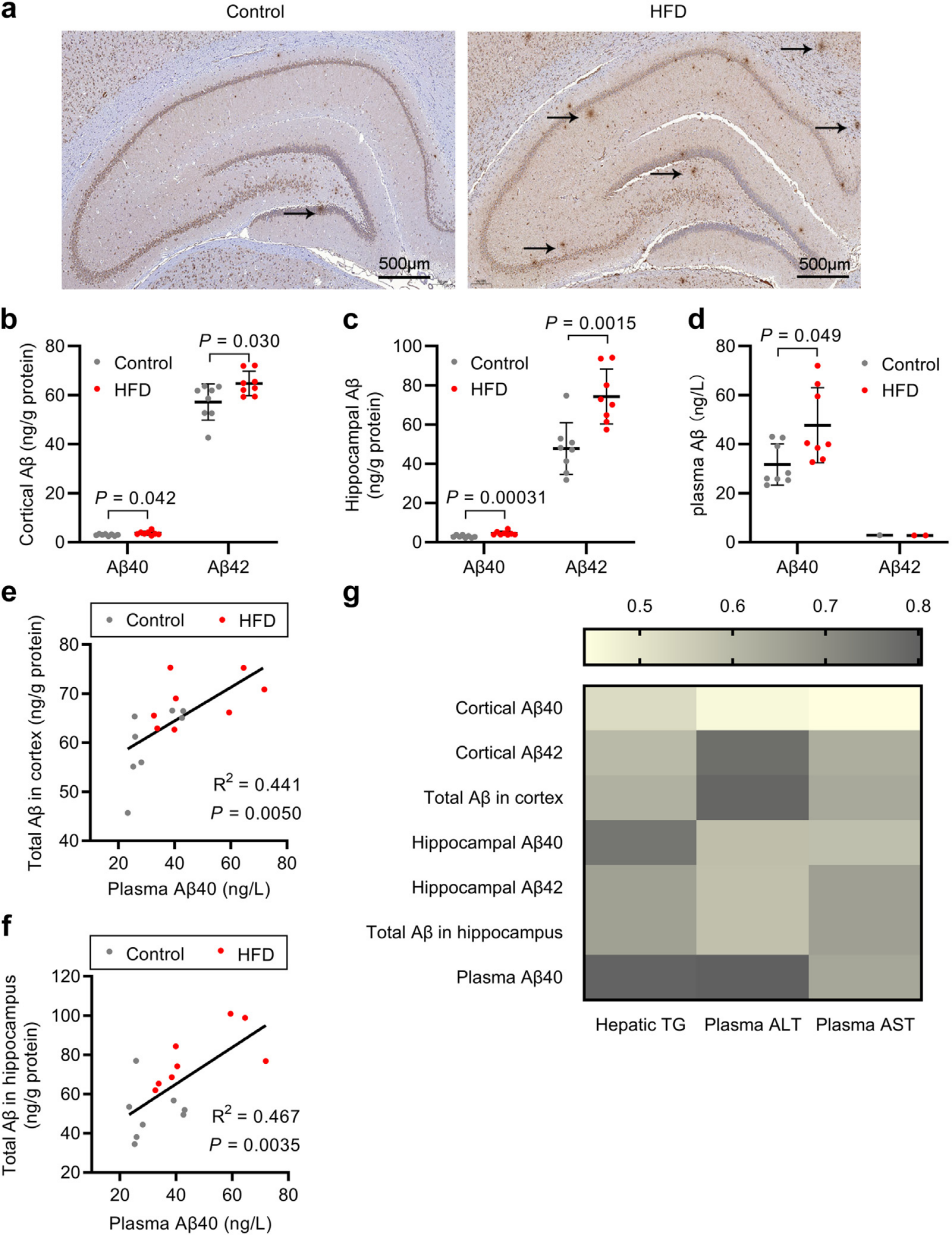
HFD-induced NAFLD was positively correlated with Aβ accumulation in rats. (a) Representative image of immunohistochemistry for Aβ in brain tissue of control and HFD-fed rats by 6E10 antibody (*n* = 3–4 per group); arrows indicate Aβ deposition in brain tissue. (b, c) Aβ40 and Aβ42 levels in the cortex (b) and hippocampus (c) of control and HFD-fed rats. (d) Plasma Aβ40 and Aβ42 levels of control and HFD-fed rats; seven rats in the control group and six rats in HFD group had plasma Aβ42 levels below the lower limit of detection. (e, f) Linear regression between plasma Aβ40 and total Aβ levels in the cortex (e) and hippocampus (f) in control and HFD-fed rats; the regression lines were fitted by ordinary least square. (g) Heatmap for correlations of hepatic TG content and liver function markers with cerebral and plasma Aβ levels by Spearman test; the intensity of colour represents Spearman correlation coefficient. *n* = 8 per group unless otherwise stated. Data were presented as mean ± SD. *P*-values were calculated by unpaired Student’s *t*-test (b–d) except for Aβ40 in the hippocampus (c) and plasma (d), which were compared with the Mann–Whitney *U* test.

Aβ accumulation in the brain is one of the important pathologies of cognitive impairment. Immunohistochemistry revealed exacerbation of Aβ deposition in the cortex and hippocampus of HFD-fed rats (Fig. 2a). Through quantification of Aβ levels, we consistently observed elevated concentrations of Aβ40 and Aβ42 in the cortex and hippocampus of HFD-fed rats (Fig. 2b and c, Supplementary Table S1). As expected, the cortical and hippocampal Aβ concentrations were negatively associated with cognitive function (Supplementary Table S2). However, the expression of APP, IDE, and NEP, integral to the Aβ production and degradation pathways, remained insignificantly altered in HFD-fed rats (Supplementary Fig. S6).

Considering the pivotal role of the peripheral system in Aβ clearance, we assessed plasma Aβ levels and noted increased plasma Aβ40 concentrations in HFD-fed rats (Fig. 2d, Supplementary Table S1). Notably, plasma Aβ40 levels exhibited positive correlations with total Aβ content in both the cortex (R^2^ = 0.441, *P* = 0.0050; linear regression) and hippocampus (R^2^ = 0.467, *P* = 0.0035; linear regression) (Fig. 2e and f, Supplementary Table S1). Subsequently, we determined the correlations between the severity of hepatic steatosis and dysfunction and cerebral and plasma Aβ accumulation (Fig. 2g, Supplementary Table S1). The results demonstrated that hepatic TG content as well as plasma ALT and AST levels were positively correlated with Aβ concentrations in the cortex, hippocampus, and plasma.

Next, we performed a case–control study, including 549 cases with NAFLD and 549 controls, to assess the relationship between NAFLD and plasma Aβ levels in human subjects. The characteristics of the participants are presented in Table 1. Compared to controls, individuals with NAFLD had higher levels of BMI, TG, total cholesterol, ALT, creatinine, and plasma Aβ40 and Aβ42 as well as lower eGFR levels. NAFLD cases were also more likely to have diabetes, hyperlipidaemia, and hypertension. After multivariate adjustment, NAFLD was associated with an elevation of 8.24% (95% CI 5.60%, 10.88%) and 10.51% (95% CI 6.25%, 14.77%) in plasma Aβ40 and Aβ42, respectively (Table 2). Each 10 U/L increment in ALT was associated with an elevation of 1.00% (95% CI 0.28%, 1.73%) and 2.14% (95% CI 0.97%, 3.30%) in plasma Aβ40 and Aβ42, respectively (Table 2). Seventy NAFLD cases were classified as moderate or severe and displayed a 6.39% (95% CI 0.75%, 12.03%) increment in plasma Aβ40 and 9.23% (95% CI 0.10%, 18.36%) increment in plasma Aβ42 compared to those classified as mild (Table 2, Supplementary Table S3). Additionally, 200 NAFLD cases with a history of diagnosed NAFLD exhibited a 7.82% (95% CI 1.59%, 14.06%) increment in plasma Aβ42 compared to those without a history of diagnosed NAFLD (Table 2, Supplementary Table S4).

**Table 1:** Demographic and clinical characteristics of the NAFLD cases and matched controls.

Characteristics	Cases (*n* = 549)	Controls (*n* = 549)	*P*
Age (years)	60 (45, 64)	59 (45, 63)	0.41
Sex			1.0
Male, *n* (%)	391 (71.2)	391 (71.2)	
Female, *n* (%)	158 (28.8)	158 (28.8)	
BMI (kg/m^2^)	25.90 (2.73)	22.96 (2.61)	<0.0001
Current smoker, *n* (%)	186 (33.9)	188 (34.2)	0.90
Current drinker, *n* (%)	137 (25.0)	105 (19.1)	0.020
Physical activity, *n* (%)	229 (41.7)	239 (43.5)	0.54
Diabetes, *n* (%)	51 (9.3)	20 (3.6)	0.00014
Hyperlipidaemia, *n* (%)	275 (50.1)	131 (23.9)	<0.0001
Hypertension, *n* (%)	163 (29.7)	98 (17.9)	<0.0001
CVD, *n* (%)	37 (6.7)	38 (6.9)	0.90
TG (mmol/L)	1.67 (1.23–2.39)	1.20 (0.86–1.62)	<0.0001
Total cholesterol (mmol/L)	5.00 (4.34–5.67)	4.53 (4.02–5.10)	<0.0001
LDL cholesterol (mmol/L)	2.59 (1.80–3.25)	2.47 (1.83–3.01)	0.061
HDL cholesterol (mmol/L)	1.29 (1.15–1.47)	1.33 (1.15–1.53)	0.072
ALT (U/L)	24 (19–32)	18 (15–23)	<0.0001
Creatinine (μmol/L)	72.8 (62.0–83.7)	70.8 (60.1–80.2)	0.0055
eGFR (mL/min/1.73 m^2^)	96.69 (85.22–109.76)	99.81 (89.62–110.45)	0.0017
Aβ40 (ng/L)	140.05 (122.58–163.41)	126.58 (115.90–141.19)	<0.0001
Aβ42 (ng/L)	13.97 (10.73–17.46)	12.18 (10.33–14.21)	<0.0001

Data were presented as mean (SD) for normally distributed data, median (interquartile range) for non-normally distributed data, or *n* (%) for categorical data.

**Table 2:** Mean % (95% confidence interval) difference in plasma Aβ40 and Aβ42 levels associated with NAFLD-related variates.

Variates	Aβ40			Aβ42		
	Model 1	Model 2	Model 3	Model 1	Model 2	Model 3
NAFLD						
No (*n* = 549)	0.00 (ref.)	0.00 (ref.)	0.00 (ref.)	0.00 (ref.)	0.00 (ref.)	0.00 (ref.)
Yes (*n* = 549)	8.97 (6.71, 11.22)	8.29 (5.65, 10.93)	8.24 (5.60, 10.88)	11.86 (8.23, 15.48)	10.42 (6.17, 14.67)	10.51 (6.25, 14.77)
*P*	<0.0001	<0.0001	<0.0001	<0.0001	<0.0001	<0.0001
ALT						
Per 10 U/L increment	1.46 (0.75, 2.17)	1.06 (0.37, 1.79)	1.00 (0.28, 1.73)	2.59 (1.46, 3.71)	2.12 (0.96, 3.28)	2.14 (0.97, 3.30)
*P*	<0.0001	0.0042	0.0070	<0.0001	0.00035	0.00033
Severity of NAFLD^a^						
Mild (*n* = 485)	0.00 (ref.)	0.00 (ref.)	0.00 (ref.)	0.00 (ref.)	0.00 (ref.)	0.00 (ref.)
Moderate or severe (*n* = 64)	7.32 (1.71, 12.92)	6.72 (1.08, 12.36)	6.39 (0.75, 12.03)	10.47 (1.56, 19.37)	9.23 (0.14, 18.33)	9.23 (0.10, 18.36)
*P*	0.011	0.020	0.027	0.022	0.047	0.048
History of diagnosed NAFLD^a^						
No (*n* = 370)	0.00 (ref.)	0.00 (ref.)	0.00 (ref.)	0.00 (ref.)	0.00 (ref.)	0.00 (ref.)
Yes (*n* = 179)	2.85 (−1.03, 6.73)	2.93 (−0.90, 6.77)	2.34 (−1.53, 6.21)	7.77 (1.64, 13.91)	7.74 (1.59, 13.90)	7.82 (1.59, 14.06)
*P*	0.15	0.13	0.24	0.013	0.014	0.014

Model 1 was adjusted for age and sex. Model 2 was additionally adjusted for BMI, current smoking status, current drinking status, physical activity, TG, total cholesterol, and eGFR. Model 3 was additionally adjusted for history of diabetes, history of hypertension, history of CVD.

a The analyses were conducted among NAFLD cases.

Taken together, these data indicate that NAFLD may accelerate cerebral Aβ accumulation by reducing peripheral Aβ clearance.

### NAFLD reduced LRP-1-mediated Aβ uptake in rat liver and cultured hepatocyte

**Fig. 3:**
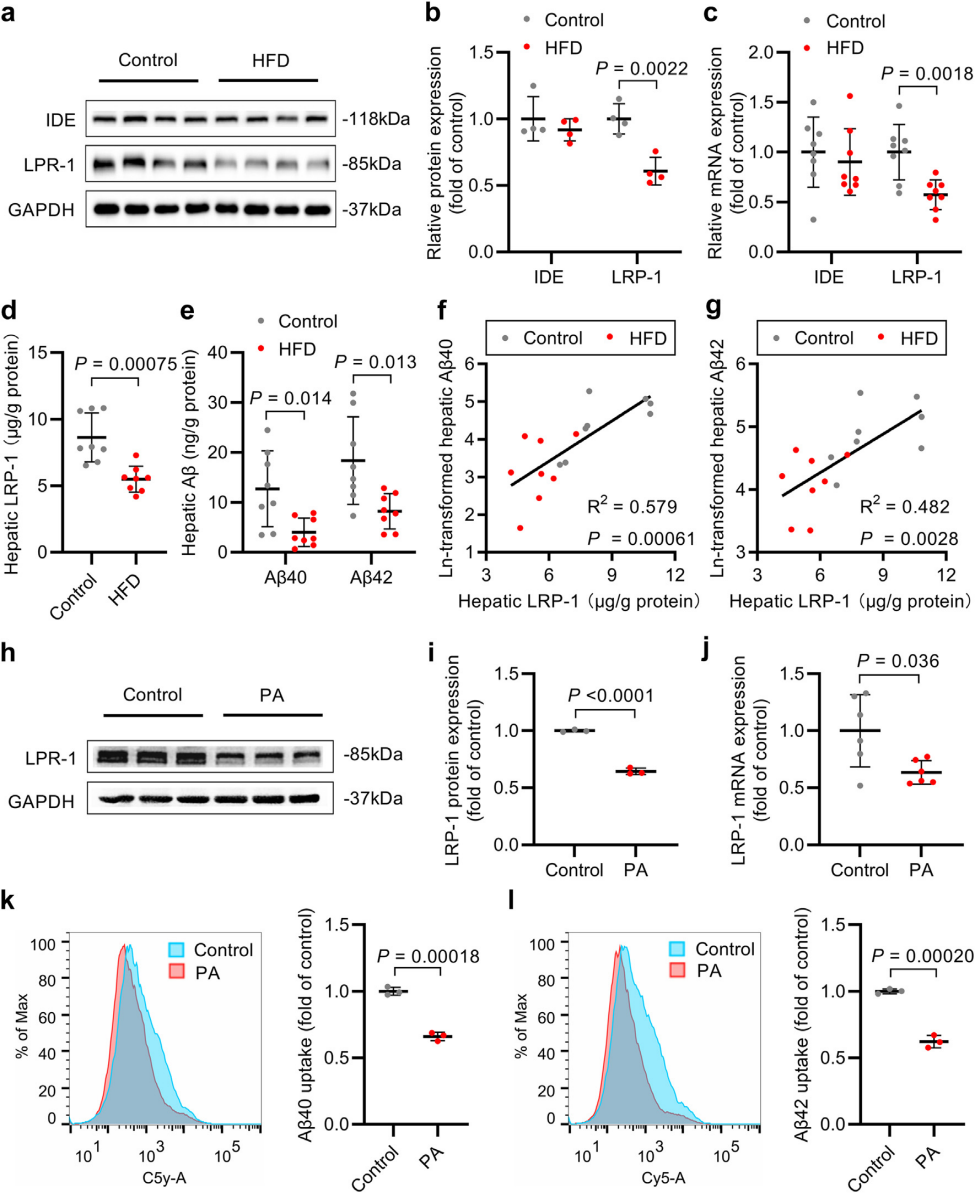
NAFLD reduced LRP-1-mediated Aβ uptake in vivo and in vitro. (a, b) Western blot bands (a) and quantification (b) of IDE and LRP-1 protein expression in the liver of control and HFD-fed rats (*n* = 4 per group). (c) Real-time PCR quantification of IDE and LRP-1 mRNA expression in the liver of control and HFD-fed rats (*n* = 8 per group). (d, e) Hepatic LRP-1 (d) and Aβ40 and Aβ42 (e) levels of control and HFD-fed rats (*n* = 8 per group). (f, g) Linear regression between hepatic LRP-1 and logarithmically transformed hepatic Aβ40 (f) and Aβ42 (g) in control and HFD-fed rats; the regression lines were fitted by ordinary least square (*n* = 8 per group). (h, i) Western blot bands (h) and quantification (i) of IDE and LRP-1 protein expression in HepG2 cells treated with or without 400 μM PA (*n* = 3 per group). (j) Real-time PCR quantification of IDE and LRP-1 mRNA expression in HepG2 cells treated with or without 400 μM PA (*n* = 6 per group). (k, l) Flow fluorescence intensity histogram and quantification of Aβ40 (k) and Aβ42 (l) uptake in HepG2 cells treated with or without 400 μM PA (*n* = 3 per group). Data were presented as mean ± SD. *P*-values were calculated by unpaired Student’s *t*-test (b–d, i, k, l) or Welch’s *t*-test (e, j) except for IDE protein in the liver (b), which was compared with the Mann–Whitney *U* test.

LRP-1 and IDE play integral roles in Aβ uptake and degradation in the liver, respectively. HFD led to a 39% and 43% decrease in LRP-1 protein and mRNA expression, whereas the expression of IDE remained unaltered in the rat liver (Fig. 3a–c, Supplementary Table S1), indicating an impairment in hepatic Aβ uptake capability. Consequently, our quantitative findings demonstrated a noteworthy reduction in LRP-1, Aβ40, and Aβ42 levels in the livers of HFD-fed rats (Fig. 3d and e, Supplementary Table S1). Hepatic LRP-1 levels exhibited positive correlations with hepatic Aβ40 (R^2^ = 0.579, *P* = 0.00061; linear regression) and Aβ42 (R^2^ = 0.482, *P* = 0.0028; linear regression) levels among all rats (Fig. 3f and g, Supplementary Table S1). Furthermore, hepatic LRP-1 and Aβ levels were inversely correlated with Aβ concentrations in the cortex, hippocampus, and plasma as well as cognitive performance (Supplementary Table S5). To validate the impact of NAFLD on Aβ uptake, we treated HepG2 cells with PA and observed a 35% and 37% reduction in LPR-1 protein and mRNA expression (Fig. 3h–j, Supplementary Table S1). Concurrently, the uptake capability of Aβ40 and Aβ42 was decreased by 34% and 38%, respectively, in HepG2 cells exposed to PA (Fig. 3k and l, Supplementary Table S1). These data reveal an adverse effect of NAFLD on hepatic Aβ clearance via inhibition of LRP-1 expression.

### PPARα mediated the adverse effect of NAFLD on LRP-1-mediated Aβ uptake

**Fig. 4:**
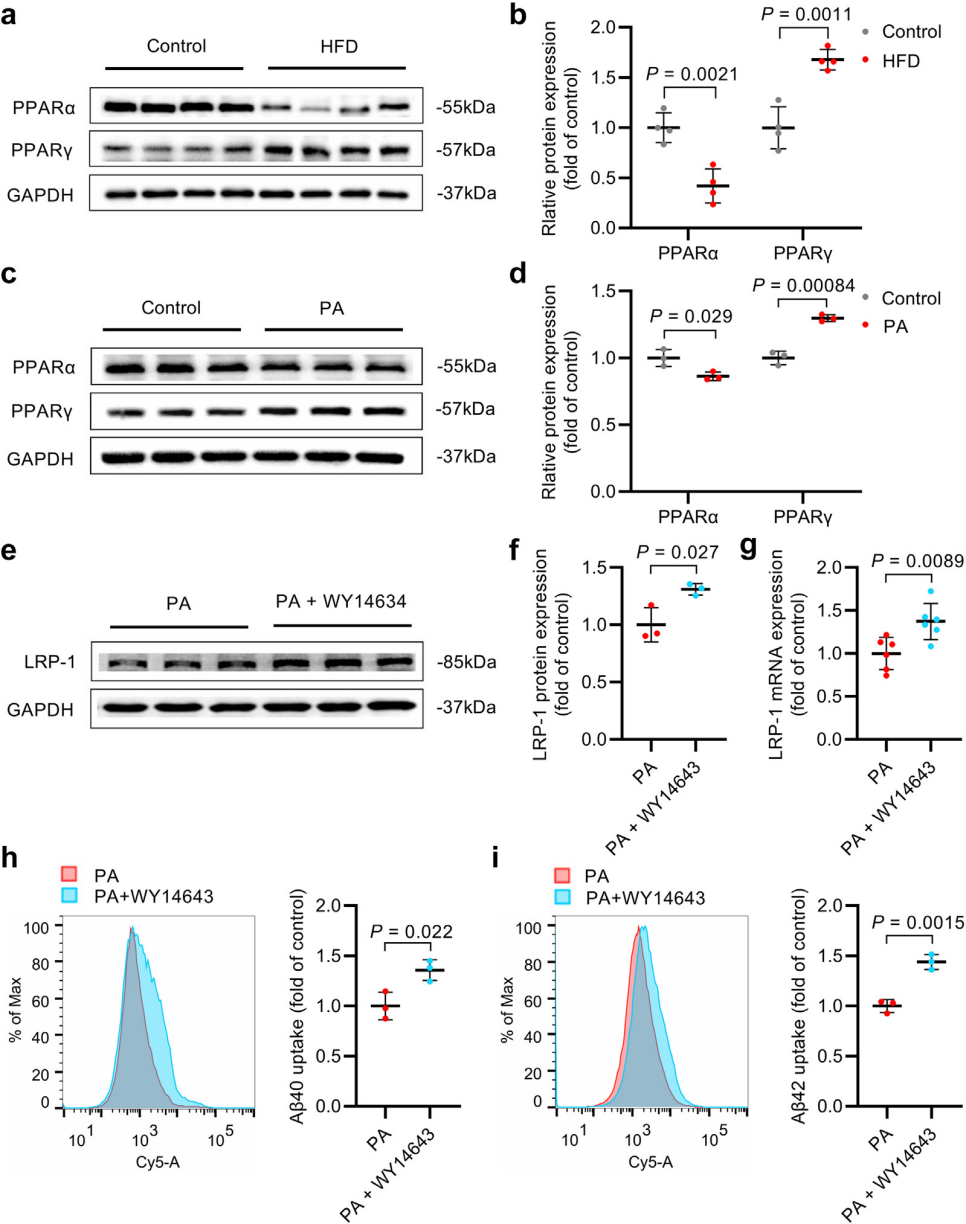
Down-regulation of PPARα mediated the reduction of LRP-1 expression and Aβ uptake in NAFLD. (a, b) Western blot bands (a) and quantification (b) of PPARα and PPARγ protein expression in the liver of control and HFD-fed rats (*n* = 4 per group). (c, d) Western blot bands (c) and quantification (d) of PPARα and PPARγ protein expression in HepG2 cells treated with or without 400 μM PA (*n* = 3 per group). (e, f) Western blot bands (e) and quantification (f) of LRP-1 protein expression in HepG2 cells treated with 400 μM PA and with or without 50 μM WY14643 (*n* = 3 per group). (g) Real-time PCR quantification of LRP-1 mRNA expression in HepG2 cells treated with 400 μM PA and with or without 50 μM WY14643 (*n* = 6 per group). (h, i) Flow fluorescence intensity histogram and quantification of Aβ40 (h) and Aβ42 (i) uptake in HepG2 cells treated with 400 μM PA and with or without 50 μM WY14643 (*n* = 3 per group). Data were presented as mean ± SD. *P*-values were calculated by unpaired Student’s *t*-test.

Considering the presence of PPARs binding sites within the *LRP-1* promoter region, we investigated the expression of PPARs in both in vivo and in vitro NAFLD models. In the liver of rats, HFD led to a reduction in PPARα protein expression and an increase in PPARγ protein expression (Fig. 4a and b, Supplementary Table S1). These trends were in line with the findings from PA-treated HepG2 cells, in which PPARα was downregulated and PPARγ was upregulated (Fig. 4c and d, Supplementary Table S1). To further verify the regulation of LRP-1 by PPARs, we treated HepG2 cells with 50 μM WY14643 (PPARα agonist) or 15 μM T0070907 (PPARγ inhibitor). These treatments effectively modulated the expression of targeted proteins without compromising cell viability (Supplementary Fig. S7). T0070907 did not increase the expression of LRP-1 mRNA and even led to a decrease in LRP-1 protein expression (Supplementary Fig. S8). In contrast, WY14643 notably elevated both LRP-1 protein and mRNA levels in PA-treated HepG2 cells (Fig. 4e–g, Supplementary Table S1). Furthermore, administration of WY14643 substantially increased Aβ40 and Aβ42 uptake by 36% and 44%, respectively, in PA-treated HepG2 cells (Fig. 4h and i, Supplementary Table S1). These findings demonstrate that the reduction in PPARα contributes to impaired Aβ uptake capability in NAFLD.

### Interaction between NAFLD and *LRP-1* rs1799986 polymorphism on plasma Aβ in human subjects

The minor allele frequency (T allele) was 7.7% among all participants (Supplementary Table S6). In multivariate linear regression, the T allele was associated with a 7.98% (95% CI 4.90%, 11.06%) reduction in plasma Aβ40 and 11.48% (95% CI 6.52%, 16.43%) reduction in plasma Aβ42 compared with the C allele. Compared with the CC genotype, the CT + TT genotypes were associated with a reduction of 8.87% (95% CI 5.66%, 12.09%) and 12.12% (95%CI 6.94%, 17.31%) in plasma Aβ40 and Aβ42, respectively.

We further examined the interaction between NAFLD and rs1799986 polymorphism in association with plasma Aβ. The positive associations between NAFLD and plasma Aβ40 and Aβ42 were modified by rs1799986 genotypes. The associations between NAFLD and plasma Aβ40 and Aβ42 were weaker in individuals carrying the T allele than in those not carrying the T allele (*P* for interaction = 0.0017 and 0.0015, respectively; likelihood ratio test) (Table 3). Meanwhile, the genetic effects of the T allele on plasma Aβ40 and Aβ42 levels were augmented by NAFLD (Supplementary Table S7). These results suggest that LRP-1 potentially mediates the unfavourable effects of NAFLD on peripheral Aβ clearance in humans.

**Table 3:** Mean % (95% confidence interval) difference in plasma Aβ40 and Aβ42 levels associated with NAFLD according to rs1799986 genotypes.

Genotypes	NAFLD			Regression coefficients (95% CI) for interaction term	*P* for interaction
	No	Yes	*P*		
Aβ40					
CC	0.00 (ref.)	9.74 (6.82, 12.65)	<0.0001	−10.05 (−16.29, −3.80)	0.0017
TC + TT	0.00 (ref.)	2.00 (−2.90, 6.89)	0.43		
Aβ42					
CC	0.00 (ref.)	12.54 (7.82, 17.27)	<0.0001	−16.46 (−26.59, −6.33)	0.0015
TC + TT	0.00 (ref.)	2.45 (−6.16, 11.06)	0.58		

Multivariable analysis was adjusted for age, sex, BMI, current smoking status, current drinking status, physical activity, history of diabetes, history of hypertension, history of CVD, TG, total cholesterol, and eGFR.

## Discussion

In this study, we elucidated positive correlations between NAFLD, cerebral Aβ accumulation, and cognitive dysfunction in rats. Meanwhile, NAFLD was linked to elevated plasma Aβ levels in rats and humans, indicating compromised peripheral Aβ clearance. Mechanistically, NAFLD impeded LRP-1-mediated Aβ uptake by the liver, which was attributed to the reduction of PPARα. As a consequence, the PPARα agonist restored the compromised Aβ uptake capacity caused by NAFLD. Furthermore, the positive association between NAFLD and plasma Aβ was modified by the *LRP-1* rs1799986 genotype in humans. Taken together, our results underscore the potential mechanism of NAFLD in promoting cognitive impairment by impeding LRP-1-mediated peripheral Aβ clearance via the liver.

An increasing body of literature highlights the pivotal role of peripheral Aβ clearance in the development of cognitive dysfunction.^30^ Our findings reveal a deleterious effect of NAFLD on peripheral Aβ clearance. We unveiled a positive association between NAFLD and plasma Aβ levels in humans. Our in vivo and in vitro results provided further evidence of the deleterious influence of NAFLD on hepatic Aβ uptake capacity. Similarly, elevated plasma Aβ levels have been demonstrated in patients with cirrhosis or hepatocellular carcinoma which is the adverse outcome related to NAFLD.^15^^,^^16^ In patients with cirrhosis, circulating Aβ cannot be eliminated by the liver.^31^ Moreover, previous experimental studies have indicated that reduced peripheral Aβ clearance exacerbates Aβ accumulation in the CNS.^32^^,^^33^ Consistent with these studies, we found that reduced peripheral Aβ clearance due to NAFLD was related to cerebral Aβ accumulation, which was negatively correlated with cognitive performance. Therefore, impaired peripheral Aβ clearance serves as a potential explanation for the epidemiological association between NAFLD and cognitive dysfunction. Unlike our study, a previous study found that lower levels of ALT were associated with increased Aβ deposition.^17^ However, this previous study did not consider the causes of altered liver enzymes, and ALT was used as a biomarker for liver function rather than liver injury. In the present study, we excluded participants with liver disease other than NAFLD and adjusted for confounders associated with altered ALT, such as drinking status, physical activity, and history of CVD.^34^ The positive association between ALT and plasma Aβ may reflect the relationship between chronic liver injury and Aβ accumulation. Additionally, encouraging peripheral Aβ clearance has been reported to diminish cerebral Aβ deposition and improve cognitive impairment in AD mice.^11^^,^^35^ This could also be a potential strategy for alleviating NAFLD-induced cognitive impairment. Notably, not all cognitive impairment in NAFLD patients is attributed to amyloid pathology, although we demonstrate a role of peripheral Aβ clearance in NAFLD-induced cognitive impairment. Encouraging peripheral Aβ clearance may be only appropriate for NAFLD patients with both cognitive impairment and amyloid pathology.

Previous studies have shown a protective effect of the T allele of *LRP-1* rs1799986 on cerebral amyloid angiopathy, cognitive impairment, and AD.^36^, ^37^, ^38^ In this study, we found that the rs1799986 T allele is linked to reduced plasma Aβ levels in humans. This discovery, in conjunction with earlier research,^36^ suggests that the protective effect of the rs1799986 T allele against cognitive impairment and AD may involve modulation of peripheral Aβ clearance. The liver is the main site of LRP-1 involved in peripheral Aβ clearance, and hepatocyte-specific *LRP-1* knockdown has been demonstrated to accelerate cerebral Aβ accumulation and cognitive dysfunction in AD mice.^32^ Similarly, we observed diminished hepatic LRP-1 and Aβ uptake in NAFLD, a phenomenon inversely associated with plasma Aβ levels and cerebral Aβ accumulation. Consistently, the positive correlation between NAFLD and plasma Aβ was notably attenuated among individuals carrying the rs1799986 T allele, a subgroup characterized by higher LRP-1 expression compared to carriers of the C allele.^39^ These findings underscore the role of LRP-1 in mediating the deleterious effects of NAFLD on peripheral Aβ clearance. Importantly, previous studies have demonstrated that augmenting hepatic LRP-1 expression through pharmacological intervention or overexpression strategy mitigates cerebral Aβ deposition and enhances cognitive function in AD mice.^32^^,^^40^ Whether enhancing hepatic LRP-1 expression can ameliorate Aβ accumulation and cognitive dysfunction induced by NAFLD warrants further investigation.

PPARα has emerged as a link between lipid metabolism and cognitive function^41^^,^^42^; however, almost all studies have focused on PPARα signalling within the CNS. In the present study, we found that attenuation of PPARα in NAFLD precipitated a diminution in hepatic LRP-1 expression, culminating in compromised hepatic Aβ clearance. This observation provides insight into the role of hepatic PPARα signalling in cognitive function. PPARs binding sites have been identified within the LRP-1 promoter region.^43^ Previous studies have demonstrated that activating PPARγ can up-regulate the expression of LRP-1 by enhancing the transcriptional activity of the LRP-1 promoter.^22^^,^^44^ Similarly, we found that PPARα agonist could increase the transcription of LRP-1. Our results, combined with previous studies, suggest that PPARα may regulate LRP-1 expression by promoting LRP-1 promoter activity. In AD mice, PPARα agonists have been shown to reverse memory deficits by promoting Aβ clearance.^45^ However, the specific role of hepatic PPARα signalling needs to be further verified.

There are several limitations to our study. First, although we established a correlation between NAFLD and Aβ accumulation in rats and humans, clearance of Aβ in the CNS and peripheral system needs to be further determined in vivo using quantitative methods (e.g., radiolabeled Aβ). Second, we found that NAFLD hindered the hepatic uptake of Aβ via the PPARα/LRP-1 pathway, but studies in hepatocyte-specific *LRP-1* or *PPARα* overexpression animal models challenged with a long-term HFD are required to clarify its role in NAFLD-induced Aβ pathology and cognitive impairment. Third, although many confounders have been taken into consideration in our population study, we could not rule out the possibility of unmeasured confounding and residual confounding due to measurement error in confounders. Meanwhile, we could not rule out the potential mediating effect of adjustment factors due to the case–control study design. Finally, cognitive function was not assessed in this population-based study. We plan to further investigate the mediating effect of plasma Aβ in the prospective association of NAFLD with cognitive impairment in the Shenzhen Ageing Cohort Study.^46^

In conclusion, our study highlights the importance of peripheral Aβ clearance in NAFLD-induced cognitive impairment. Reduction in hepatic LRP-1 regulated by PPARα contributes to impaired peripheral Aβ clearance, thereby leading to cerebral Aβ accumulation and cognitive impairment in NAFLD. Therefore, enhancing peripheral Aβ clearance may be a potential therapy for NAFLD-induced cognitive dysfunction and hepatic LRP-1 may be a key target for intervention.

## Contributors

XP, JX, and LGL conceived and designed the study. XP, ZX, LYL, XM, and ZP performed the in vivo and in vitro experiments. XP, XZ, ZX, and ZS conducted the population-based study. XP and ZX contributed to data analysis. XP, XZ, ZX, LYL, XM, ZP, ZS, HY, JX, and LGL contributed to the acquisition and interpretation of data. XP wrote the draft of the paper. XZ, ZX, LYL, XM, ZP, ZS, HY, JX, and LGL contributed to reviewing and revising the paper. All authors have read and approved the final manuscript. XP, ZX, and LGL have verified the underlying data. All authors confirm that they had full access to all the data in the study and accept responsibility to submit the paper for publication.

## Data sharing statement

The data supporting the findings of this study are available from the corresponding author upon reasonable request.

## Declaration of interests

All authors have declared that no competing interests exist.

## Appendix ASupplementary data

Supplementary Figs. S1–S8 and Tables S1–S7

Supplemental Western blots

Reagent Validation

## References

[1] Powell E.E., Wong V.W., Rinella M. (2021). Non-alcoholic fatty liver disease. Lancet.

[2] Targher G., Tilg H., Byrne C.D. (2021). Non-alcoholic fatty liver disease: a multisystem disease requiring a multidisciplinary and holistic approach. Lancet Gastroenterol Hepatol.

[3] Armstrong M.J., Adams L.A., Canbay A., Syn W.K. (2014). Extrahepatic complications of nonalcoholic fatty liver disease. Hepatology.

[4] Seo S.W., Gottesman R.F., Clark J.M. (2016). Nonalcoholic fatty liver disease is associated with cognitive function in adults. Neurology.

[5] Liu Q., Liu C., Hu F., Deng X., Zhang Y. (2022). Non-alcoholic fatty liver disease and longitudinal cognitive changes in middle-aged and elderly adults. Front Med.

[6] Kim G.A., Oh C.H., Kim J.W. (2022). Association between non-alcoholic fatty liver disease and the risk of dementia: a nationwide cohort study. Liver Int.

[7] Jeong S., Oh Y.H., Choi S. (2022). Association of non-alcoholic fatty liver disease with incident dementia later in life among elder adults. Clin Mol Hepatol.

[8] Ossenkoppele R., Jansen W.J., Rabinovici G.D. (2015). Prevalence of amyloid PET positivity in dementia syndromes: a meta-analysis. JAMA.

[9] Irwin D.J., Lee V.M., Trojanowski J.Q. (2013). Parkinson’s disease dementia: convergence of α-synuclein, tau and amyloid-β pathologies. Nat Rev Neurosci.

[10] Ruan D., Sun L. (2023). Amyloid-β PET in Alzheimer’s disease: a systematic review and Bayesian meta-analysis. Brain Behav.

[11] Xiang Y., Bu X.L., Liu Y.H. (2015). Physiological amyloid-beta clearance in the periphery and its therapeutic potential for Alzheimer’s disease. Acta Neuropathol.

[12] Roberts K.F., Elbert D.L., Kasten T.P. (2014). Amyloid-beta efflux from the central nervous system into the plasma. Ann Neurol.

[13] Qosa H., Abuasal B.S., Romero I.A. (2014). Differences in amyloid-β clearance across mouse and human blood-brain barrier models: kinetic analysis and mechanistic modeling. Neuropharmacology.

[14] Ghiso J., Shayo M., Calero M. (2004). Systemic catabolism of alzheimer's Abeta40 and Abeta42. J Biol Chem.

[15] Wang Y.R., Wang Q.H., Zhang T. (2017). Associations between hepatic functions and plasma amyloid-beta levels-Implications for the capacity of liver in peripheral amyloid-beta clearance. Mol Neurobiol.

[16] Jin W.S., Bu X.L., Liu Y.H. (2017). Plasma amyloid-beta levels in patients with different types of cancer. Neurotox Res.

[17] Nho K., Kueider-Paisley A., Ahmad S. (2019). Association of altered liver enzymes with Alzheimer disease diagnosis, cognition, neuroimaging measures, and cerebrospinal fluid biomarkers. JAMA Netw Open.

[18] Kim D.G., Krenz A., Toussaint L.E. (2016). Non-alcoholic fatty liver disease induces signs of Alzheimer’s disease (AD) in wild-type mice and accelerates pathological signs of AD in an AD model. J Neuroinflammation.

[19] Sagare A.P., Deane R., Zlokovic B.V. (2012). Low-density lipoprotein receptor-related protein 1: a physiological Aβ homeostatic mechanism with multiple therapeutic opportunities. Pharmacol Ther.

[20] Shinohara M., Tachibana M., Kanekiyo T., Bu G. (2017). Role of LRP1 in the pathogenesis of Alzheimer’s disease: evidence from clinical and preclinical studies. J Lipid Res.

[21] Tamaki C., Ohtsuki S., Iwatsubo T. (2006). Major involvement of low-density lipoprotein receptor-related protein 1 in the clearance of plasma free amyloid beta-peptide by the liver. Pharm Res.

[22] Kim H.J., Moon J.H., Kim H.M. (2014). The hypolipidemic effect of cilostazol can be mediated by regulation of hepatic low-density lipoprotein receptor-related protein 1 (LRP1) expression. Metabolism.

[23] Gali C.C., Fanaee-Danesh E., Zandl-Lang M. (2019). Amyloid-beta impairs insulin signaling by accelerating autophagy-lysosomal degradation of LRP-1 and IR-β in blood-brain barrier endothelial cells in vitro and in 3XTg-AD mice. Mol Cell Neurosci.

[24] Salinero A.E., Robison L.S., Gannon O.J. (2020). Sex-specific effects of high-fat diet on cognitive impairment in a mouse model of VCID. FASEB J.

[25] Zeng M.D., Fan J.G., Lu L.G. (2008). Guidelines for the diagnosis and treatment of nonalcoholic fatty liver diseases. J Dig Dis.

[26] Sun T., Deng Y., Geng X. (2022). Plasma alkylresorcinol metabolite, a biomarker for whole-grain intake, is inversely associated with risk of nonalcoholic fatty liver disease in a case-control study of Chinese adults. J Nutr.

[27] Inker L.A., Eneanya N.D., Coresh J. (2021). New creatinine- and cystatin C-based equations to estimate GFR without race. N Engl J Med.

[28] Pinto Pereira S.M., Power C. (2013). Life course body mass index, birthweight and lipid levels in mid-adulthood: a nationwide birth cohort study. Eur Heart J.

[29] Cole T.J. (2000). Sympercents: symmetric percentage differences on the 100 log(e) scale simplify the presentation of log transformed data. Stat Med.

[30] Cheng Y., Tian D.Y., Wang Y.J. (2020). Peripheral clearance of brain-derived Aβ in Alzheimer’s disease: pathophysiology and therapeutic perspectives. Transl Neurodegener.

[31] Wiest R., Weiss T.S., Danielyan L., Buechler C. (2021). Serum amyloid beta42 is not eliminated by the cirrhotic liver: a pilot study. J Clin Med.

[32] Cheng Y., He C.Y., Tian D.Y. (2023). Physiological β-amyloid clearance by the liver and its therapeutic potential for Alzheimer’s disease. Acta Neuropathol.

[33] Mackic J.B., Bading J., Ghiso J. (2002). Circulating amyloid-beta peptide crosses the blood-brain barrier in aged monkeys and contributes to Alzheimer’s disease lesions. Vascul Pharmacol.

[34] Kwo P.Y., Cohen S.M., Lim J.K. (2017). ACG clinical guideline: evaluation of abnormal liver chemistries. Am J Gastroenterol.

[35] Jin W.S., Shen L.L., Bu X.L. (2017). Peritoneal dialysis reduces amyloid-beta plasma levels in humans and attenuates Alzheimer-associated phenotypes in an APP/PS1 mouse model. Acta Neuropathol.

[36] Christoforidis M., Schober R., Krohn K. (2005). Genetic-morphologic association study: association between the low density lipoprotein-receptor related protein (LRP) and cerebral amyloid angiopathy. Neuropathol Appl Neurobiol.

[37] Lambert J.C., Wavrant-De Vrièze F., Amouyel P., Chartier-Harlin M.C. (1998). Association at LRP gene locus with sporadic late-onset Alzheimer’s disease. Lancet.

[38] Cao W., Tian S., Zhang H. (2020). Association of low-density lipoprotein receptor-related protein 1 and its rs1799986 polymorphism with mild cognitive impairment in Chinese patients with type 2 diabetes. Front Neurosci.

[39] Kang D.E., Pietrzik C.U., Baum L. (2000). Modulation of amyloid beta-protein clearance and Alzheimer’s disease susceptibility by the LDL receptor-related protein pathway. J Clin Invest.

[40] Sehgal N., Gupta A., Valli R.K. (2012). Withania somnifera reverses Alzheimer’s disease pathology by enhancing low-density lipoprotein receptor-related protein in liver. Proc Natl Acad Sci U S A.

[41] Sáez-Orellana F., Octave J.N., Pierrot N. (2020). Alzheimer’s disease, a lipid story: involvement of peroxisome proliferator-activated receptor α. Cells.

[42] Roy A., Pahan K. (2015). PPARα signaling in the hippocampus: crosstalk between fat and memory. J Neuroimmune Pharmacol.

[43] Gauthier A., Vassiliou G., Benoist F., McPherson R. (2003). Adipocyte low density lipoprotein receptor-related protein gene expression and function is regulated by peroxisome proliferator-activated receptor gamma. J Biol Chem.

[44] Moon J.H., Kim H.J., Yang A.H. (2012). The effect of rosiglitazone on LRP1 expression and amyloid β uptake in human brain microvascular endothelial cells: a possible role of a low-dose thiazolidinedione for dementia treatment. Int J Neuropsychopharmacol.

[45] Luo R., Su L.Y., Li G. (2020). Activation of PPARA-mediated autophagy reduces Alzheimer disease-like pathology and cognitive decline in a murine model. Autophagy.

[46] Ni W., Peng X., Yuan X. (2023). Protocol for Shenzhen Ageing Cohort Study (SZ-ageing): a prospective observational cohort study of elderly disability and cognitive impairment. BMJ Open.

