# Supplementary material for: Peripheral amyloid-β clearance mediates cognitive impairment in non-alcoholic fatty liver disease
Source: eBioMedicine. 2024 Mar 19;102:105079. doi: 10.1016/j.ebiom.2024.105079 (PMC10965463; doi:10.1016/j.ebiom.2024.105079)
Supplement: Supplementary Figs. S1–S8 and Tables S1–S7 [file mmc1.docx]

**Table S1 Estimates with 95% confidence intervals for primary experimental results**

| **Comparison** | **Variable** | **Estimate with 95% CI** | ***P*** | **Corresponding Figure** |
| --- | --- | --- | --- | --- |
| Unpaired *t*-tests | | | | |
| HFD vs. control | Liver weight | 69% (44%, 93%) | 0.00014^a^ | Fig. 1c |
| HFD vs. control | Plasma ALT | 46% (8%, 85) | 0.022 | Fig. 1e |
| HFD vs. control | Plasma AST | 35% (12%, 59) | 0.0062 | Fig. 1f |
| HFD vs. control | Time in target quadrant | -33% (-54%, -12%) | 0.0038 | Fig. 1h |
| HFD vs. control | Distance in target quadrant | -35% (-60%, -10%) | 0.0082 | Fig. 1i |
| HFD vs. control | Swimming speed | 8% (-10%, 26%) | 0.35 | Fig. 1k |
| HFD vs. control | Recognition index | -29% (-42%, -16%) | 0.00013 | Fig. 1l |
| HFD vs. control | Discrimination index | -83% (-119%, -46%) | 0.00013 | Fig. 1m |
| HFD vs. control | Cortical Aβ40 | 23% (1%, 45%) | 0.042 | Fig. 2b |
| HFD vs. control | Cortical Aβ42 | 13% (1%, 25%) | 0.030 | Fig. 2b |
| HFD vs. control | Hippocampal Aβ42 | 56% (25%, 86%) | 0.0015 | Fig. 2c |
| HFD vs. control | Hepatic LRP-1 protein | -39% (-58%, -20%) | 0.0022 | Fig. 3b |
| HFD vs. control | IDE mRNA | -10% (-47%, 27%) | 0.58 | Fig. 3c |
| HFD vs. control | Hepatic LRP-1 mRNA | -43% (-77%, -19%) | 0.0018 | Fig. 3c |
| HFD vs. control | Hepatic LRP-1 | -36% (-55%, -18%) | 0.00075 | Fig. 3d |
| HFD vs. control | Hepatic Aβ40 | -69% (-120%, -17) | 0.014^a^ | Fig. 3e |
| HFD vs. control | Hepatic Aβ42 | -55% (-96%, -15%) | 0.013^a^ | Fig. 3e |
| PA vs. control | LRP-1 protein | -35% (-40%, -31%) | <0.0001 | Fig. 3i |
| PA vs. control | LRP-1 mRNA | -37% (-70%, -3%) | 0.036^a^ | Fig. 3j |
| PA vs. control | Aβ40 uptake | -34% (-41%, -27%) | 0.00018 | Fig. 3k |
| PA vs. control | Aβ42 uptake | -38% (-46%, -30%) | 0.00020 | Fig. 3l |
| HFD vs. control | Hepatic PPARα protein | -58% (-85%, -31%) | 0.0021 | Fig. 4b |
| HFD vs. control | Hepatic PPARγ protein | 68% (39%, 96%) | 0.0011 | Fig. 4b |
| PA vs. control | PPARα protein | -14% (-25%, -2%) | 0.029 | Fig. 4d |
| PA vs. control | PPARγ protein | 30% (21%, 39%) | 0.00084 | Fig. 4d |
| PA +WY14643 vs. PA | LRP-1 protein | 31% (6%, 56%) | 0.027 | Fig. 4f |
| PA +WY14643 vs. PA | LRP-1 mRNA | 37% (12, 63%) | 0.0089 | Fig. 4g |
| PA +WY14643 vs. PA | Aβ40 uptake | 36% (8%, 63%) | 0.022 | Fig. 4h |
| PA +WY14643 vs. PA | Aβ42 uptake | 44% (28%, 60%) | 0.0015 | Fig. 4i |
| Mann-Whitney *U* test | | | | |
| HFD vs. control | Hepatic TG | 116% (59%, 163%) | 0.0019 | Fig. 1d |
| HFD vs. control | Number of crossing | -67% (-67%, 0%) | 0.014 | Fig. 1j |
| HFD vs. control | Hippocampal Aβ40 | 49% (27%, 86%) | 0.00031 | Fig. 2c |
| HFD vs. control | Plasma Aβ40 | 48% (3%, 124%) | 0.049 | Fig. 2d |
| HFD vs. control | IDE protein | 1% (-47%, 9%) | 0.69 | Fig. 3b |
| Linear regression | | | | |
| Not applicable | Total Aβ in cortex and plasma Aβ40 | 0.34 (0.12, 0.56) | 0.0050 | Fig. 2e |
| Not applicable | Total Aβ in hippocampus and plasma Aβ40 | 0.93 (0.36, 1.51) | 0.0035 | Fig. 2f |
| Not applicable | Ln-transformed hepatic Aβ40 and LRP-1 | 0.36 (0.18, 0.53) | 0.00061 | Fig. 3f |
| Not applicable | Ln-transformed hepatic Aβ42 and LRP-1 | 0.20 (0.08, 0.33) | 0.0028 | Fig. 3g |
| Spearman test | | | | |
| Not applicable | Escape latency and hepatic TG | 0.89 (0.70, 0.96) | <0.0001 | Fig. 1n |
| Not applicable | Escape latency and plasma ALT | 0.77 (0.42, 0.92) | 0.00087 | Fig. 1n |
| Not applicable | Escape latency and plasma AST | 0.78 (0.45, 0.92) | 0.00061 | Fig. 1n |
| Not applicable | Time in target quadrant and hepatic TG | -0.70 (-0.89, -0.31) | 0.0031 | Fig. 1n |
| Not applicable | Time in target quadrant and plasma ALT | -0.33 (-0.72, 0.22) | 0.21 | Fig. 1n |
| Not applicable | Time in target quadrant and plasma AST | -0.48 (-0.79, 0.04) | 0.061 | Fig. 1n |
| Not applicable | Distance in target quadrant and hepatic TG | -0.47 (-0.79, 0.05) | 0.066 | Fig. 1n |
| Not applicable | Distance in target quadrant and plasma ALT | -0.12 (-0.59, 0.42) | 0.67 | Fig. 1n |
| Not applicable | Distance in target quadrant and plasma AST | -0.27 (-0.68, 0.28) | 0.32 | Fig. 1n |
| Not applicable | Recognition index and hepatic TG | -0.56 (-0.83, -0.07) | 0.028 | Fig. 1n |
| Not applicable | Recognition index and plasma ALT | -0.60 (-0.85, -0.13) | 0.016 | Fig. 1n |
| Not applicable | Recognition index and plasma AST | -0.75 (-0.91, -0.39) | 0.0012 | Fig. 1n |
| Not applicable | Discrimination index and hepatic TG | -0.55 (-0.83, -0.06) | 0.029 | Fig. 1n |
| Not applicable | Discrimination index and plasma ALT | -0.59 (-0.84, -0.12) | 0.018 | Fig. 1n |
| Not applicable | Discrimination index and plasma AST | -0.75 (-0.91, -0.39) | 0.0012 | Fig. 1n |
| Not applicable | Cortical Aβ40 and hepatic TG | 0.52 (0.02, 0.81) | 0.041 | Fig. 2g |
| Not applicable | Cortical Aβ40 and plasma ALT | 0.46 (-0.07, 0.78) | 0.078 | Fig. 2g |
| Not applicable | Cortical Aβ40 and plasma AST | 0.44 (-0.09, 0.77) | 0.089 | Fig. 2g |
| Not applicable | Cortical Aβ42 and hepatic TG | 0.60 (0.13, 0.85) | 0.017 | Fig. 2g |
| Not applicable | Cortical Aβ42 and plasma ALT | 0.77 (0.43, 0.92) | 0.00076 | Fig. 2g |
| Not applicable | Cortical Aβ42 and hepatic AST | 0.62 (0.17, 0.86) | 0.011 | Fig. 2g |
| Not applicable | Total Aβ in cortex and hepatic TG | 0.62 (0.16, 0.86) | 0.013 | Fig. 2g |
| Not applicable | Total Aβ in cortex and plasma ALT | 0.79 (0.47, 0.93) | 0.00048 | Fig. 2g |
| Not applicable | Total Aβ in cortex and hepatic AST | 0.63 (0.19, 0.86) | 0.0098 | Fig. 2g |
| Not applicable | Hippocampal Aβ40 and hepatic TG | 0.75 (0.40, 0.91) | 0.0011 | Fig. 2g |
| Not applicable | Hippocampal Aβ40 and plasma ALT | 0.59 (0.11, 0.84) | 0.019 | Fig. 2g |
| Not applicable | Hippocampal Aβ40 and hepatic AST | 0.58 (0.11, 0.84) | 0.019 | Fig. 2g |
| Not applicable | Hippocampal Aβ42 and hepatic TG | 0.66 (0.22, 0.87) | 0.0071 | Fig. 2g |
| Not applicable | Hippocampal Aβ42 and plasma ALT | 0.58 (0.11, 0.84) | 0.020 | Fig. 2g |
| Not applicable | Hippocampal Aβ42 and hepatic AST | 0.66 (0.23, 0.87) | 0.0068 | Fig. 2g |
| Not applicable | Total Aβ in hippocampus and hepatic TG | 0.66 (0.22, 0.87) | 0.0071 | Fig. 2g |
| Not applicable | Total Aβ in hippocampus and plasma ALT | 0.58 (0.11, 0.84) | 0.020 | Fig. 2g |
| Not applicable | Total Aβ in hippocampus and hepatic AST | 0.66 (0.23, 0.87) | 0.0068 | Fig. 2g |
| Not applicable | Plasma Aβ40 and hepatic TG | 0.79 (0.58, 0.93) | 0.00041 | Fig. 2g |
| Not applicable | Plasma Aβ40 and plasma ALT | 0.80 (0.50, 0.93) | 0.00032 | Fig. 2g |
| Not applicable | Plasma Aβ40 and hepatic AST | 0.64 (0.20, 0.87) | 0.0089 | Fig. 2g |

Estimates were mean% differences for unpaired *t*-tests, median% differences for Mann-Whitney *U* test, regression coefficients for linear regression, and Spearman correlation coefficients for Spearman test.

^a^ *P* value was calculated by unpaired *t*-tests with Welch’s correction.

**Table S2 Spearman correlation coefficients with 95% confidence intervals for correlations between cerebral Aβ levels and cognitive performance**

| **Variable** | **Escape latency** | | **Time in target quadrant** | | **Distance in target quadrant** | | **Recognition index** | | **Discrimination index** | |
| --- | --- | --- | --- | --- | --- | --- | --- | --- | --- | --- |
|  | ***r* (95% CI)** | ***P*** | ***r* (95% CI)** | ***P*** | ***r* (95% CI)** | ***P*** | ***r* (95% CI)** | ***P*** | ***r* (95% CI)** | ***P*** |
| Cortical Aβ40 | 0.52 (0.01, 0.81) | 0.044 | -0.54 (-0.82, -0.04) | 0.034 | -0.47 (-0.79, 0.05) | 0.068 | -0.24 (-0.67, 0.31) | 0.37 | -0.24 (-0.67, 0.30) | 0.36 |
| Cortical Aβ42 | 0.66 (0.22, 0.87) | 0.0071 | -0.42 (-0.76, 0.11) | 0.11 | -0.25 (-0.67, 0.30) | 0.35 | -0.66 (-0.87, -0.23) | 0.0068 | -0.66 (-0.88, -0.23) | 0.0065 |
| Total Aβ in cortex | 0.67 (0.25, 0.88) | 0.0056 | -0.43 (-0.77, 0.10) | 0.10 | -0.25 (-0.67, 0.29) | 0.34 | -0.64 (-0.87, -0.19) | 0.0093 | -0.64 (-0.87, -0.20) | 0.0089 |
| Hippocampal Aβ40 | 0.86 (0.62, 0.95) | <0.0001 | -0.62 (-0.86, -0.16) | 0.012 | -0.40 (-0.75, 0.14) | 0.13 | -0.62 (-0.86, -0.16) | 0.013 | -0.61 (-0.86, -0.15) | 0.013 |
| Hippocampal Aβ42 | 0.69 (0.29, 0.89) | 0.0038 | -0.59 (-0.84, -0.11) | 0.019 | -0.42 (-0.77, 0.11) | 0.11 | -0.55 (-0.83, -0.05) | 0.031 | -0.54 (-0.82, -0.04) | 0.033 |
| Total Aβ in Hippocampus | 0.69 (0.29, 0.89) | 0.0038 | -0.59 (-0.84, -0.11) | 0.019 | -0.42 (-0.77, 0.11) | 0.11 | -0.55 (-0.83, -0.05) | 0.031 | -0.54 (-0.82, -0.04) | 0.033 |

**Table S3 Demographic and clinical characteristics of the NAFLD cases according to the severity of NAFLD**

| **Characteristics** | **Severity of NAFLD** | | ***P*** |
| --- | --- | --- | --- |
|  | **Moderate or severe (*n* = 64)** | **Mild (*n* = 485)** |  |
| Age (years) | 61 (48.5, 66) | 59 (45, 64) | 0.067 |
| Sex | | | |
| Male, *n* (%) | 47 (73.4) | 344 (71.0) | 0.68 |
| Female, *n* (%) | 17 (26.6) | 141 (29.0) |  |
| BMI (kg/m^2^) | 27.12 (3.13) | 25.7 (2.63) | 0.0011^a^ |
| Current smoker, *n* (%) | 20 (31.3) | 166 (34.2) | 0.64 |
| Current drinker, *n* (%) | 22 (34.4) | 115 (23.7) | 0.064 |
| Physical activity, *n* (%) | 29 (45.3) | 200 (41.2) | 0.53 |
| Diabetes, *n* (%) | 9 (14.1) | 42 (8.7) | 0.16 |
| Hyperlipidaemia, *n* (%) | 38 (59.4) | 237 (48.9) | 0.11 |
| Hypertension, *n* (%) | 25 (39.1) | 138 (28.5) | 0.081 |
| CVD, *n* (%) | 7 (10.9) | 30 (6.2) | 0.25^b^ |
| TG (mmol/L) | 1.93 (1.51–2.72) | 1.63 (1.20–2.35) | 0.0074 |
| Total cholesterol (mmol/L) | 5.12 (4.32–5.82) | 4.98 (4.34–5.66) | 0.82 |
| LDL cholesterol (mmol/L) | 2.61 (1.90–3.22) | 2.59 (1.80–3.27) | 0.70 |
| HDL cholesterol (mmol/L) | 1.29 (1.04–1.42) | 1.29 (1.16–1.47) | 0.25 |
| ALT (U/L) | 28.5 (22–37) | 24 (18–31) | 0.00091 |
| Creatinine (μmol/L) | 77.7 (66.3–89.1) | 71.3 (61.4–83.0) | 0.041 |
| eGFR (mL/min/1.73 m^2^) | 95.08 (79.39–104.41) | 96.89 (86.82–110.97) | 0.064 |
| Aβ40 (ng/L) | 148.55 (132.71–176.18) | 138.82 (120.76–161.18) | 0.0025 |
| Aβ42 (ng/L) | 15.46 (13.32–18.10) | 13.70 (10.47–17.22) | 0.0032 |

Data were presented as mean (SD) for normally distributed data, median (interquartile range) for non-normally distributed data, or *n* (%) for categorical data.

^a^ *P* value was calculated by unpaired *t*-tests with Welch’s correction.

^b^ *P* value was calculated by χ^2^ test with correction for continuity.

**Table S4 Demographic and clinical characteristics of the NAFLD cases according to the history of diagnosed NAFLD**

| **Characteristics** | **History of diagnosed NAFLD** | | ***P*** |
| --- | --- | --- | --- |
|  | **Yes (*n* = 179)** | **No (*n* = 370)** |  |
| Age (years) | 56 (43, 64) | 60 (47, 64) | 0.025 |
| Sex | | | |
| Male, *n* (%) | 140 (78.2) | 251 (67.8) | 0.012 |
| Female, *n* (%) | 39 (21.8) | 119 (32.2) |  |
| BMI (kg/m^2^) | 26.26 (2.53) | 25.72 (2.80) | 0.028 |
| Current smoker, *n* (%) | 73 (40.8) | 113 (30.5) | 0.018 |
| Current drinker, *n* (%) | 50 (27.9) | 87 (23.5) | 0.26 |
| Physical activity, *n* (%) | 69 (38.6) | 160 (43.2) | 0.30 |
| Diabetes, *n* (%) | 18 (10.1) | 33 (8.9) | 0.67 |
| Hyperlipidaemia, *n* (%) | 110 (61.5) | 165 (44.6) | 0.00022 |
| Hypertension, *n* (%) | 57 (31.8) | 106 (28.7) | 0.44 |
| CVD, *n* (%) | 19 (10.6) | 18 (4.9) | 0.012 |
| TG (mmol/L) | 1.70 (1.26–2.42) | 1.64 (1.22–2.39) | 0.39 |
| Total cholesterol (mmol/L) | 4.98 (4.35–5.66) | 5.02 (4.32–5.70) | 0.60 |
| LDL cholesterol (mmol/L) | 2.50 (1.70–3.27) | 2.62 (1.87–3.25) | 0.26 |
| HDL cholesterol (mmol/L) | 1.28 (1.15–1.44) | 1.31 (1.15–1.47) | 0.56 |
| ALT (U/L) | 27 (19–35) | 23 (19–30) | 0.013 |
| Creatinine (μmol/L) | 74.0 (62.3–85.0) | 72.3 (61.4–83.0) | 0.49 |
| eGFR (mL/min/1.73 m^2^) | 98.39 (88.22–111.78) | 96.17 (84.36–107.69) | 0.068 |
| Aβ40 (ng/L) | 140.69 (125.04–161.18) | 138.78 (120.76–164.88) | 0.54 |
| Aβ42 (ng/L) | 13.79 (10.73–18.08) | 14.00 (10.66–17.39) | 0.42 |

Data were presented as mean (SD) for normally distributed data, median (interquartile range) for non-normally distributed data, or *n* (%) for categorical data.

**Table S5 Spearman correlation coefficients with 95% confidence intervals for correlations between hepatic LRP-1 and Aβ levels with cerebral and plasma Aβ levels as well as cognitive performance**

| **Variable** | **Hepatic LRP-1** | | **Hepatic Aβ40** | | **Hepatic Aβ42** | | **Total Aβ in liver** | |
| --- | --- | --- | --- | --- | --- | --- | --- | --- |
|  | ***r* (95% CI)** | ***P*** | ***r* (95% CI)** | ***P*** | ***r* (95% CI)** | ***P*** | ***r* (95% CI)** | ***P*** |
| Escape latency | -0.84 (-0.95, -0.58) | <0.0001 | -0.75 (-0.91, -0.39) | 0.0012 | -0.79 (-0.93, -0.47) | 0.00048 | -0.75 (-0.91, -0.40) | 0.0012 |
| Time in target quadrant | 0.56 (0.07, 0.83) | 0.026 | 0.50 (-0.02, 0.80) | 0.052 | 0.50 (-0.01, 0.80) | 0.051 | 0.49 (-0.02, 0.80) | 0.054 |
| Distance in target quadrant | 0.23 (-0.32, 0.66) | 0.40 | 0.21 (-0.33, 0.65) | 0.43 | 0.26 (-0.29, 0.68) | 0.33 | 0.25 (-0.30, 0.67) | 0.35 |
| Recognition index | 0.66 (0.22, 0.87) | 0.0071 | 0.59 (0.11, 0.84) | 0.019 | 0.57 (0.08, 0.83) | 0.024 | 0.54 (0.05, 0.82) | 0.032 |
| Discrimination index | 0.65 (0.21, 0.87) | 0.0078 | 0.59 (0.11, 0.84) | 0.019 | 0.57 (0.08, 0.83) | 0.024 | 0.54 (0.05, 0.82) | 0.032 |
| Cortical Aβ40 | -0.47 (-0.79, 0.05) | 0.070 | -0.30 (-0.70, 0.24) | 0.25 | -0.32 (-0.71, 0.23) | 0.23 | -0.32 (-0.71, 0.22) | 0.23 |
| Cortical Aβ42 | -0.75 (-0.91, -0.40) | 0.0012 | -0.71 (-0.89, -0.31) | 0.0030 | -0.70 (-0.89, -0.29) | 0.0036 | -0.69 (-0.89, -0.28) | 0.0042 |
| Total Aβ in cortex | -0.77 (-0.92, -0.43) | 0.00076 | -0.71 (-0.90, -0.32) | 0.0027 | -0.71 (-0.89, -0.31) | 0.0029 | -0.70 (-0.89, -0.30) | 0.0034 |
| Hippocampal Aβ40 | -0.77 (-0.92, -0.43) | 0.00081 | -0.82 (-0.94, -0.54) | 0.00019 | -0.82 (-0.94, -0.54) | 0.00017 | -0.83 (-0.94, -0.55) | 0.00015 |
| Hippocampal Aβ42 | -0.66 (-0.87, -0.23) | 0.0068 | -0.56 (-0.83, -0.07) | 0.027 | -0.60 (-0.85, -0.14) | 0.015 | -0.56 (-0.83, -0.08) | 0.025 |
| Total Aβ in Hippocampus | -0.66 (-0.87, -0.23) | 0.0068 | -0.56 (-0.83, -0.07) | 0.027 | -0.60 (-0.85, -0.14) | 0.015 | -0.56 (-0.83, -0.08) | 0.025 |
| Plasma Aβ40 | -0.63 (-0.86, -0.18) | 0.011 | -0.61 (-0.85, -0.14) | 0.015 | -0.66 (-0.87, -0.23) | 0.0068 | -0.63 (-0.86, -0.17) | 0.011 |

**Table S6 Mean % (95% confidence interval) difference in plasma Aβ40 and Aβ42 levels associated with rs1799986 genotypes**

| **Variates** | ***n* (%)** | **Aβ40** | | | **Aβ42** | | |
| --- | --- | --- | --- | --- | --- | --- | --- |
|  |  | **Model 1** | **Model 2** | **Model 3** | **Model 1** | **Model 2** | **Model 3** |
| Alleles | | | | | | | |
| C | 2028 (92.3) | 0.00 (ref.) | 0.00 (ref.) | 0.00 (ref.) | 0.00 (ref.) | 0.00 (ref.) | 0.00 (ref.) |
| T | 168 (7.7) | -8.41 (-11.51, -5.30) | -8.00 (-11.10, -4.90) | -7.98 (-11.06, -4.90) | -12.19 (-17.13, -7.24) | -11.68 (-16.65, -6.72) | -11.48 (-16.43, -6.52) |
| *P* |  | <0.0001 | <0.0001 | <0.0001 | <0.0001 | <0.0001 | <0.0001 |
| Genotypes | | | | | | | |
| CC | 935 (85.2) | 0.00 (ref.) | 0.00 (ref.) | 0.00 (ref.) | 0.00 (ref.) | 0.00 (ref.) | 0.00 (ref.) |
| CT + TT | 208 (14.8) | -9.27 (-12.52, -6.03) | -8.89 (-12.13, -5.65) | -8.87 (-12.09, -5.66) | -12.81 (-17.99, -7.63) | -12.30 (-17.50, -7.11) | -12.12 (-17.31, -6.94) |
| *P* |  | <0.0001 | <0.0001 | <0.0001 | <0.0001 | <0.0001 | <0.0001 |

Model 1 was adjusted for age and sex. Model 2 was additionally adjusted for BMI, current smoking status, current drinking status, physical activity, triglyceride, total cholesterol, and eGFR. Model 3 was additionally adjusted for history of diabetes, history of hypertension, history of CVD.

**Table S7 Mean % (95% confidence interval) difference in plasma Aβ40 and Aβ42 levels associated with rs1799986 genotypes according to NAFLD status**

| **Genotypes** | **NAFLD** | | **Regression coefficients (95% CI) for interaction term** | ***P* for interaction** |
| --- | --- | --- | --- | --- |
|  | **No** | **Yes** |  |  |
| Aβ40 | | | | |
| CC | 0.00 (ref.) | 0.00 (ref.) | -10.05 (-16.29, -3.80) | 0.0017 |
| TC+TT | -3.95 (-7.75, -0.14) | -14.67 (-19.77, -9.57) |  |  |
| *P* | 0.042 | <0.0001 |  |  |
| Aβ42 | | | | |
| CC | 0.00 (ref.) | 0.00 (ref.) | -16.46 (-26.59, -6.33) | 0.0015 |
| TC+TT | -4.49 (-10.68, 1.70) | -21.06 (-29.36, -12.76) |  |  |
| *P* | 0.16 | <0.0001 |  |  |

Multivariable analysis was adjusted for age, sex, BMI, current smoking status, current drinking status, physical activity, history of diabetes, history of hypertension, history of CVD, TG, total cholesterol, and eGFR.


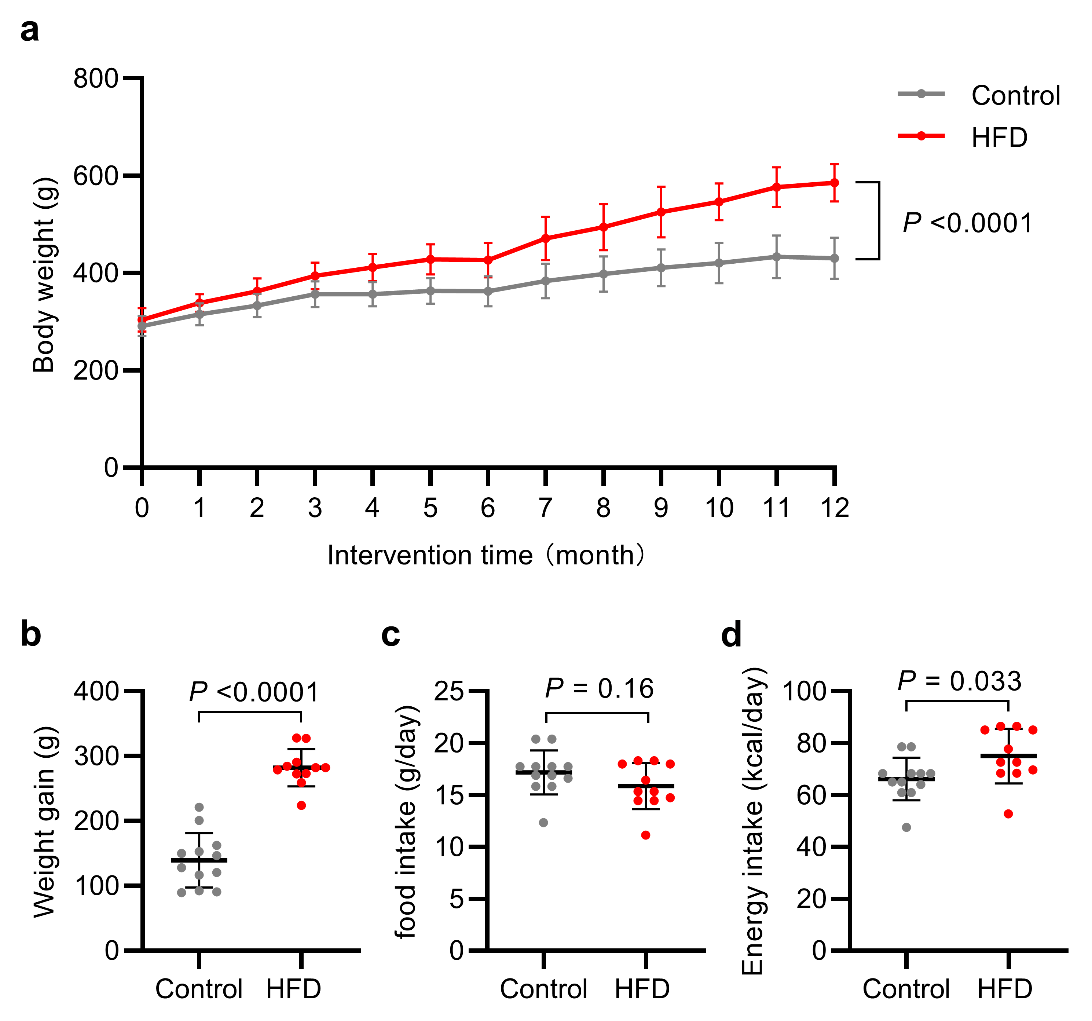


**Fig. S1 Body weight changes as well as food and energy intake in rats.** (a) Trajectories of body weight changes in control and HFD-fed rats during the 12-month feeding period (*n* = 11-12 per group). (b-d) Weight gain (b), food intake (c), and energy intake (d) of control and HFD-fed rats (*n* = 11-12 per group). Data were presented as mean ± SD. *P*-values were calculated by two-way ANOVA (a) or unpaired Student’s *t*-test (b, c, and d).


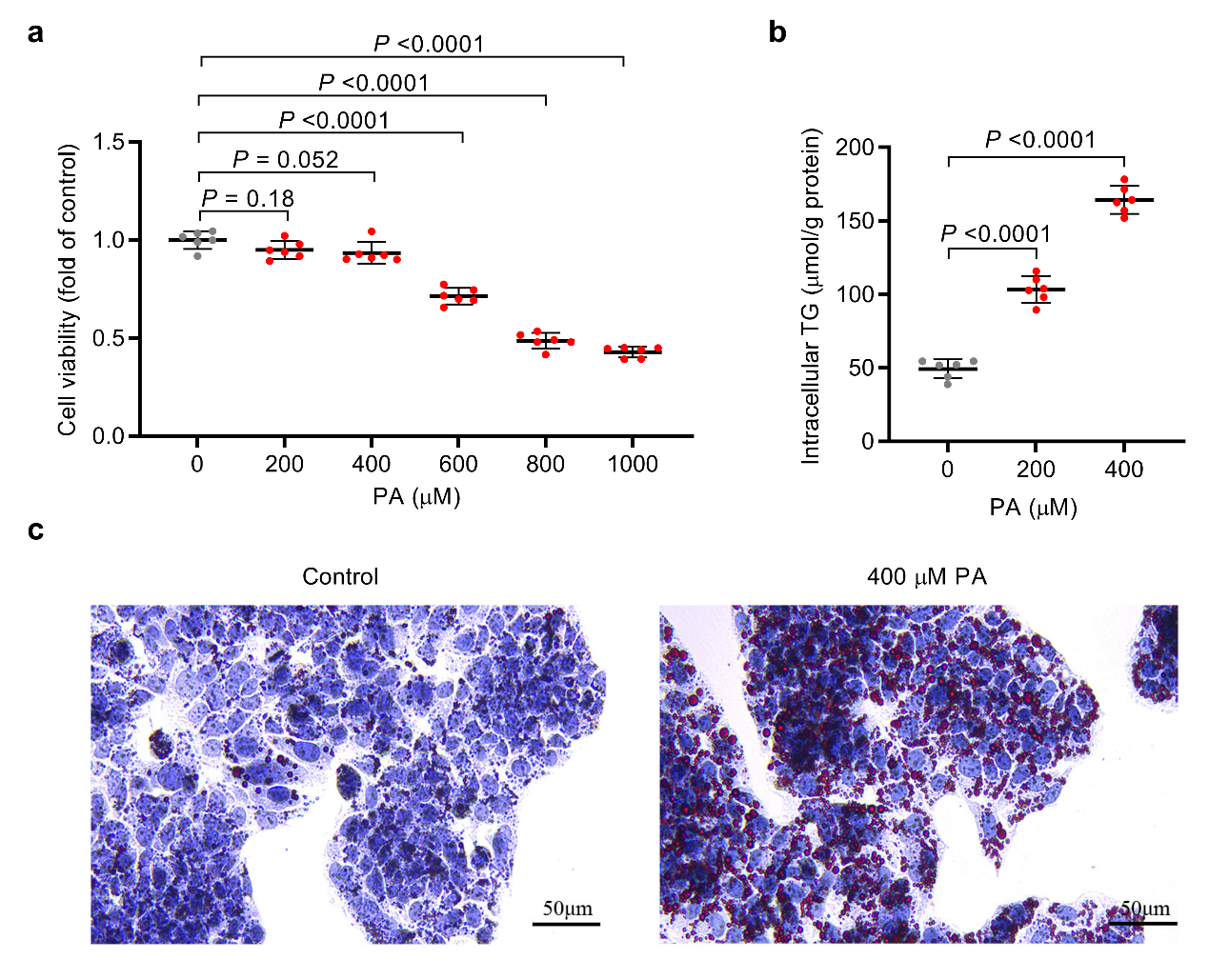


**Fig. S2 Effects of PA on the viability and intracellular TG in HepG2 cells.** (a, b) Viability (a) and intracellular TG (b) of HepG2 cells treated with indicated concentrations of PA for 24 h (*n* = 6 per group). (c) Oil Red O staining for HepG2 cells treated with or without 400 μM PA for 24 h (*n* = 3 per group); scale bars indicate 50 μm. Data were presented as mean ± SD. *P*-values were calculated by one-way ANOVA with Dunnett’s multiple comparisons test.


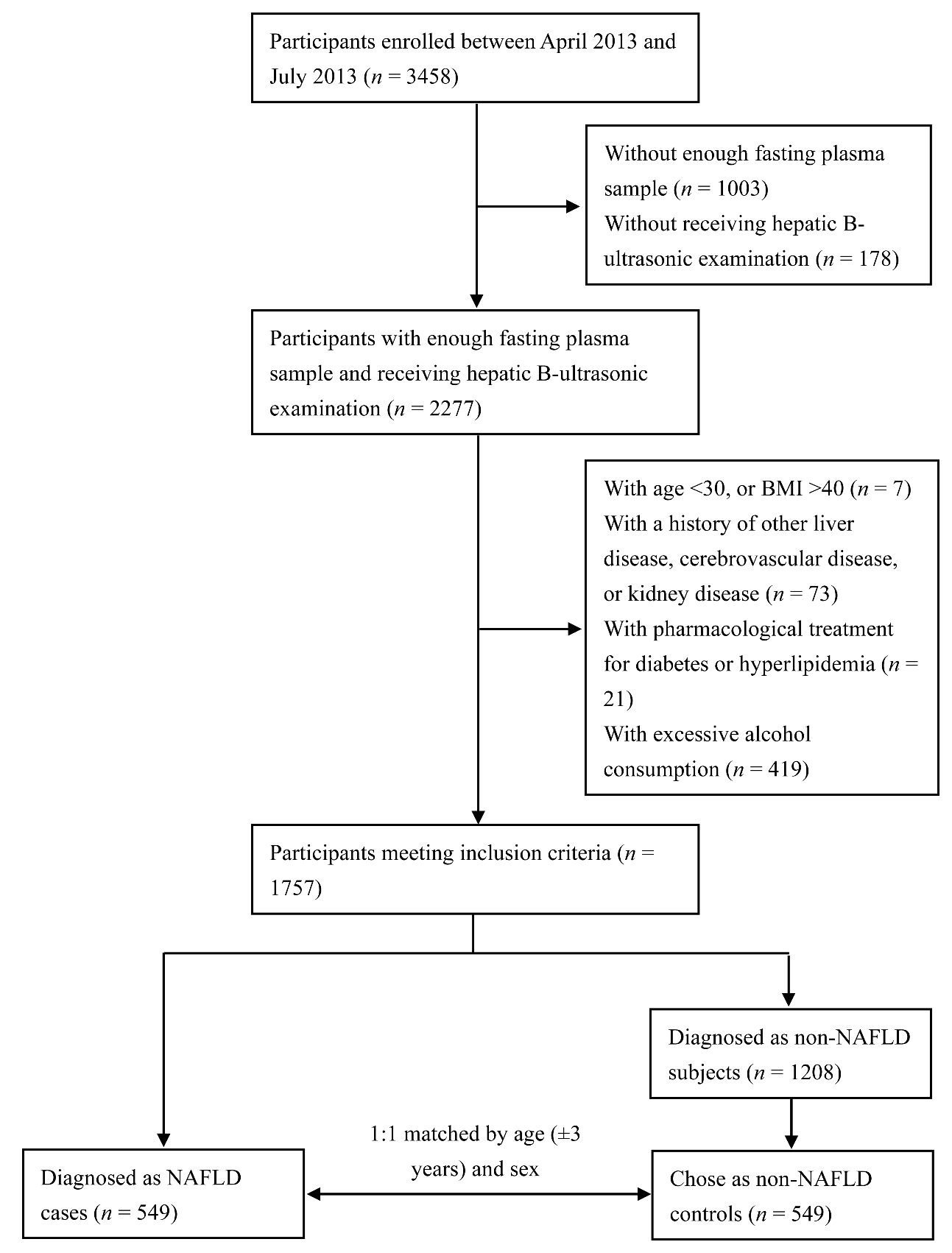


**Fig. S3 Flowcharts of the participant recruitment and case-control selection.**

**
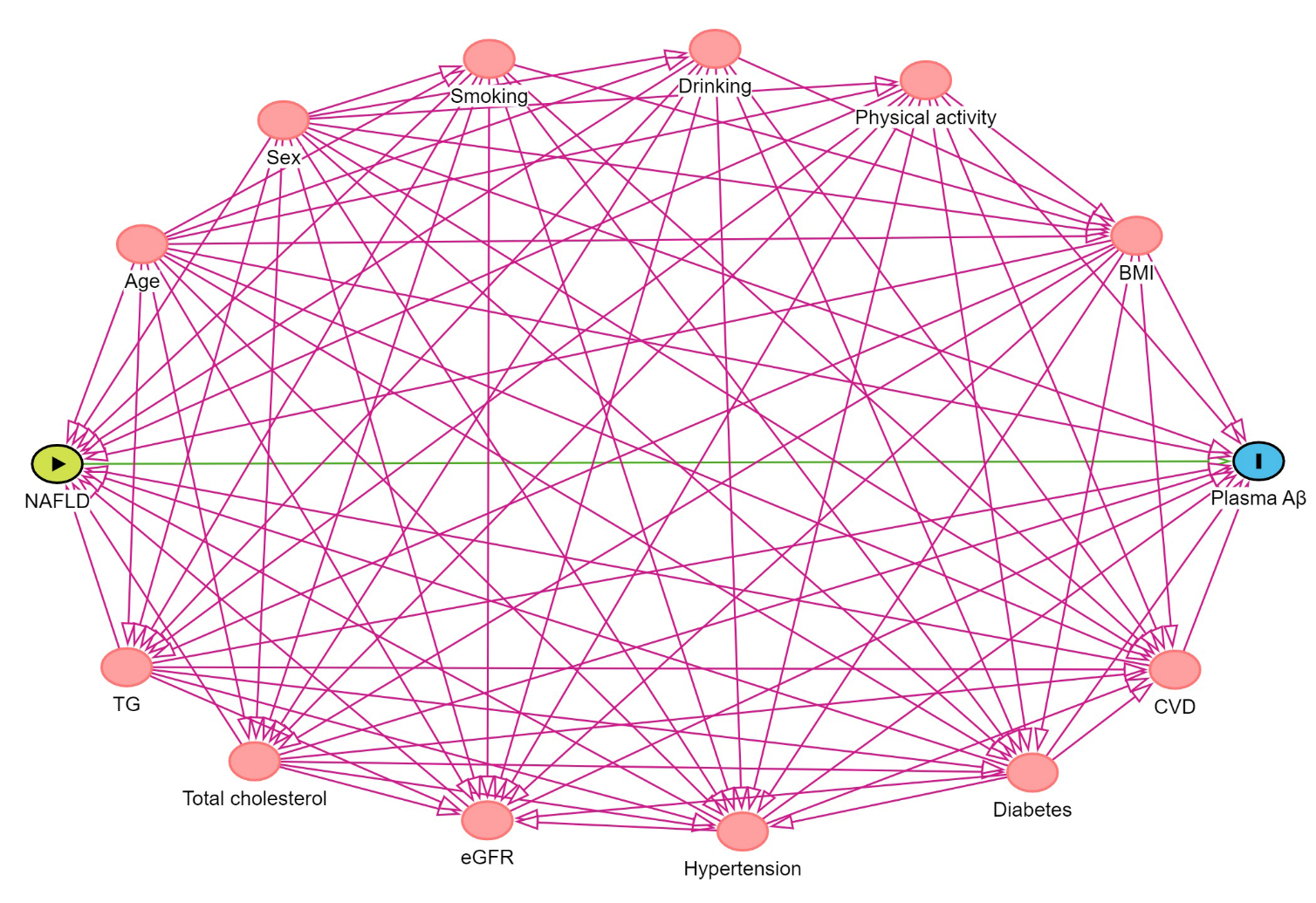
**

**Fig. S4 Causal directed acyclic graph for the association between NAFLD and plasma Aβ.** Green circle represents exposure, blue circle represents outcome, and red circles represent ancestors of the exposure and outcome. Green line represents causal path, and red lines represent biasing paths.


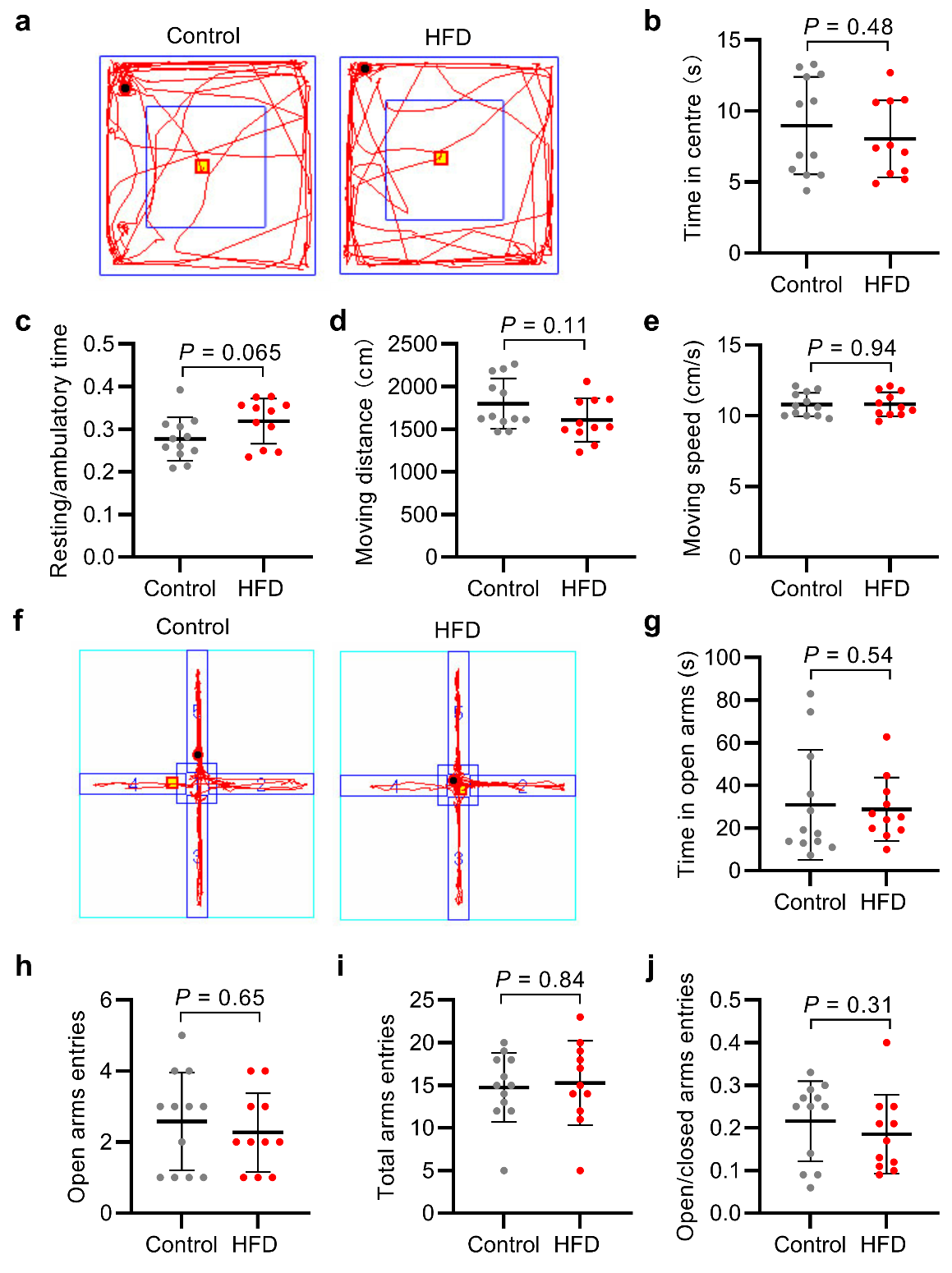


**Fig. S5 HFD did not alter spontaneous locomotor and anxiety-like behaviour in rats.** (a-e) Representative motion track (a), time spent in the centre area (b), the ratio of resting to ambulatory time (c), moving distance (d), and moving speed (e) in the open field test of control and HFD-fed rats (*n* = 11-12 per group). (f-j) Representative motion track (f), time spent in open arms (g), number of times entered open arms (h), number of times entered total arms (i), and the ratio of times entered open arms to closed arms (j) in the elevated plus-maze test of control and HFD-fed rats (*n* = 11-12 per group). Data were presented as mean ± SD. *P*-values were calculated by unpaired Student’s *t*-test (b-e) or Mann-Whitney *U* test (g-j).


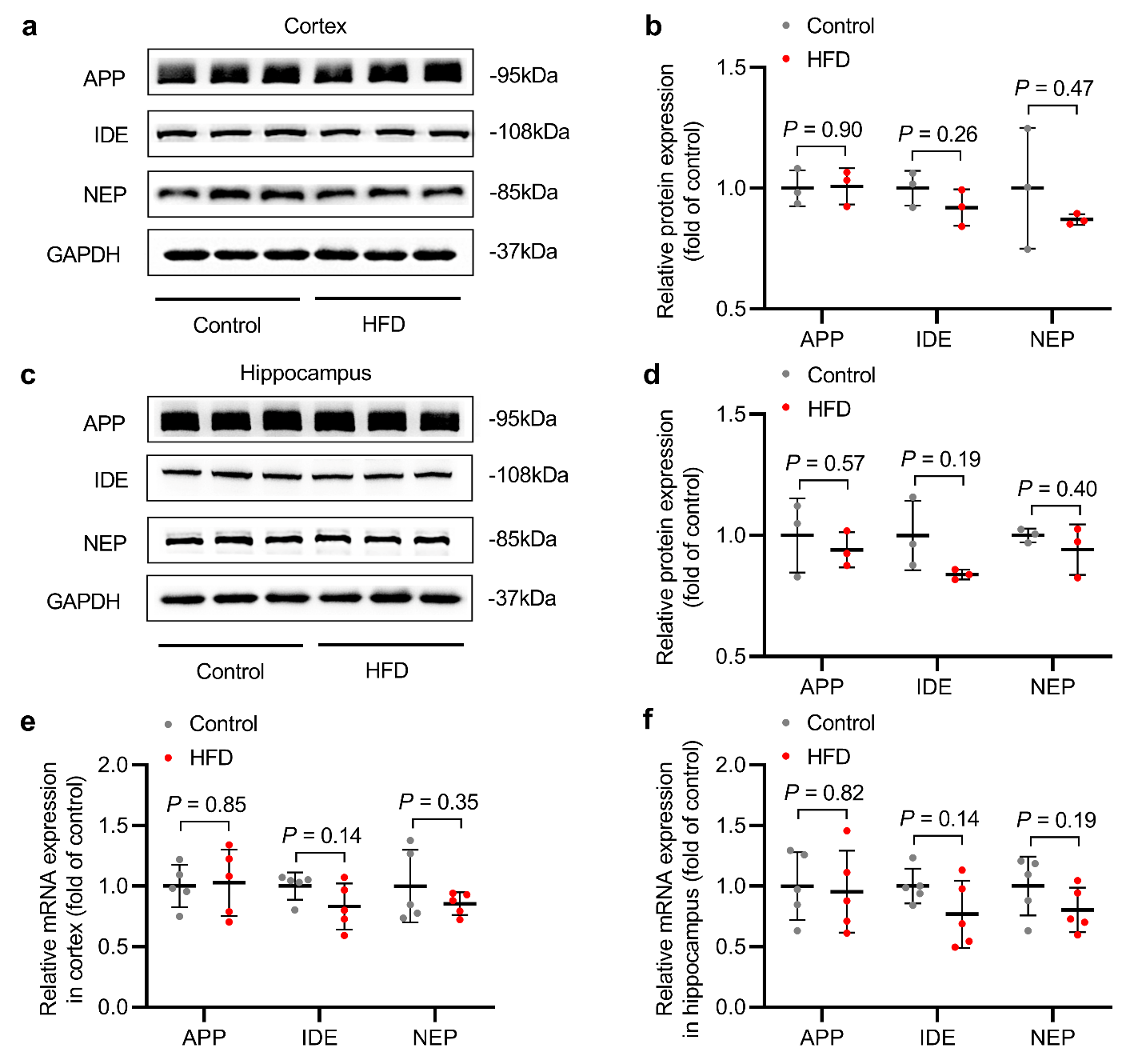


**Fig. S6 HFD did not alter the expression of APP, IDE, and NEP in the cortex and hippocampus of rats.** (a, b) Western blot bands (a) and quantification (b) for APP, IDE, and NEP protein expression in the cortex of control and HFD-fed rats (*n* = 3 per group). (c, d) Western blot bands (c) and quantification (d) of APP, IDE, and NEP protein expression in the hippocampus of control and HFD-fed rats (*n* = 3 per group). (e, f) Real-time PCR quantification of mRNA expression of APP, IDE, and NEP in the cortex (e) and hippocampus (f) of control and HFD-fed rats (*n* = 5 per group). Data were presented as mean ± SD. *P*-values were calculated by unpaired Student’s *t*-test or Welch’s *t*-test (NEP in panels b and e, IDE in panel d) except for IDE mRNA in the cortex (e), which was compared with the Mann-Whitney *U* test.


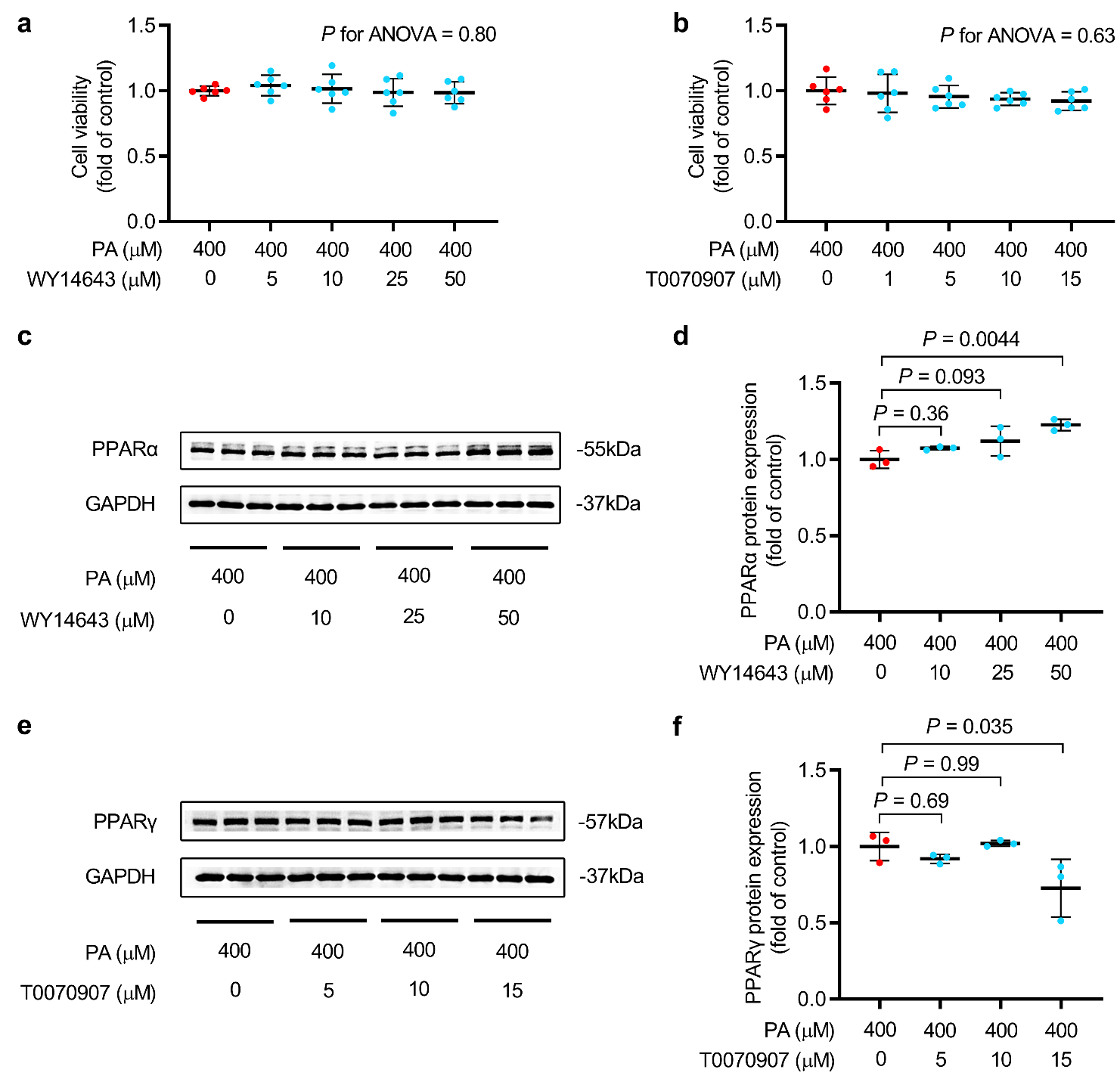


**Fig. S7 Effects of WY14643 and T0070907 on the viability and PPARs protein expression in PA-treated HepG2 cells.** (a, b) Viability of HepG2 cells treated with 400 μM PA and indicated concentrations of WY14643 (a) or T0070907 (b) for 24 h (*n* = 6 per group). (c, d) Western blot bands (c) and quantification (d) of PPARα protein expression in HepG2 cells treated with 400 μM PA and indicated concentrations of WY14643 (*n* = 3 per group). (e, f) Western blot bands (e) and quantification (f) of PPARγ protein expression in HepG2 cells treated with 400 μM PA and indicated concentrations of T007090 (*n* = 3 per group). Data were presented as mean ± SD. *P*-values were calculated by one-way ANOVA with Dunnett’s multiple comparisons test.


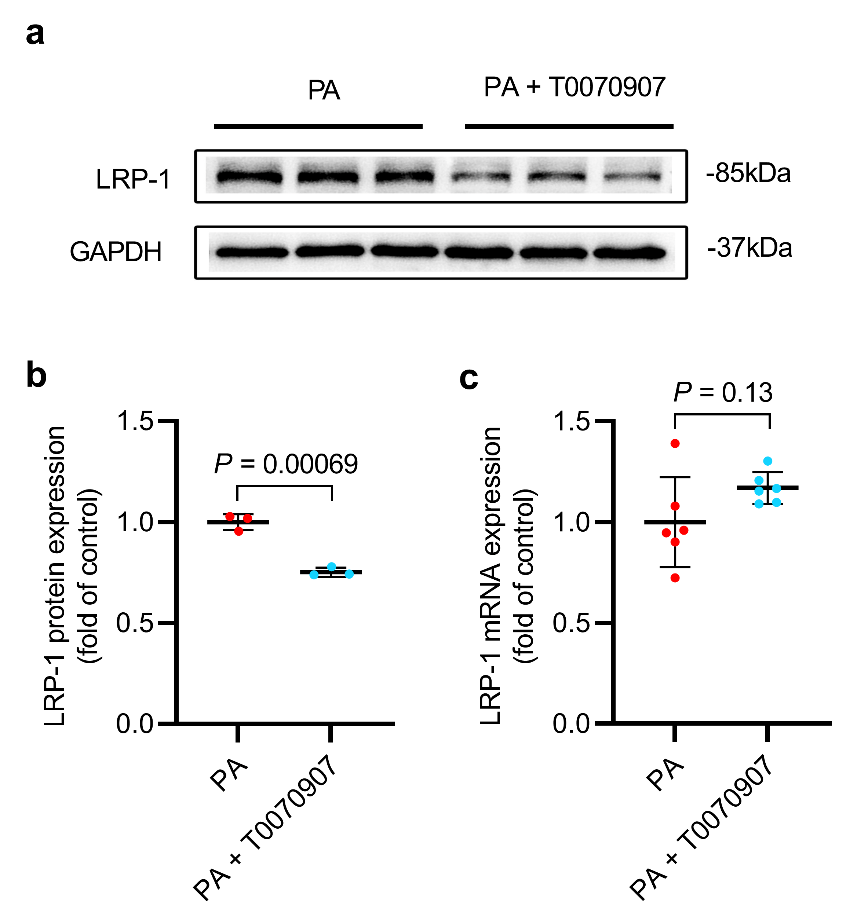


**Fig. S8 Effects of T0070907 on LRP-1 expression in PA-treated HepG2 cells.** (a, b) Western blot bands (a) and quantification (b) for LRP-1 protein expression in HepG2 cells treated with 400 μM PA and with or without 15 μM T0070907 (*n* = 3 per group). (c) Real-time PCR quantification for LRP-1 mRNA expression in HepG2 cells treated with 400 μM PA and with or without 15 μM T0070907 (*n* = 6 per group). Data were presented as mean ± SD. *P*-values were calculated by unpaired Student’s *t*-test (b) or Welch’s *t*-test (c).
