# Supplementary figures and images for: Peripheral amyloid-β clearance mediates cognitive impairment in non-alcoholic fatty liver disease
Source: eBioMedicine. 2024 Mar 19;102:105079. doi: 10.1016/j.ebiom.2024.105079 (PMC10965463; doi:10.1016/j.ebiom.2024.105079)

Fig. 3a

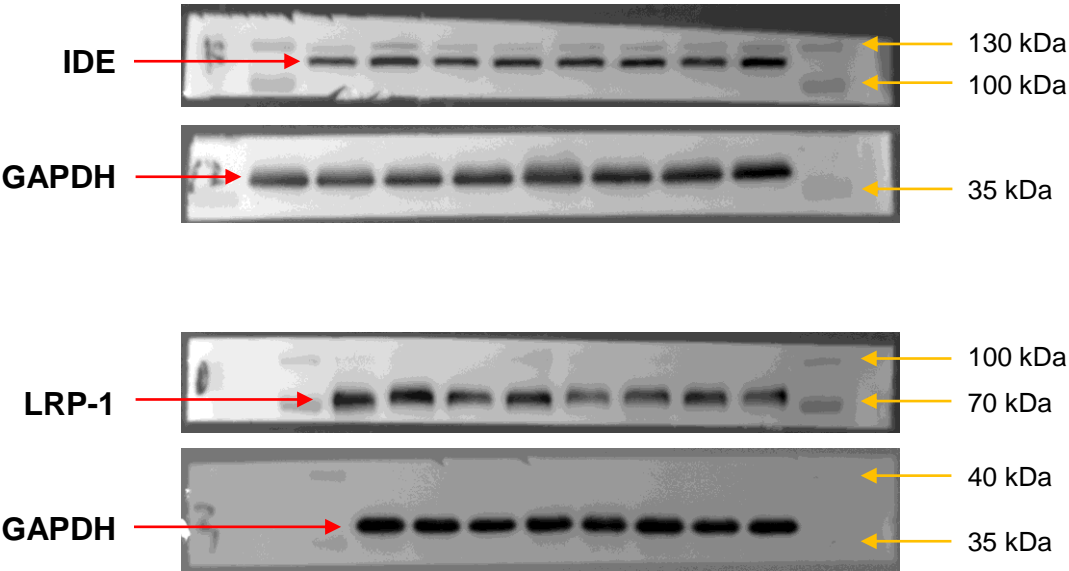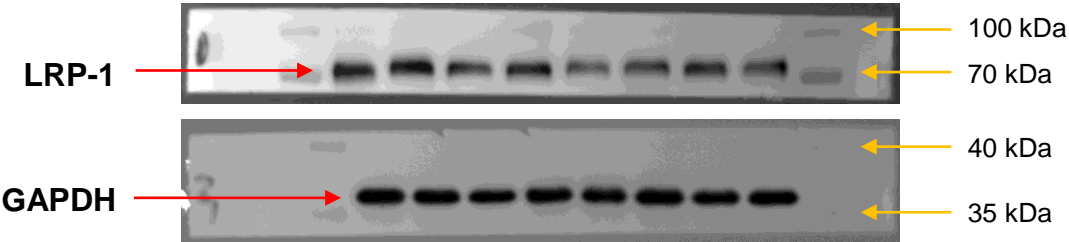

Fig. 3h

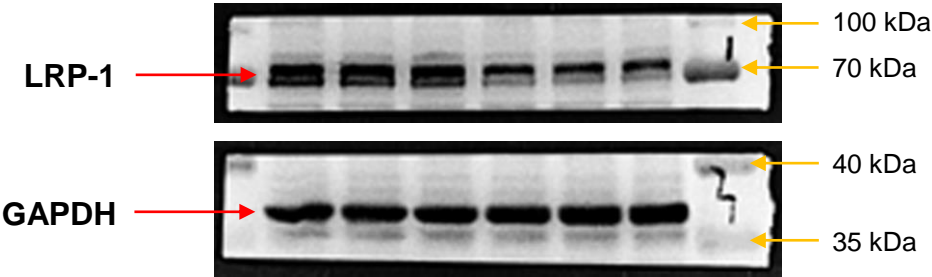

Fig. 4a

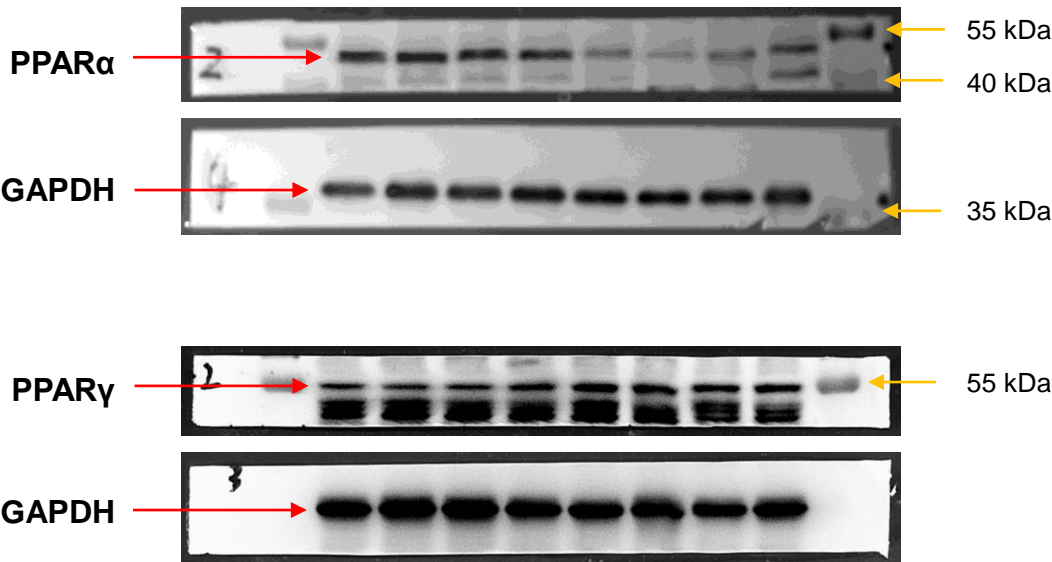

Fig. 4c

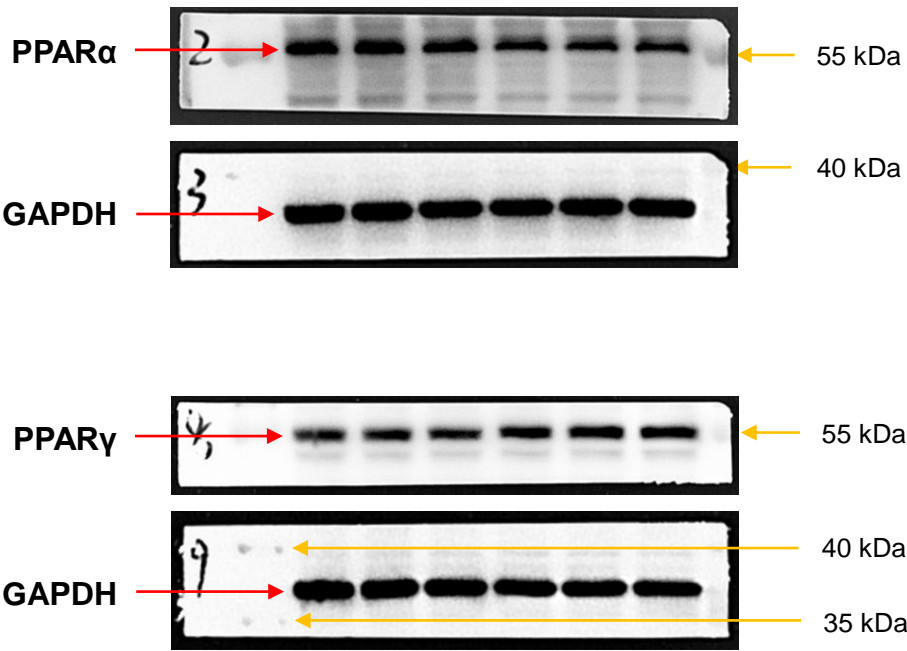

Fig. 4e

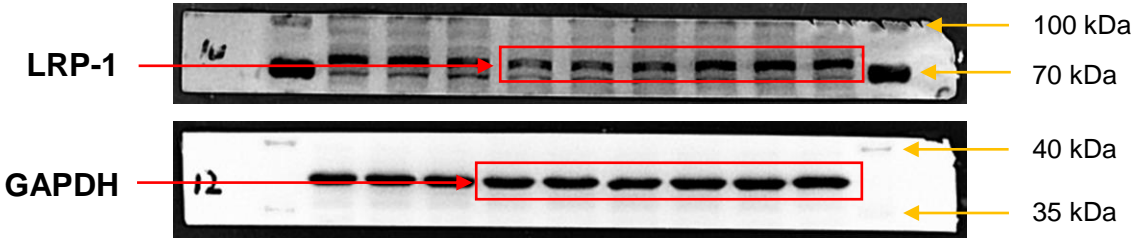

Fig. S6a

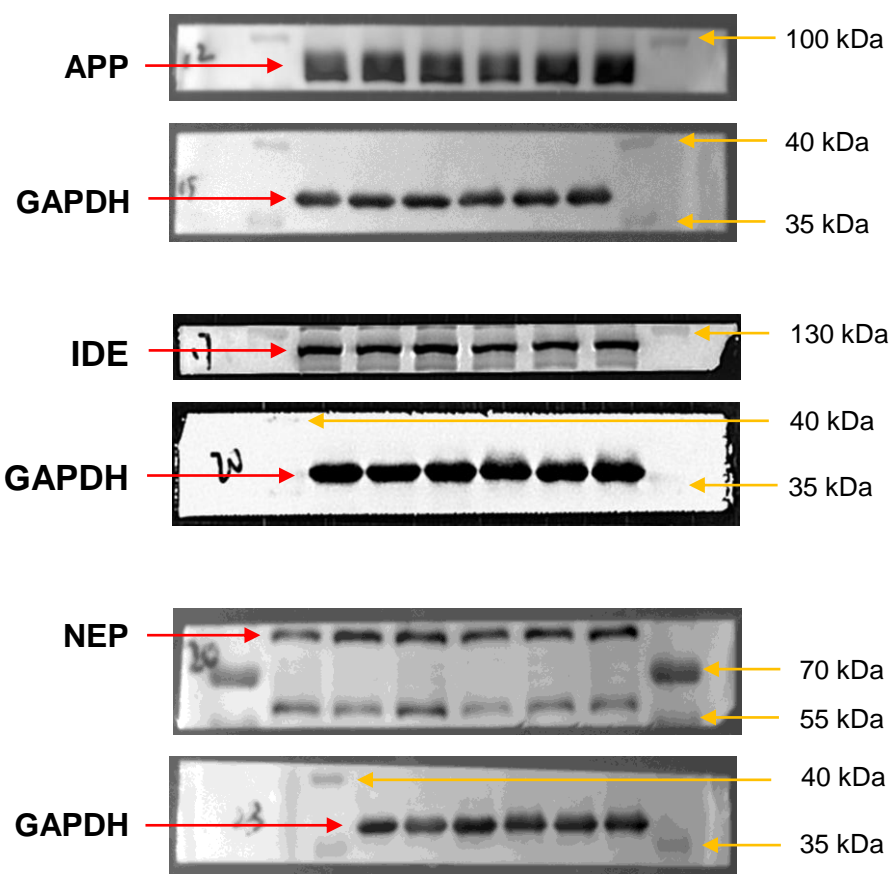

Fig. S6c

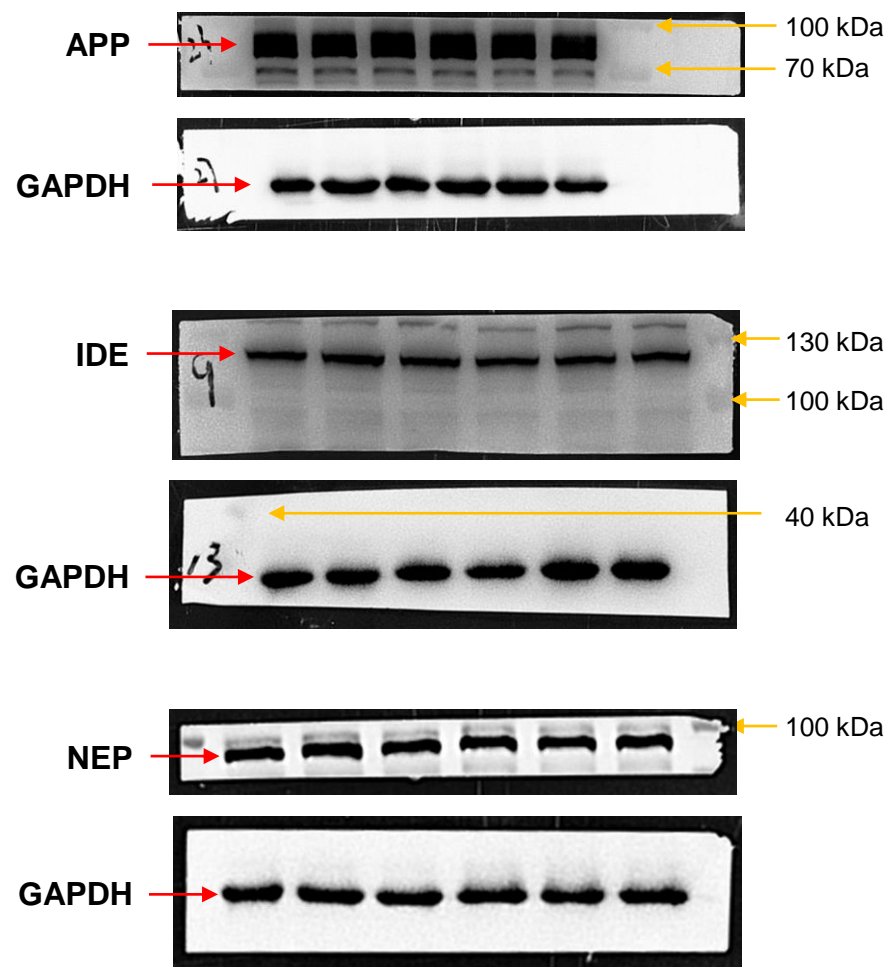

Fig. S7c

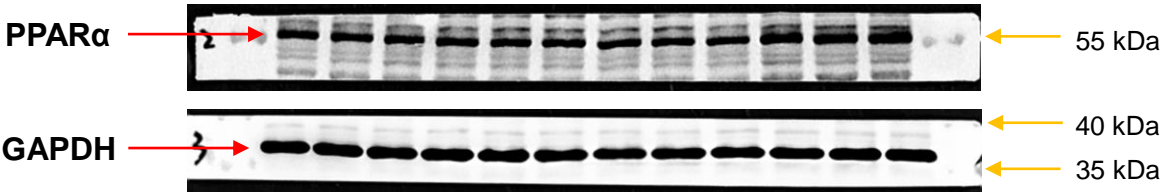

Fig. S7e

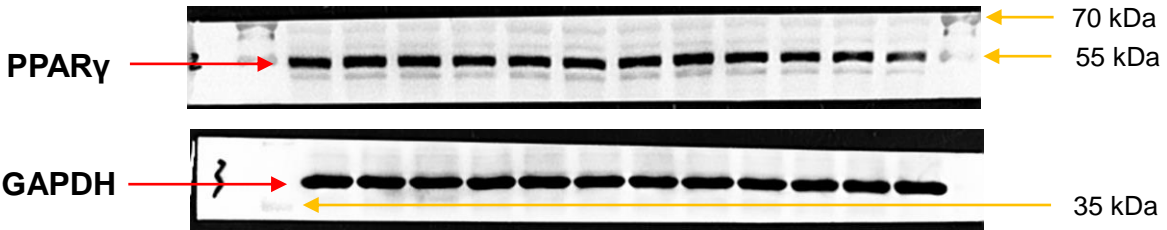

Fig. S8a

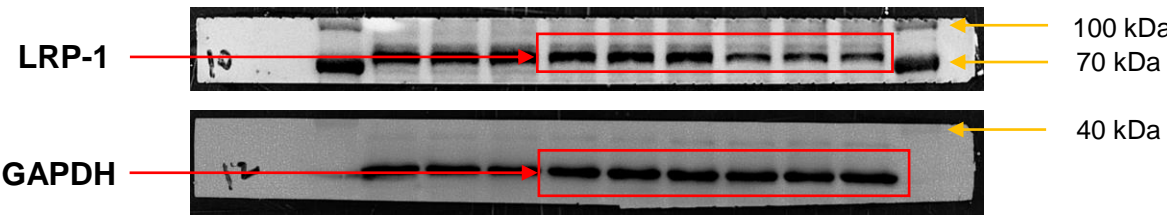

Supplement: Supplemental Western blots [file mmc2.pdf]
