# Supplementary material for: Peripheral amyloid-β clearance mediates cognitive impairment in non-alcoholic fatty liver disease
Source: eBioMedicine. 2024 Mar 19;102:105079. doi: 10.1016/j.ebiom.2024.105079 (PMC10965463; doi:10.1016/j.ebiom.2024.105079)
Supplement: Reagent Validation [file mmc3.pdf]

Antibody Validation

Company and location	Antibody	Catalog #	RRID
Proteintech, Rosemont, IL, USA	APP [1]	25524-1-AP	AB_2880118
Abcam, Cambridge, UK	IDE [2]	ab32216	AB_775686
	NEP [3]	ab256494	AB_2894853
	PPARα [4]	ab227074	AB_3083737
	PPARγ [5]	ab209350	AB_2890099
Cell Signaling Technology, Danvers, MA, USA	LRP-1 [6]	64099S	AB_2799654
	GAPDH [7]	2118	AB_561053
	Rabbit Secondary antibody [8]	7074	AB_2099233
BioLegend, San Diego, CA, USA	Aβ [9]	803001	AB_2564653
Thermo Fisher Scientific, Waltham, MA, USA	Mouse Secondary antibody [10]	31800	AB_228305

References

1. Ai J, Wang H, Chu P, et al. The neuroprotective effects of phosphocreatine on Amyloid Beta 25-35-induced differentiated neuronal cell death through inhibition of AKT /GSK-3β /Tau/APP /CDK5 pathways in vivo and vitro. *Free Radic Biol Med* 2021; 162: 181-90.

2. Wang T, Chen Y, Zou Y, et al. Locomotor hyperactivity in the early-stage Alzheimer's disease-like pathology of APP/PS1 mice: Associated with impaired polarization of astrocyte aquaporin 4. *Aging Dis* 2022; 13(5): 1504-22.

3. Chen Q, Wu Y, Yu Y, Wei J, Huang W. Rho-kinase inhibitor hydroxyfasudil protects against HIV-1 Tat-induced dysfunction of tight junction and neprilysin/Aβ transfer receptor expression in mouse brain microvessels. *Mol Cell Biochem* 2021; 476(5): 2159-70.

4. Mishra S, Sadagopan N, Dunkerly-Eyring B, et al. Inhibition of phosphodiesterase type 9 reduces obesity and cardiometabolic syndrome in mice. *J Clin Invest* 2021; 131(21).

5. Costa AM, Russo F, Senn L, Ibatici D, Cannazza G, Biagini G. Antiseizure effects of cannabidiol leading to increased peroxisome proliferator-activated receptor gamma levels in the hippocampal CA3 subfield of epileptic rats. *Pharmaceuticals (Basel)* 2022; 15(5): 495.

6. Garcia J, Chang R, Steinberg RA, et al. Modulation of hepatic amyloid precursor protein and lipoprotein receptor-related protein 1 by chronic alcohol intake: Potential link between liver steatosis and amyloid-β. *Front Physiol* 2022; 13: 930402.

7. Chen YF, Song Q, Colucci P, et al. Basolateral amygdala activation enhances object recognition memory by inhibiting anterior insular cortex activity. *Proc Natl Acad Sci U S A* 2022; 119(22): e2203680119.

8. Kay Y, Tsan L, Davis EA, et al. Schizophrenia-associated SAP97 mutations increase glutamatergic synapse strength in the dentate gyrus and impair contextual

episodic memory in rats. *Nat Commun* 2022; 13(1): 798.

9. Thakker DR, Weatherspoon MR, Harrison J, et al. Intracerebroventricular amyloid-beta antibodies reduce cerebral amyloid angiopathy and associated micro-hemorrhages in aged Tg2576 mice. *Proc Natl Acad Sci U S A* 2009; 106(11): 4501-6.

10. Sabogal-Guáqueta AM, Osorio E, Cardona-Gómez GP. Linalool reverses neuropathological and behavioral impairments in old triple transgenic Alzheimer's mice. *Neuropharmacology* 2016; 102: 111-20.

Cell Line Validation

The human hepatoma cell line HepG2 was acquired from the American Type Culture Collection (Rockville, MD, USA) and validated by STR profiling. The documentation for STR profiling was provided below.

For Research Use Only

APP/Beta Amyloid Polyclonal antibody

proteintech®
Antibodies | ELISA kits | Proteins
www.ptglab.com

Catalog Number:25524-1-AP Featured Product 55 Publications

Basic Information

Catalog Number:
25524-1-AP

Size:
150ul , Concentration: 550 μg/ml by Nanodrop;

Source:
Rabbit

Isotype:
IgG

Immunogen Catalog Number:
AG22408

GenBank Accession Number:
BC065529

GeneID (NCBI):
351

Full Name:
amyloid beta (A4) precursor protein

Observed MW:
100 kDa

Purification Method:
Antigen affinity purification

Recommended Dilutions:
WB 1:500-1:2000
IHC 1:50-1:500

Applications

Tested Applications:
IHC, WB, ELISA

Cited Applications:
IF, IHC, WB

Species Specificity:
human, mouse, rat

Cited Species:
human, rat, mouse

Note-IHC: suggested antigen retrieval with TE buffer pH 9.0; (*) Alternatively, antigen retrieval may be performed with citrate buffer pH 6.0

Positive Controls:

WB : SH-SY5Y cells, HAP1, mouse brain tissue, HeLa cells, rat brain tissue, C6 cells

IHC : human gliomas tissue, human brain tissue

Background Information

Aβ derives from APP via proteolytic cleavage by proteases called α-, β- and γ-secretase. The α-secretase cleavage precludes the formation of Aβ, while the β- and γ-cleavages generate APP components with amyloidogenic features. Amyloid beta A4 precursor protein(APP), encoded by APP gene which locate on human chromosome 21q, is a cell surface receptor and performs physiological functions on the surface of neurons relevant to neurite growth, neuronal adhesion and axonogenesis. APP expressed in all fetal tissues and is pronounced in brain, kidney, heart and spleen, but weak in liver. Defects in APP are the cause of Alzheimer disease type 1 (AD1). Amyloid β (Aβ) precursor protein (APP) is a 100-140 kDa transmembrane glycoprotein that exists as several isoforms. This antibody can recognize several isoforms of both mature and immature amyloid beta (A4) precursor protein, including APP770, APP677, APP695, APP696, APP733, APP751, APP752, and APP639. APP can be cleaved into several chains, this antibody could recognize fragments C99, Amyloid-beta protein 42, Amyloid-beta protein 40, C83, P3(40), C80, Gamma-secretase C-terminal fragment 59, Gamma-secretase C-terminal fragment 57, Gamma-secretase C-terminal fragment 50, C31.

Notable Publications

Author	Pubmed ID	Journal	Application
Shenya Xu	33183805	Sci Total Environ	WB, IHC
Zhongkang Zhu	34695452	Neurosci Lett	IHC,IF
Jie Ai	33131696	Free Radic Biol Med	WB

Storage

Storage:
Store at -20°C. Stable for one year after shipment.
Storage Buffer:
PBS with 0.02% sodium azide and 50% glycerol pH 7.3.
Aliquoting is unnecessary for -20°C storage

***** 20ul sizes contain 0.1% BSA**

For technical support and original validation data for this product please contact:
T: 1 (888) 4PTGLAB (1-888-478-4522) (toll free in USA), or 1(312) 455-8498 (outside USA)
E: proteintech@ptglab.com
W: ptglab.com

This product is exclusively available under Proteintech Group brand and is not available to purchase from any other manufacturer.

Selected Validation Data

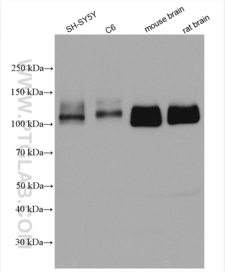

Various lysates were subjected to SDS PAGE followed by western blot with 25524-1-AP (APP antibody) at dilution of 1:1000 incubated at room temperature for 1.5 hours.

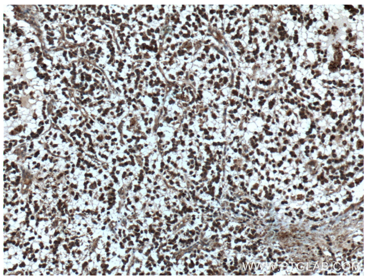

Immunohistochemical analysis of paraffin-embedded human gliomas tissue slide using 25524-1-AP (APP, C-Terminal antibody at dilution of 1:200 (under 10x lens). Heat mediated antigen retrieval with Tris-EDTA buffer (pH 9.0).

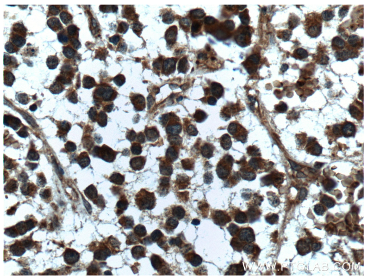

Immunohistochemical analysis of paraffin-embedded human gliomas tissue slide using 25524-1-AP (APP, C-Terminal antibody at dilution of 1:200 (under 40x lens). Heat mediated antigen retrieval with Tris-EDTA buffer (pH 9.0).

abcam

Product datasheet

Anti-Insulin degrading enzyme / IDE antibody ab32216

KO VALIDATED

★★★★☆ 9 Abreviews 70 References 3 Images

Overview

**Product name**	Anti-Insulin degrading enzyme / IDE antibody
**Description**	Rabbit polyclonal to Insulin degrading enzyme / IDE
**Host species**	Rabbit
**Specificity**	Replenishment batches of our polyclonal antibody, ab32216 are tested in WB. Previous batches were additionally validated in IHC-FoFr. This application is still expected to work and is covered by our Abpromise guarantee. You may also be interested in our alternative recombinant antibody, **ab133561**.
**Tested applications**	**Suitable for:** WB, IHC-FoFr
**Species reactivity**	**Reacts with:** Mouse, Rat, Human<br>**Predicted to work with:** Cow, Cat, Dog
**Immunogen**	Synthetic peptide corresponding to Human Insulin degrading enzyme/ IDE aa 950 to the C-terminus.<br>(Peptide available as **ab32215**)
**General notes**	The Life Science industry has been in the grips of a reproducibility crisis for a number of years. Abcam is leading the way in addressing this with our range of recombinant monoclonal antibodies and knockout edited cell lines for gold-standard validation. Please check that this product meets your needs before purchasing.<br>If you have any questions, special requirements or concerns, please send us an inquiry and/or contact our Support team ahead of purchase. Recommended alternatives for this product can be found below, along with publications, customer reviews and Q&As

Properties

**Form**	Liquid
**Storage instructions**	Shipped at 4°C. Store at +4°C short term (1-2 weeks). Upon delivery aliquot. Store at -20°C or -80°C. Avoid freeze / thaw cycle.
**Storage buffer**	pH: 7.40<br>Preservative: 0.02% Sodium azide<br>Constituent: PBS

Batches of this product that have a concentration < 1mg/ml may have BSA added as a stabilising agent. If you would like information about the formulation of a specific lot, please contact our scientific support team who will be happy to help.

Purity Immunogen affinity purified

Clonality Polyclonal

Isotype IgG

Applications

The Abpromise guarantee Our **Abpromise guarantee** covers the use of ab32216 in the following tested applications.

The application notes include recommended starting dilutions; optimal dilutions/concentrations should be determined by the end user.

Application	Abreviews	Notes
**WB**	★★★☆☆ (2)	Use a concentration of 1 µg/ml. Detects a band of approximately 118 kDa (predicted molecular weight: 118 kDa).Can be blocked with **Human Insulin degrading enzyme / IDE peptide (ab32215)**.
**IHC-FoFr**	★★★★★ (2)	1/100.

Target

Function Plays a role in the cellular breakdown of insulin, IAPP, glucagon, bradykinin, kallidin and other peptides, and thereby plays a role in intercellular peptide signaling. Degrades amyloid formed by APP and IAPP. May play a role in the degradation and clearance of naturally secreted amyloid beta-protein by neurons and microglia.

Sequence similarities Belongs to the peptidase M16 family.

Post-translational modifications The N-terminus is blocked.

Cellular localization Cytoplasm. Cell surface. Present at the cell surface of neuron cells. The membrane-associated isoform is approximately 5 kDa larger than the known cytosolic isoform.

Images

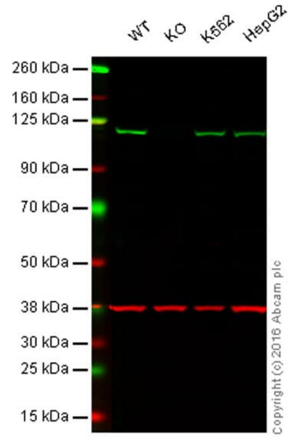

Western blot - Anti-Insulin degrading enzyme / IDE antibody (ab32216)

Lane 1: Wild-type HAP1 cell lysate (20 µg)

Lane 2: Insulin degrading enzyme / IDE knockout HAP1 cell lysate (20 µg)

Lane 3: K562 cell lysate (20 µg)

Lane 4: HepG2 cell lysate (20 µg)

Lanes 1 - 4: Merged signal (red and green). Green - ab32216 observed at 120 kDa. Red - loading control, **ab8245**, observed at 37 kDa.

ab32216 was shown to specifically react with Insulin degrading enzyme / IDE in wild-type HAP1 cells. No band was observed when Insulin degrading enzyme / IDE knockout samples were examined. Wild-type and Insulin degrading enzyme / IDE knockout samples were subjected to SDS-PAGE. ab32216 and **ab8245** (loading control to GAPDH) were diluted at 1µg/ml and 1/10,000 respectively and incubated overnight at 4°C. Blots were developed with Goat anti-Rabbit IgG H&L (IRDye® 800CW) preadsorbed (**ab216773**) and Goat anti-Mouse IgG H&L (IRDye® 680RD) preadsorbed (**ab216776**) secondary antibodies at 1/10,000 dilution for 1 hour at room temperature before imaging.

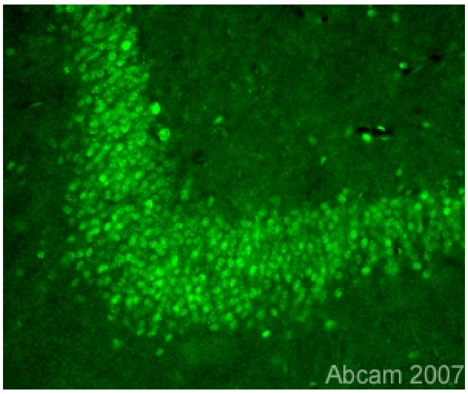

Immunohistochemistry (PFA perfusion fixed frozen sections) - Anti-Insulin degrading enzyme / IDE antibody (ab32216)

This image is courtesy of Sophie Pezet, CNRS, Paris, France

Immunofluorescent staining for Insulin degrading enzyme/IDE in rat brain rat hippocampus using Rabbit polyclonal to Insulin degrading enzyme/IDE (ab32216). . The staining is located in the neuronal soma and is finely punctuated. The picture was acquired using the X20 objective. Protocol details: Rats were intracardially perfused with 4% paraformaldehyde. Whole brain tissue was post-fixed overnight in the same fixative, and cryoprotected in 20% sucrose and frozen in OCT. 30µm coronal sections were cut by cryostat for use in fre floating IHC. Primary antibody ab32216 was incubated overnight at 1/100 at room temperature. Secondary antibody Alexa fluor 488 1/1000 was incubated for 2 hours at room temperature.

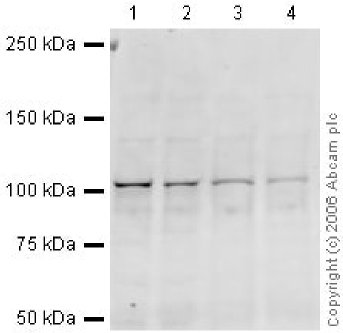

Western blot - Anti-Insulin degrading enzyme / IDE antibody (ab32216)

All lanes : Anti-Insulin degrading enzyme / IDE antibody (ab32216) at 1 µg/ml

Lane 1 : Mouse Brain at 20 µg/ml
Lane 2 : Brain (Rat) Whole Cell Lysate - normal tissue at 20 µg
Lane 3 : Mouse Hippocampus Lysate at 20 µg
Lane 4 : Rat Hippocampus Lysate at 20 µg

Secondary
All lanes : Goat polyclonal to Rabbit IgG (Alexa Fluor® 680) at 1/10000 dilution

Performed under reducing conditions.

Predicted band size: 118 kDa
Observed band size: 118 kDa

Please note: All products are "FOR RESEARCH USE ONLY. NOT FOR USE IN DIAGNOSTIC PROCEDURES"

Our Abpromise to you: Quality guaranteed and expert technical support

- Replacement or refund for products not performing as stated on the datasheet
- Valid for 12 months from date of delivery
- Response to your inquiry within 24 hours

- We provide support in Chinese, English, French, German, Japanese and Spanish
- Extensive multi-media technical resources to help you
- We investigate all quality concerns to ensure our products perform to the highest standards

If the product does not perform as described on this datasheet, we will offer a refund or replacement. For full details of the Abpromise, please visit **https://www.abcam.com/abpromise** or contact our technical team.

Terms and conditions

- Guarantee only valid for products bought direct from Abcam or one of our authorized distributors

abcam

Product datasheet

Anti-CD10 antibody [EPR22867-118] ab256494

KO VALIDATED Recombinant RabMAb

★★★★★ 5 Abreviews 8 References 14 Images

Overview

**Product name**	Anti-CD10 antibody [EPR22867-118]
**Description**	Rabbit monoclonal [EPR22867-118] to CD10
**Host species**	Rabbit
**Tested applications**	**Suitable for:** ICC/IF, WB, IHC-P, IP, IHC-Fr<br>**Unsuitable for:** Flow Cyt
**Species reactivity**	**Reacts with:** Mouse, Rat, Human
**Immunogen**	Recombinant fragment. This information is proprietary to Abcam and/or its suppliers.
**Positive control**	WB: Wild type HAP1, Raji and Ramos whole cell lysate, Rat lung, Rat kidney, Human tonsil and Mouse lung lysates. IHC-P: Human kidney, Human placenta, Human diffuse large B-cell lymphoma, Mouse kidney and Rat kidney tissues. IHC-Fr: Mouse and rat kidney tissue. ICC/IF: Ramos, WEHI-231 and 2.4G2 cells. IP: Raji cells.
**General notes**	This product is a recombinant monoclonal antibody, which offers several advantages including:<br>- High batch-to-batch consistency and reproducibility<br>- Improved sensitivity and specificity<br>- Long-term security of supply<br>- Animal-free production<br>For more information **see here**.<br>Our RabMAb® technology is a patented hybridoma-based technology for making rabbit monoclonal antibodies. For details on our patents, please refer to **RabMAb® patents**.

Properties

**Form**	Liquid
**Storage instructions**	Shipped at 4°C. Store at +4°C short term (1-2 weeks). Upon delivery aliquot. Store at -20°C long term. Avoid freeze / thaw cycle.
**Storage buffer**	pH: 7.2<br>Preservative: 0.01% Sodium azide<br>Constituents: PBS, 0.5% BSA, 40% Glycerol (glycerin, glycerine)
**Purity**	Protein A purified
**Clonality**	Monoclonal

Clone number EPR22867-118

Isotype IgG

Applications

The Abpromise guarantee Our **Abpromise guarantee** covers the use of ab256494 in the following tested applications.

The application notes include recommended starting dilutions; optimal dilutions/concentrations should be determined by the end user.

Application	Abreviews	Notes
ICC/IF	★★★★☆ (1)	1/500.
WB	★★★★★ (1)	1/1000. Predicted molecular weight: 85 kDa.
IHC-P	★★★★★ (1)	1/500.
IP		1/30.
IHC-Fr	★★★★★ (1)	1/100.

Application notes Is unsuitable for Flow Cyt.

Target

Function Thermolysin-like specificity, but is almost confined on acting on polypeptides of up to 30 amino acids. Biologically important in the destruction of opioid peptides such as Met- and Leu-enkephalins by cleavage of a Gly-Phe bond. Able to cleave angiotensin-1, angiotensin-2 and angiotensin 1-9. Involved in the degradation of atrial natriuretic factor (ANF). Displays UV-inducible elastase activity toward skin preelastic and elastic fibers.

Sequence similarities Belongs to the peptidase M13 family.

Cellular localization Cell membrane.

Images

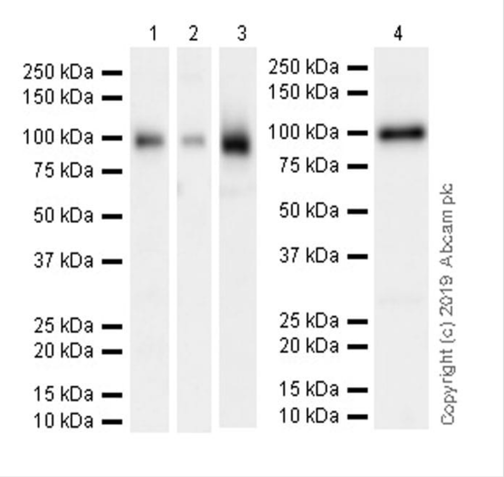

Western blot - Anti-CD10 antibody [EPR22867-118] (ab256494)

All lanes : Anti-CD10 antibody [EPR22867-118] (ab256494) at 1/1000 dilution

Lane 1 : Rat lung tissue lysate at 10 µg
Lane 2 : Rat kidney tissue lysate at 10 µg
Lane 3 : Human tonsil tissue lysate at 10 µg
Lane 4 : Mouse lung tissue lysate at 20 µg

Secondary
All lanes : Goat Anti-Rabbit IgG H&L (HRP) (**ab97051**) at 1/50000 dilution

Predicted band size: 85 kDa
Observed band size: 100 kDa

Blocking and diluting buffer and concentration: 5% NFDM/TBST

The molecular weight observed is consistent with what has been described in the literature (PMID:15286660)

Exposure time: Lane 1/3: 8 seconds Lane 2: 1 second Lane 4: 3 seconds

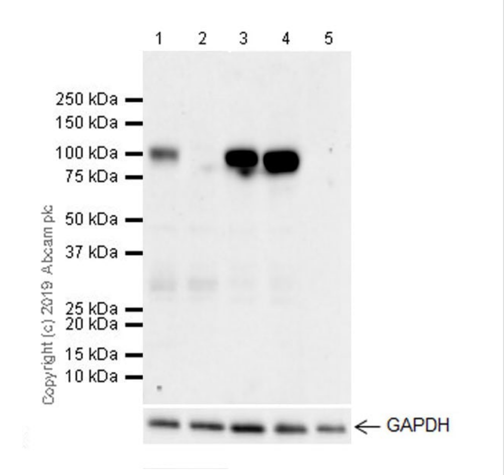

Western blot - Anti-CD10 antibody [EPR22867-118] (ab256494)

All lanes : Anti-CD10 antibody [EPR22867-118] (ab256494) at 1/1000 dilution

Lane 1 : Wild type HAP1 whole cell lysate
Lane 2 : CD10 knockout HAP1 whole cell lysate
Lane 3 : Raji (human Burkitts lymphoma B lymphocyte), whole cell lysate
Lane 4 : Ramos (human Burkitts lymphoma B lymphocyte), whole cell lysate
Lane 5 : HT-29 (human colorectal adenocarcinoma epithelial cell), whole cell lysate

Lysates/proteins at 20 µg per lane.

Secondary
Lanes 1-2 : Goat Anti-Rabbit IgG H&L (HRP) (**ab97051**)
Lanes 3-5 : Goat Anti-Rabbit IgG H&L (HRP) (**ab97051**) at 1/100000 dilution

Predicted band size: 85 kDa
Observed band size: 100 kDa

Blocking and diluting buffer and concentration: 5% NFDM/TBST

ab256494 was shown to specifically react with CD10 in wild-type HAP1 cells as signal was lost in CD10 knockout cells. Wild-type and CD10 knockout samples were subjected to SDS-PAGE. ab256494 and **ab181602** (Rabbit anti-GAPDH loading control) were incubated 1 hour at room temperature at 1/1000 dilution and 1/200,000 dilution respectively. Blots were developed with Goat Anti-Rabbit IgG, (H+L), Peroxidase conjugated (**ab97051**) secondary antibody at 1/100,000 dilution for 1 hour at room temperature before imaging.

The molecular weight observed is consistent with what has been described in the literature (PMID:15286660) Negative control: HT-29 (PMID:19828468).

Exposure time: 59 seconds

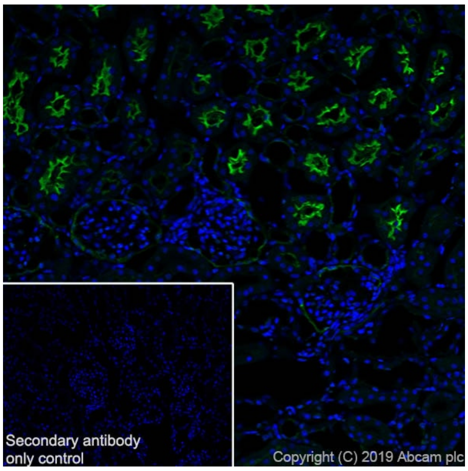

Immunohistochemistry (Frozen sections) - Anti-CD10 antibody [EPR22867-118] (ab256494)

Immunohistochemical analysis of 4% PFA fixed 0.2% Triton X-100 permeabilized frozen Rat kidney tissue labeling CD10 with ab256494 at 1/100 (5.45 µg/ml) dilution followed by **ab150077** AlexaFluor®488 Goat anti-Rabbit secondary at 1/1000 (2 µg/ml) dilution. The nuclear counterstain was DAPI (Blue). Heat mediated antigen retrieval using sodium citrate buffer (10mM citrate pH 6.0 + 0.05% Tween-20).

Secondary antibody only control: Used PBS instead of primary antibody, secondary antibody was **ab150077** AlexaFluor®488 Goat anti-Rabbit secondary at 1/1000 (2 µg/ml) dilution.

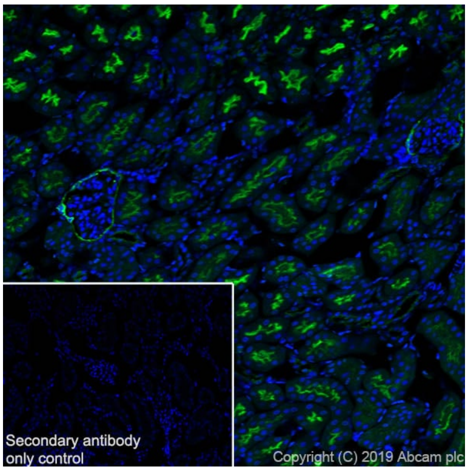

Immunohistochemistry (Frozen sections) - Anti-CD10 antibody [EPR22867-118] (ab256494)

Immunohistochemical analysis of 4% PFA fixed 0.2% Triton X-100 permeabilized frozen Mouse kidney tissue labeling CD10 with ab256494 at 1:100 (5.45 µg/ml) dilution followed by **ab150077** AlexaFluor®488 Goat anti-Rabbit secondary at 1/1000 (2 µg/ml) dilution. The nuclear counterstain was DAPI (Blue). Heat mediated antigen retrieval using sodium citrate buffer (10mM citrate pH 6.0 + 0.05% Tween-20).

Secondary antibody only control: Used PBS instead of primary antibody, secondary antibody was **ab150077** AlexaFluor®488 Goat anti-Rabbit secondary at 1:1000 (2 µg/ml) dilution.

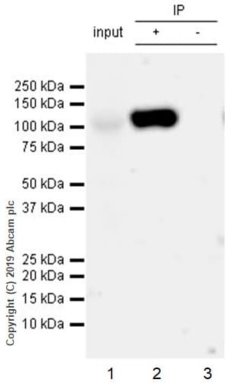

Immunoprecipitation - Anti-CD10 antibody [EPR22867-118] (ab256494)

CD10 was immunoprecipitated from 0.35 mg Raji (human Burkitt's lymphoma B lymphocyte) whole cell lysate with ab256494 at 1/30 (2ug in 0.35mg lysates). Western blot was performed on the immunoprecipitate using ab256494 at 1/1000 dilution. VeriBlot for IP Detection Reagent (HRP) (**ab131366**) was used at 1/5000 dilution.

Lane 1: Raji (human Burkitt's lymphoma B lymphocyte) whole cell lysate 10ug

Lane 2: ab256494 IP in Raji whole cell lysate

Lane 3: Rabbit monoclonal IgG (**ab172730**) instead of ab256494 in Raji whole cell lysate

Blocking and dilution buffer and concentration: 5% NFDM/TBST.

Exposure time: 3 min

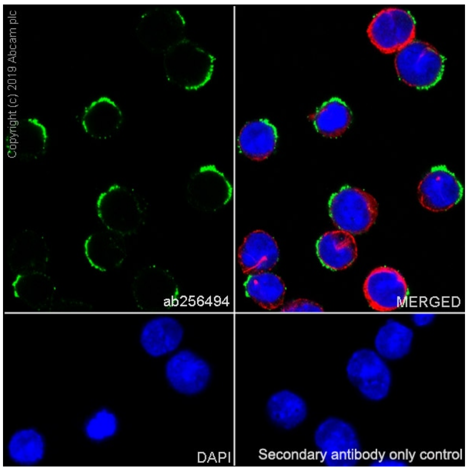

Immunocytochemistry/ Immunofluorescence - Anti-CD10 antibody [EPR22867-118] (ab256494)

Immunofluorescent analysis of 100% Methanol-fixed 2.4G2 (rat B cell lymphoma B lymphocyte) cells labelling CD10 with ab256494 at 1/500 dilution, followed by Ab256494 anti- CD10 **ab150077** AlexaFluor®488 Goat anti-Rabbit secondary antibody at 1/1000 dilution (Green). Confocal image showing staining in 2.4G2 cell line is observed. Ab195889 Anti-alpha Tubulin antibody [DM1A] - Microtubule Marker (Alexa Fluor® 594) was used to counterstain tubulin at 1/200 dilution (Red). The Nuclear counterstain was DAPI (Blue).

Secondary antibody only control: Secondary antibody is Ab256494 anti- CD10 **ab150077** AlexaFluor®488 Goat anti-Rabbit secondary at 1/1000 dilution.

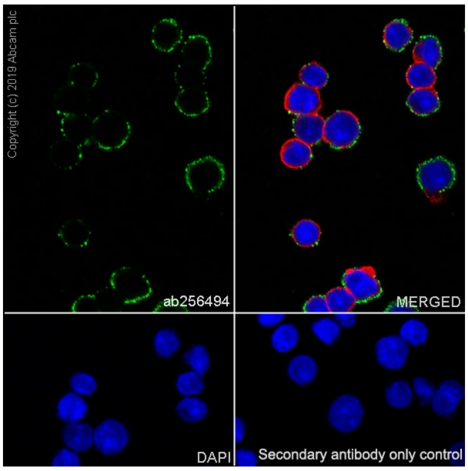

Immunocytochemistry/ Immunofluorescence - Anti-CD10 antibody [EPR22867-118] (ab256494)

Immunofluorescent analysis of 100% Methanol-fixed WEHI-231 (mouse B cell lymphoma B lymphocyte) cells labelling CD10 with ab256494 at 1/500 dilution, followed by Ab256494 anti- CD10 **ab150077** AlexaFluor®488 Goat anti-Rabbit secondary antibody at 1/1000 dilution (Green). Confocal image showing staining in WEHI-231 cell line is observed. Ab195889 Anti-alpha Tubulin antibody [DM1A] - Microtubule Marker (Alexa Fluor® 594) was used to counterstain tubulin at 1/200 dilution (Red). The Nuclear counterstain was DAPI (Blue).

Secondary antibody only control: Secondary antibody is Ab256494 anti- CD10 **ab150077** AlexaFluor®488 Goat anti-Rabbit secondary at 1/1000 dilution.

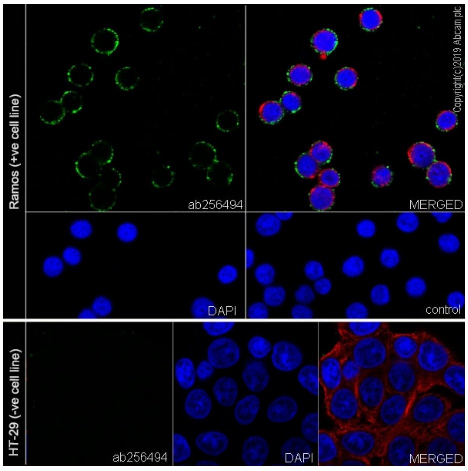

Immunocytochemistry/ Immunofluorescence - Anti-CD10 antibody [EPR22867-118] (ab256494)

Immunofluorescent analysis of 100% Methanol-fixed, Ramos (human Burkitt's lymphoma B lymphocyte) cells labelling CD10 with ab256494 at 1/500 dilution, followed by Ab256494 anti- CD10 **ab150077** AlexaFluor®488 Goat anti-Rabbit secondary antibody at 1/1000 dilution (Green).Confocal image showing membranous staining in Ramos cell line is observed. Ab195889 Anti-alpha Tubulin antibody [DM1A] - Microtubule Marker (Alexa Fluor® 594) was used to counterstain tubulin at 1/200 dilution (Red). The Nuclear counterstain was DAPI (Blue). **Negative control:** HT-29 PMID: 19828468

Secondary antibody only control: Secondary antibody is Ab256494 anti- CD10 **ab150077** AlexaFluor®488 Goat anti-Rabbit secondary at 1/1000 dilution.

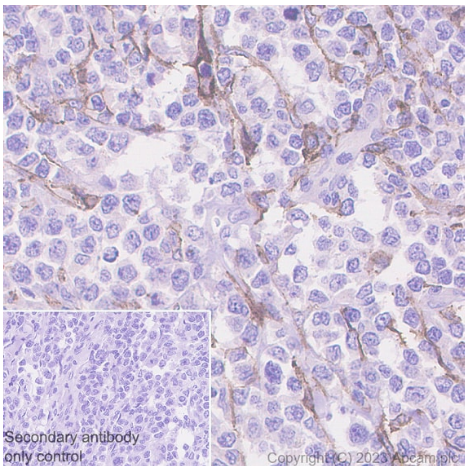

Immunohistochemistry (Formalin/PFA-fixed paraffin-embedded sections) - Anti-CD10 antibody [EPR22867-118] (ab256494)

Immunohistochemical analysis of paraffin-embedded Human diffuse large B-cell lymphoma labelling CD10 with ab256494 at 1/500 dilution, followed by a Goat Anti-Rabbit IgG H&L (HRP polymer) ready to use (**ab214880**).

Positive staining on Human diffuse large B-cell lymphoma is observed. Counter stained with hematoxylin. Secondary antibody only control: Used PBS instead of primary antibody, secondary antibody is a Goat Anti-Rabbit IgG H&L (HRP polymer) ready to use. Heat mediated antigen retrieval using **ab93684** (Tris/EDTA buffer, pH 9.0).

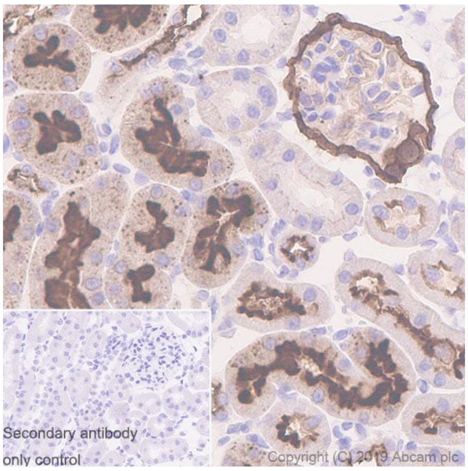

Immunohistochemistry (Formalin/PFA-fixed paraffin-embedded sections) - Anti-CD10 antibody [EPR22867-118] (ab256494)

Immunohistochemical analysis of paraffin-embedded Rat kidney tissue labeling CD10 with ab256494 at 1/500 dilution followed by a ready to use Goat Anti-Rabbit IgG H&L (HRP). Membranous staining on renal tubules and of rat kidney (PMID:10705818) is observed. Counterstained with Hematoxylin.

Secondary antibody only control: Secondary antibody is a ready to use Goat Anti-Rabbit IgG H&L (HRP).

Heat mediated antigen retrieval using **ab93684** (Tris/EDTA buffer, pH 9.0).

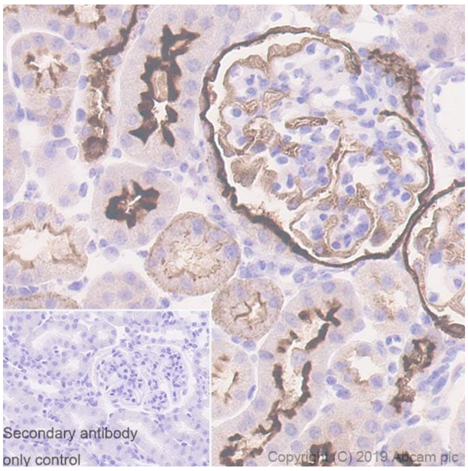

Immunohistochemistry (Formalin/PFA-fixed paraffin-embedded sections) - Anti-CD10 antibody [EPR22867-118] (ab256494)

Immunohistochemical analysis of paraffin-embedded Mouse kidney tissue labeling CD10 with ab256494 at 1/500 dilution followed by a ready to use Goat Anti-Rabbit IgG H&L (HRP). Membranous staining on renal tubules and of mouse kidney (PMID:10705818) is observed. Counterstained with Hematoxylin.

Secondary antibody only control: Secondary antibody is a ready to use Goat Anti-Rabbit IgG H&L (HRP).

Heat mediated antigen retrieval using **ab93684** (Tris/EDTA buffer, pH 9.0).

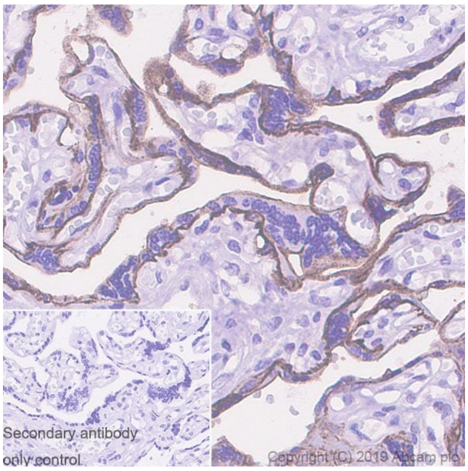

Immunohistochemistry (Formalin/PFA-fixed paraffin-embedded sections) - Anti-CD10 antibody [EPR22867-118] (ab256494)

Immunohistochemical analysis of paraffin-embedded Human placenta tissue labeling CD10 with ab256494 at 1/500 dilution followed by a ready to use Goat Anti-Rabbit IgG H&L (HRP). Membranous staining on syncytiotrophoblast layer of human placenta (PMID:11092533) is observed. Counterstained with Hematoxylin.

Secondary antibody only control: Secondary antibody is a ready to use Goat Anti-Rabbit IgG H&L (HRP).

Heat mediated antigen retrieval using **ab93684** (Tris/EDTA buffer, pH 9.0).

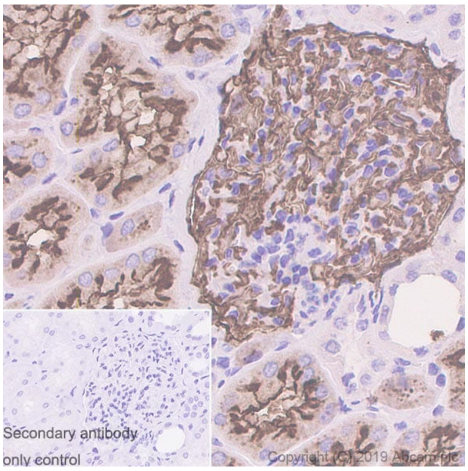

Immunohistochemistry (Formalin/PFA-fixed paraffin-embedded sections) - Anti-CD10 antibody [EPR22867-118] (ab256494)

Immunohistochemical analysis of paraffin-embedded Human kidney tissue labeling CD10 with ab256494 at 1/500 dilution followed by a ready to use Goat Anti-Rabbit IgG H&L (HRP). Positive staining on proximal convoluted tubules and glomerular epithelial cells of human kidney (PMID:10705818) is observed. Counterstained with Hematoxylin.

Secondary antibody only control: Secondary antibody is a ready to use Goat Anti-Rabbit IgG H&L (HRP).

Heat mediated antigen retrieval using **ab93684** (Tris/EDTA buffer, pH 9.0).

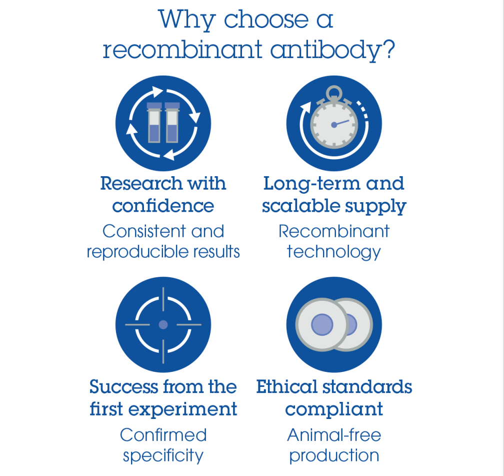

Anti-CD10 antibody [EPR22867-118] (ab256494)

Please note: All products are "FOR RESEARCH USE ONLY. NOT FOR USE IN DIAGNOSTIC PROCEDURES"

Our Abpromise to you: Quality guaranteed and expert technical support

- Replacement or refund for products not performing as stated on the datasheet
- Valid for 12 months from date of delivery
- Response to your inquiry within 24 hours

- We provide support in Chinese, English, French, German, Japanese and Spanish
- Extensive multi-media technical resources to help you
- We investigate all quality concerns to ensure our products perform to the highest standards

If the product does not perform as described on this datasheet, we will offer a refund or replacement. For full details of the Abpromise, please visit **https://www.abcam.com/abpromise** or contact our technical team.

Terms and conditions

- Guarantee only valid for products bought direct from Abcam or one of our authorized distributors

abcam

Product datasheet

Anti-PPAR alpha antibody - ChIP Grade ab227074

★★★★★ 3 Abreviews 10 References 3 Images

Overview

**Product name**	Anti-PPAR alpha antibody - ChIP Grade
**Description**	Rabbit polyclonal to PPAR alpha - ChIP Grade
**Host species**	Rabbit
**Tested applications**	**Suitable for:** WB, IP, ChIP
**Species reactivity**	**Reacts with:** Rat, Human
	**Predicted to work with:** Dog, Pig, Rhesus monkey
**Immunogen**	Recombinant fragment within Human PPAR alpha (N terminal). The exact sequence is proprietary.<br>Database link: Q07869
**Positive control**	ChIP: HepG2 chromatin extract. IP: HepG2 whole cell lysate (ab7900). WB: Rat primary hepatocyte lysate.
**General notes**	The Life Science industry has been in the grips of a reproducibility crisis for a number of years. Abcam is leading the way in addressing this with our range of recombinant monoclonal antibodies and knockout edited cell lines for gold-standard validation. Please check that this product meets your needs before purchasing.<br>If you have any questions, special requirements or concerns, please send us an inquiry and/or contact our Support team ahead of purchase. Recommended alternatives for this product can be found below, along with publications, customer reviews and Q&As

Properties

**Form**	Liquid
**Storage instructions**	Shipped at 4°C. Store at +4°C short term (1-2 weeks). Upon delivery aliquot. Store at -20°C long term. Avoid freeze / thaw cycle.
**Storage buffer**	pH: 7.00<br>Preservative: 0.025% Proclin 300<br>Constituents: 79% PBS, 20% Glycerol (glycerin, glycerine)
**Purity**	Immunogen affinity purified
**Clonality**	Polyclonal
**Isotype**	IgG

Applications

The Abpromise guarantee Our **Abpromise guarantee** covers the use of ab227074 in the following tested applications.

The application notes include recommended starting dilutions; optimal dilutions/concentrations should be determined by the end user.

Application	Abreviews	Notes
**WB**	★★★★★ (1)	1/500 - 1/3000. Predicted molecular weight: 52 kDa.
**IP**		1/100 - 1/500.
**ChIP**		Use at an assay dependent concentration. Use 5 µg per reaction.

Target

Function Ligand-activated transcription factor. Key regulator of lipid metabolism. Activated by the endogenous ligand 1-palmitoyl-2-oleoyl-sn-glycerol-3-phosphocholine (16:0/18:1-GPC). Activated by oleylethanolamide, a naturally occurring lipid that regulates satiety (By similarity). Receptor for peroxisome proliferators such as hypolipidemic drugs and fatty acids. Regulates the peroxisomal beta-oxidation pathway of fatty acids. Functions as transcription activator for the ACOX1 and P450 genes. Transactivation activity requires heterodimerization with RXRA and is antagonized by NR2C2.

Tissue specificity Skeletal muscle, liver, heart and kidney.

Sequence similarities Belongs to the nuclear hormone receptor family. NR1 subfamily.
Contains 1 nuclear receptor DNA-binding domain.

Cellular localization Nucleus.

Images

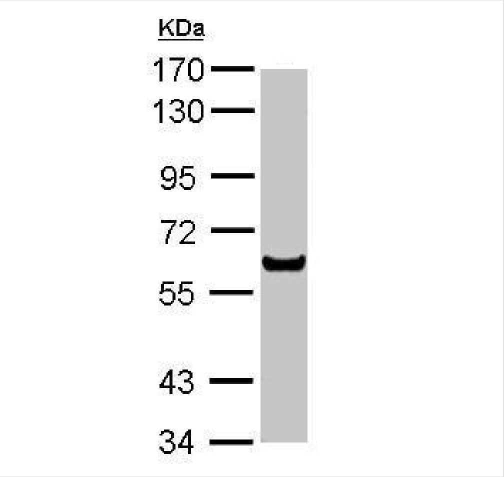

Western blot - Anti-PPAR alpha antibody - ChIP Grade (ab227074)

Anti-PPAR alpha antibody - ChIP Grade (ab227074) at 1/1000 dilution + Rat primary hepatocyte lysate at 30 µg

Secondary

HRP-conjugated anti-rabbit IgG

Predicted band size: 52 kDa

7.5% SDS-PAGE gel.

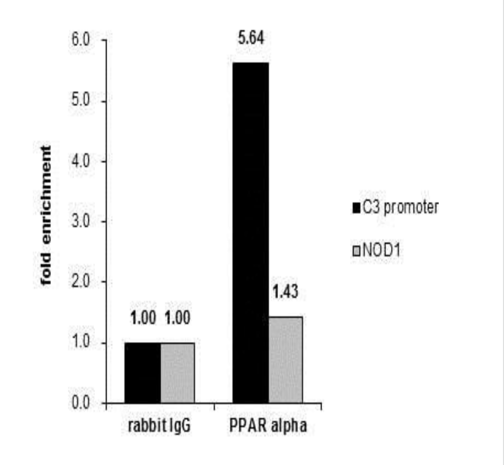

ChIP - Anti-PPAR alpha antibody - ChIP Grade (ab227074)

Cross-linked ChIP was performed with HepG2 (human liver hepatocellular carcinoma cell line) chromatin extract and 5 µg of either control rabbit IgG or ab227074. The precipitated DNA was detected by PCR with primer set targeting to C3 promotor or NOD1 gene.

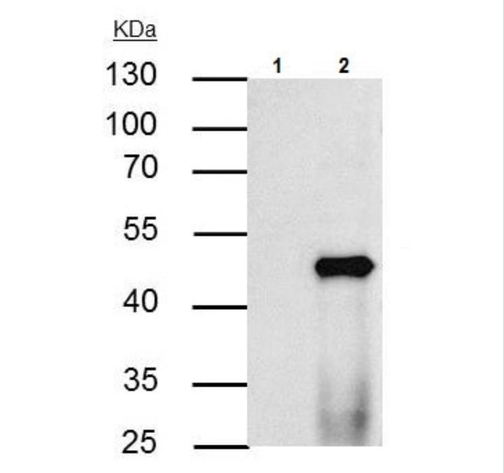

Immunoprecipitation - Anti-PPAR alpha antibody - ChIP Grade (ab227074)

PPAR alpha was immunoprecipitated from HepG2 (human liver hepatocellular carcinoma cell line) whole cell extract with ab227074 at 1/100 dilution. Western blot was performed from the immunoprecipitate using ab227074 at 1/1000 dilution.

Lane 1: Control IgG IP in HepG2 whole cell extract.

Lane 2: ab227074 IP in HepG2 whole cell extract.

Please note: All products are "FOR RESEARCH USE ONLY. NOT FOR USE IN DIAGNOSTIC PROCEDURES"

Our Abpromise to you: Quality guaranteed and expert technical support

- Replacement or refund for products not performing as stated on the datasheet
- Valid for 12 months from date of delivery
- Response to your inquiry within 24 hours

- We provide support in Chinese, English, French, German, Japanese and Spanish
- Extensive multi-media technical resources to help you
- We investigate all quality concerns to ensure our products perform to the highest standards

If the product does not perform as described on this datasheet, we will offer a refund or replacement. For full details of the Abpromise, please visit **https://www.abcam.com/abpromise** or contact our technical team.

Terms and conditions

- Guarantee only valid for products bought direct from Abcam or one of our authorized distributors

abcam

Product datasheet

Anti-PPAR gamma antibody ab209350

★★★★★ 2 Abreviews 55 References 8 Images

Overview

**Product name**	Anti-PPAR gamma antibody
**Description**	Rabbit polyclonal to PPAR gamma
**Host species**	Rabbit
**Specificity**	No significant homology with PPAR alpha or NUC1.
**Tested applications**	**Suitable for:** ICC, WB
**Species reactivity**	**Reacts with:** Mouse, Rat, Human
**Immunogen**	Synthetic peptide corresponding to Mouse PPAR gamma aa 250-350.<br>Database link: P37238<br>Run BLAST with Run BLAST with
**General notes**	The Life Science industry has been in the grips of a reproducibility crisis for a number of years. Abcam is leading the way in addressing this with our range of recombinant monoclonal antibodies and knockout edited cell lines for gold-standard validation. Please check that this product meets your needs before purchasing.<br>If you have any questions, special requirements or concerns, please send us an inquiry and/or contact our Support team ahead of purchase. Recommended alternatives for this product can be found below, along with publications, customer reviews and Q&As

Properties

**Form**	Liquid
**Storage instructions**	Shipped at 4°C. Store at +4°C short term (1-2 weeks). Upon delivery aliquot. Store at -20°C long term. Avoid freeze / thaw cycle.
**Storage buffer**	Preservative: 0.05% Sodium azide<br>Constituent: PBS
**Purity**	Whole antiserum
**Clonality**	Polyclonal
**Isotype**	IgG

Applications

The Abpromise guarantee Our **Abpromise guarantee** covers the use of ab209350 in the following tested applications.

The application notes include recommended starting dilutions; optimal dilutions/concentrations should be determined by the end user.

Application	Abreviews	Notes
ICC		1/250.
WB	★★★★★ (1)	1/500 - 1/1000. Predicted molecular weight: 57 kDa.

Target

Function Receptor that binds peroxisome proliferators such as hypolipidemic drugs and fatty acids. Once activated by a ligand, the receptor binds to a promoter element in the gene for acyl-CoA oxidase and activates its transcription. It therefore controls the peroxisomal beta-oxidation pathway of fatty acids. Key regulator of adipocyte differentiation and glucose homeostasis.

Tissue specificity Highest expression in adipose tissue. Lower in skeletal muscle, spleen, heart and liver. Also detectable in placenta, lung and ovary.

Involvement in disease Note=Defects in PPARG can lead to type 2 insulin-resistant diabetes and hyptertension. PPARG mutations may be associated with colon cancer.
Defects in PPARG may be associated with susceptibility to obesity (OBESITY) [MIM:601665]. It is a condition characterized by an increase of body weight beyond the limitation of skeletal and physical requirements, as the result of excessive accumulation of body fat.
Defects in PPARG are the cause of familial partial lipodystrophy type 3 (FPLD3) [MIM:604367]. Familial partial lipodystrophies (FPLD) are a heterogeneous group of genetic disorders characterized by marked loss of subcutaneous (sc) fat from the extremities. Affected individuals show an increased preponderance of insulin resistance, diabetes mellitus and dyslipidemia.
Genetic variations in PPARG can be associated with susceptibility to glioma type 1 (GLM1) [MIM:137800]. Gliomas are central nervous system neoplasms derived from glial cells and comprise astrocytomas, glioblastoma multiforme, oligodendrogliomas, and ependymomas. Note=Polymorphic PPARG alleles have been found to be significantly over-represented among a cohort of American patients with sporadic glioblastoma multiforme suggesting a possible contribution to disease susceptibility.

Sequence similarities Belongs to the nuclear hormone receptor family. NR1 subfamily.
Contains 1 nuclear receptor DNA-binding domain.

Cellular localization Nucleus.

Images

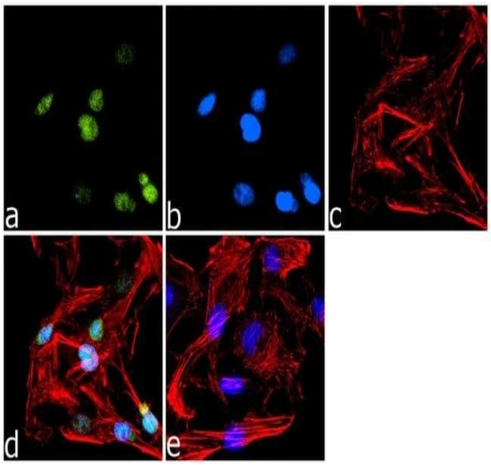

Immunocytochemistry - Anti-PPAR gamma antibody (ab209350)

Immunocytochemistry analysis of 70% confluent log phase HeLa cells labeling PPAR Gamma with ab209350. The cells were fixed with 4% paraformaldehyde for 15 minutes, permeabilized with 0.25% Triton™ X-100 for 10 minutes, and blocked with 5% BSA for 1 hour at room temperature. The cells were labeled with ab209350 at 1/250 dilution in 0.1% BSA and incubated for 3 hours at room temperature and then labeled with Goat anti-Rabbit IgG (H+L) Superclonal™ Secondary Antibody, Alexa Fluor® 488 conjugate at a dilution of 1/2000 for 45 minutes at room temperature (Panel a: green). Nuclei (Panel b: blue) were stained with SlowFade® Gold Antifade Mountant with DAPI. F-actin (Panel c: red) was stained with Rhodamine Phalloidin, 1/300. Panel d is a merged image showing Nuclear localization. Panel e is a no primary antibody control. The images were captured at 60X magnification.

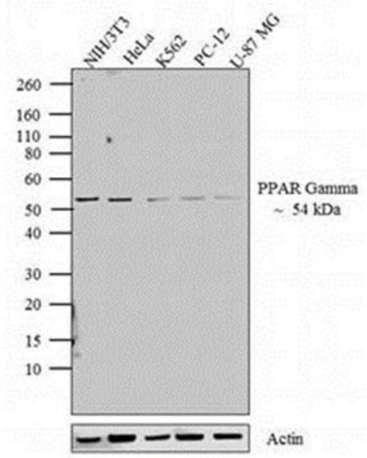

Western blot - Anti-PPAR gamma antibody (ab209350)

All lanes : Anti-PPAR gamma antibody (ab209350)

Lane 1 : NIH/3T3 cell lysate
Lane 2 : HeLa cell lysate
Lane 3 : K562 cell lysate
Lane 4 : PC-12 cell lysate
Lane 5 : U-87 MG cell lysate

Predicted band size: 57 kDa

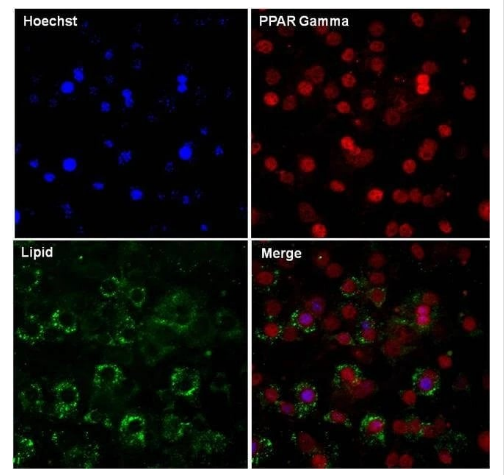

Immunocytochemistry - Anti-PPAR gamma antibody (ab209350)

Immunocytochemistry analysis of 3T3-L1 differentiated day 7 cells labeling PPAR gamma-2 (red) with ab209350. The cells were fixed with 4% paraformaldehyde in PBS for 15 minutes at room temperature, permeabilized with 0.1% Triton X-100 for 15 minutes, and blocked with 3% BSA for 30 minutes at room temperature. Cells were stained with ab209350 at a dilution of 1/200 in blocking buffer for 1 hour at room temperature, and then incubated with a Goat anti-Rabbit IgG (H+L) Secondary Antibody, Dylight 680 at a dilution of 1/1000 for at least 30 minutes at room temperature in the dark (red). Lipids (green) were stained with HCS LipidTOX neutral lipid stain at a dilution of 1/200 for at least 30 minutes at room temperature in the dark. Nuclei (blue) were stained with Hoechst 33342. Images were taken on a EVOS FL Auto Imaging System at 20X magnification.

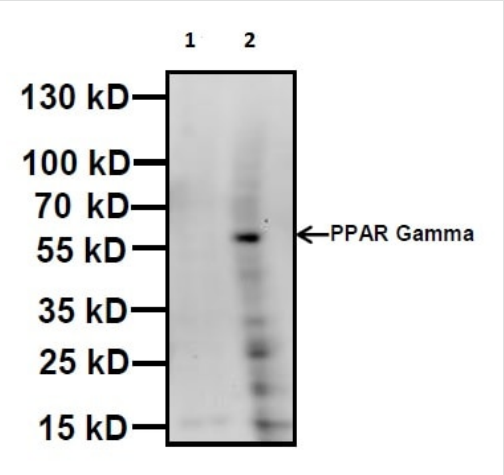

Western blot - Anti-PPAR gamma antibody (ab209350)

All lanes : Anti-PPAR gamma antibody (ab209350) at 1/500 dilution

Lane 1 : 3T3
Lane 2 : 3T3-L1 differentiated day 7

Lysates/proteins at 20 µg per lane.

Predicted band size: 57 kDa

Specific band detected in 3T3-L1 day 7 differentiated lysate with no reactivity in 3T3 lysate.

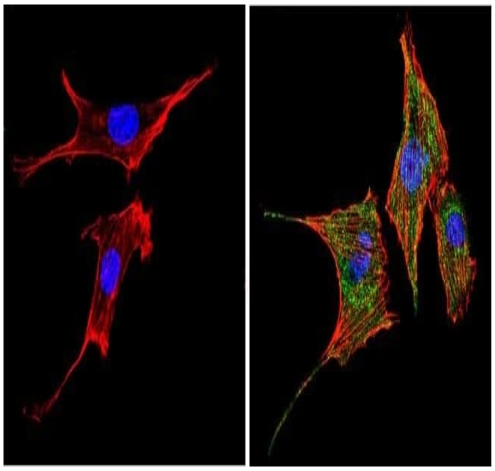

Immunocytochemistry - Anti-PPAR gamma antibody (ab209350)

Immunocytochemistry analysis of PPAR gamma (green) showing positive staining in the nucleus and cytoplasm of NIH-3T3 cells (right) compared with a negative control in the absence of primary antibody (left). Formalin-fixed cells were permeabilized with 0.1% Triton X-100 in TBS for 5-10 minutes, blocked with 3% BSA-PBS for 30 minutes at room temperature and probed with ab209350 in 3% BSA-PBS at a dilution of 1/200 and incubated overnight at 4 °C in a humidified chamber. Cells were washed with PBST and incubated with a DyLight 488-conjugated goat-anti-rabbit IgG secondary antibody in PBS at room temperature in the dark. F-actin (red) was stained with a fluorescent red phalloidin and nuclei (blue) were stained with DAPI for 5-10 minutes in the dark. Images were taken at a magnification of 60x.

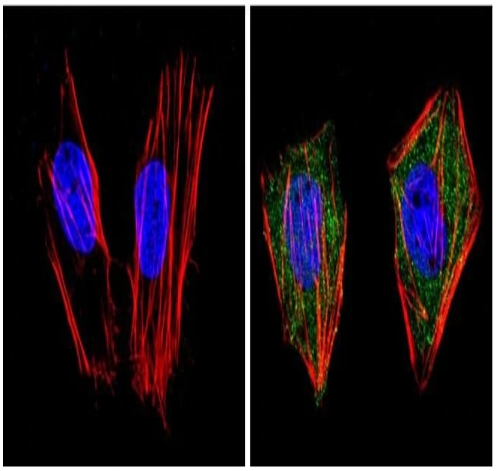

Immunocytochemistry - Anti-PPAR gamma antibody (ab209350)

Immunocytochemistry analysis of PPAR gamma (green) showing positive staining in the nucleus and cytoplasm of Hela cells (right) compared with a negative control in the absence of primary antibody (left). Formalin-fixed cells were permeabilized with 0.1% Triton X-100 in TBS for 5-10 minutes, blocked with 3% BSA-PBS for 30 minutes at room temperature and probed with ab209350 in 3% BSA-PBS at a dilution of 1/200 and incubated overnight at 4 °C in a humidified chamber. Cells were washed with PBST and incubated with a DyLight 488-conjugated goat-anti-rabbit IgG secondary antibody in PBS at room temperature in the dark. F-actin (red) was stained with a fluorescent red phalloidin and nuclei (blue) were stained with DAPI for 5-10 minutes in the dark. Images were taken at a magnification of 60x.

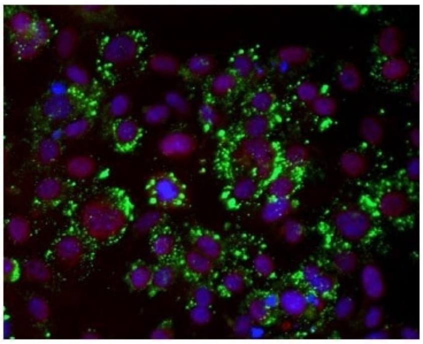

Immunocytochemistry - Anti-PPAR gamma antibody (ab209350)

Immunocytochemical analysis of PPAR gamma using ab209350 at the dilution 1/200. 3T3-L1 cells providing positive signal have been differentiated for 7 days.

PPAR gamma is shown in red, lipid droplets (that indicates the proper differentiation of the cells) are shown in green.

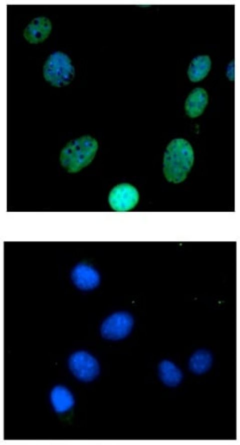

Immunocytochemistry - Anti-PPAR gamma antibody (ab209350)

Immunocytochemical analysis of PPAR gamma using ab209350 at the dilution 1/200.The image at the top shows 3T3-L1 cells differntiated (for 7 days) where PPAR gamma is shown in green. The image below shows 3T3-L1 undifferentiated cells where no PPAR gamma is detected.

Please note: All products are "FOR RESEARCH USE ONLY. NOT FOR USE IN DIAGNOSTIC PROCEDURES"

Our Abpromise to you: Quality guaranteed and expert technical support

- Replacement or refund for products not performing as stated on the datasheet
- Valid for 12 months from date of delivery
- Response to your inquiry within 24 hours

- We provide support in Chinese, English, French, German, Japanese and Spanish
- Extensive multi-media technical resources to help you
- We investigate all quality concerns to ensure our products perform to the highest standards

If the product does not perform as described on this datasheet, we will offer a refund or replacement. For full details of the Abpromise, please visit **https://www.abcam.com/abpromise** or contact our technical team.

Terms and conditions

- Guarantee only valid for products bought direct from Abcam or one of our authorized distributors

Safety Data Sheet (SDS) According to the REACH Regulation (EC) No. 1907/2006

Issuing Date: 2017-07-10 **Revision Date:** 2023-11-16 **Version:** 2

SECTION 1: Identification of the substance/mixture and of the company/undertaking

1.1. Product identifier

Product No 64099

Product name LRP1 Antibody

Contains

Chemical name	Index No.	CAS No
glycerol (30-60)	Not Listed	56-81-5

1.2. Relevant identified uses of the substance or mixture and uses advised against

Identified uses For Research Use Only. Not for Use in Diagnostic Procedures.

1.3. Details of the supplier of the safety data sheet

Importer
Cell Signaling Technology Europe B.V.
Dellaertweg 9b
2316 WZ Leiden
The Netherlands
TEL: +31 (0)71 7200 200
FAX: +31 (0)71 891 0019

Manufacturer
Cell Signaling Technology, Inc.
3 Trask Lane
Danvers, MA 01923
United States
TEL: +1 978 867 2300
FAX: +1 978 867 2400

Website www.cellsignal.com
E-mail Address info@cellsignal.eu

1.4. Emergency telephone number

CHEMTREC 24 hours a day, 7 days a week, 365 days a year
+1 703 527 3887 (INTERNATIONAL) +1 800 424 9300 (NORTH AMERICA)

Europe 112

SECTION 2: Hazards identification

2.1. Classification of the substance or mixture

Regulation (EC) No. 1272/2008

This substance is classified as not hazardous according to regulation (EC) 1272/2008 [CLP]

2.2. Label elements

Signal word
None.

Hazard statement(s)
None.

Precautionary statement(s)
None.

2.3. Other hazards

May produce an allergic reaction.

For the full text of the H-phrases & EUH-phrases mentioned in this Section, see Section 16

SECTION 3: Composition/information on ingredients

Chemical nature Mixture

Chemical name	CAS No	Weight-%	EC No	Classification (1272/2008)	REACH Registration Number
glycerol	56-81-5	30-60	200-289-5	-	no data available

For the full text of the H-phrases & EUH-phrases mentioned in this Section, see Section 16

SECTION 4: First aid measures

4.1. Description of first aid measures

General advice Use first aid treatment according to the nature of the injury. When symptoms persist or in all cases of doubt seek medical advice.
Inhalation Move to fresh air.
Skin contact Wash skin with soap and water.
Eye contact Rinse thoroughly with plenty of water, also under the eyelids.
Ingestion Clean mouth with water and afterwards drink plenty of water.

4.2. Most important symptoms and effects, both acute and delayed

No information available.

4.3. Indication of any immediate medical attention and special treatment needed

Notes to physician Treat symptomatically.

SECTION 5: Firefighting measures

5.1. Extinguishing media

Suitable Extinguishing Media Use extinguishing measures that are appropriate to local circumstances and the surrounding environment.
Unsuitable Extinguishing Media No information available.

5.2. Special hazards arising from the substance or mixture

Thermal decomposition can lead to release of irritating gases and vapors.

5.3. Advice for firefighters

Wear self-contained breathing apparatus and protective suit. Use personal protective equipment.

SECTION 6: Accidental release measures

6.1. Personal precautions, protective equipment and emergency procedures

For non-emergency personnel Avoid contact with skin, eyes and clothing. Use personal protective equipment. For personal protection see section 8.

For emergency responders Use personal protection recommended in Section 8.

6.2. Environmental precautions

Prevent further leakage or spillage if safe to do so. Prevent product from entering drains. Prevent entry into waterways, sewers, basements or confined areas.

6.3. Methods and material for containment and cleaning up

Methods for containment Prevent further leakage or spillage if safe to do so.

Methods for cleaning up Soak up with inert absorbent material. Pick up and transfer to properly labeled containers.

6.4. Reference to other sections

See Sections 8 & 13 for additional information.

SECTION 7: Handling and storage

7.1. Precautions for safe handling

Handle in accordance with good industrial hygiene and safety practice. Wear personal protective equipment. Avoid contact with skin, eyes and clothing. Remove and wash contaminated clothing before re-use.

7.2. Conditions for safe storage, including any incompatibilities

Keep container tightly closed in a dry and well-ventilated place.

7.3. Specific end use(s)

Use as a laboratory reagent.

SECTION 8: Exposure controls/personal protection

8.1. Control parameters

Chemical name	European Union	United Kingdom	France	Spain	Germany
glycerol		STEL 30 mg/m^3<br>TWA 10 mg/m^3	TWA 10 mg/m^3	TWA 10 mg/m^3	Ceiling / Peak: 400 mg/m^3<br>TWA: 200 mg/m^3
**Chemical name**	**Italy**	**Portugal**	**Netherlands**	**Finland**	**Denmark**
glycerol		TWA 10 mg/m^3		TWA 20 mg/m^3	
**Chemical name**	**Austria**	**Switzerland**	**Poland**	**Norway**	**Ireland**
glycerol		SS-C**<br>TWA 50 mg/m^3<br>STEL 100 mg/m^3	TWA 10 mg/m^3		TWA 10 mg/m^3<br>STEL 30 mg/m^3

8.2. Exposure controls

Appropriate engineering controls
Showers, eyewash stations, and ventilation systems.

Individual protection measures, such as personal protective equipment

**Eye/face protection**	Safety glasses with side-shields.
**Skin protection**	Wear protective gloves and protective clothing
**Hand protection**	Impervious gloves.
**Other**	Wear suitable protective clothing.
**Respiratory protection**	When workers are facing concentrations above the exposure limit they must use appropriate certified respirators.

Environmental Exposure Controls
No information available.

SECTION 9: Physical and chemical properties

9.1. Information on basic physical and chemical properties

**Physical state**	Liquid - Clear
**Color**	Colorless
**Odor**	No information available

Property	Values	Remarks • Method
**pH**	7.5	@ 20 °C
**Melting point/freezing point**	No information available	No information available
**Boiling point or initial boiling point and boiling range**	No information available	No information available
**Flash point**	No information available	No information available.
**Evaporation rate**	No information available	No information available
**Flammability**	No information available	No information available
**Upper/lower flammability or explosive limits**	No information available /	No information available
**Vapor pressure**	No information available	No information available
**Relative vapor density**	No information available	No information available
**Density and/or relative density**	No information available	No information available
**Solubility**	No information available.	No information available
**Partition coefficient: n-octanol/water**	No information available	No information available
**Autoignition temperature**	No information available	No information available
**Decomposition temperature**	No information available	No information available.
**Viscosity**	No information available	No information available
**Explosive properties**	No information available	No information available
**Oxidizing properties**	No information available	No information available

9.2. Other information

**Softening point**	No information available
**Molecular Weight**	No information available
**Solubility in other solvents**	No information available
**VOC content**	No information available
**Liquid Density**	No information available

SECTION 10: Stability and reactivity

10.1. Reactivity

No information available.

10.2. Chemical stability

Stable under normal conditions.

10.3. Possibility of hazardous reactions

Hazardous polymerization Hazardous polymerization does not occur.
Hazardous reactions None under normal processing.

10.4. Conditions to avoid

Extremes of temperature and direct sunlight.

10.5. Incompatible materials

No information available.

10.6. Hazardous decomposition products

None under normal use conditions.

SECTION 11: Toxicological information

11.1. Information on hazard classes as defined in Regulation (EC) No 1272/2008

This product is for experimental uses only. The product has not been completely analyzed and all of the hazards may not be known. Please use caution while handling this product.

Chemical name	LD50 Oral	LD50 Dermal	LC50 Inhalation
glycerol	= 12600 mg/kg (Rat)	> 10 g/kg (Rabbit)	> 570 mg/m³ (Rat) 1 h

Information on likely routes of exposure

Inhalation Avoid breathing vapors or mists.
Eye contact Avoid contact with eyes.
Skin contact Avoid contact with skin.
Ingestion Ingestion may cause gastrointestinal irritation, nausea, vomiting and diarrhea.

Symptoms No information available.

Skin corrosion/irritation No information available.
Serious eye damage/eye irritation No information available.
Sensitization No information available.
Mutagenic effects No information available.
Carcinogenicity No information available.

Reproductive toxicity No information available.
STOT - single exposure No information available.
STOT - repeated exposure No information available.
Aspiration Hazard No information available.

11.2. Information on other hazards

No information available.

SECTION 12: Ecological information

12.1. Toxicity

Chemical name	Toxicity to algae	Toxicity to fish	Toxicity to daphnia and other aquatic invertebrates
glycerol	-	LC50 51 - 57 mL/L (Oncorhynchus mykiss) 96 h	EC50 500 mg/L (Daphnia magna) 24 h

12.2. Persistence and degradability

No information available

12.3. Bioaccumulative potential

Bioaccumulation

Chemical name	Octanol-Water Partition Coefficient
glycerol	-1.76

Bioconcentration factor (BCF) No information available.

12.4. Mobility in soil

No information available.

12.5. Results of PBT and vPvB assessment

No information available.

12.6. Endocrine disrupting properties

This product does not contain any known or suspected endocrine disruptors

12.7. Other adverse effects

No information available

SECTION 13: Disposal considerations

13.1. Waste treatment methods

Waste from residues / unused products Dispose of in accordance with local regulations.

Contaminated packaging Empty containers should be taken to an approved waste handling site for recycling or disposal.

Other information Waste codes should be assigned by the user based on the application for which the product was used.

SECTION 14: Transport information

IMDG/IMO

**14.1 UN number**	Not regulated
**14.2 UN proper shipping name**	Not regulated
**14.3 Transport hazard class(es)**	Not regulated
**14.4 Packing group**	Not regulated
**14.5 Environmental hazards**	None
**14.6 Special precautions for user**	None
**14.7 Maritime transport in bulk according to IMO instruments**	Not regulated

ADR/RID

**14.1 UN number**	Not regulated
**14.2 UN proper shipping name**	Not regulated
**14.3 Transport hazard class(es)**	Not regulated
**14.4 Packing group**	Not regulated
**14.5 Environmental hazards**	None
**14.6 Special precautions for user**	None

IATA

**14.1 UN number**	Not regulated
**14.2 UN proper shipping name**	Not regulated
**14.3 Transport hazard class(es)**	Not regulated
**14.4 Packing group**	Not regulated
**14.5 Environmental hazards**	None
**14.6 Special precautions for user**	None

SECTION 15: Regulatory information

15.1. Safety, health and environmental regulations/legislation specific for the substance or mixture

Candidate List of Substances of Very High Concern for Authorization Information

This product does not contain Substances of Very High Concern (SVHC).

SEVESO Directive Information

This product does not contain substances identified in the SEVESO Directive.

International inventories

**TSCA 8(b)**	-
**DSL/NDSL**	Complies
**EINECS/ELINCS**	-
**ENCS**	-
**IECSC**	Complies
**KECL**	-
**PICCS**	-
**AICS**	Complies

International inventories legend

TSCA - United States Toxic Substances Control Act Section 8(b) Inventory
DSL/NDSL - Canadian Domestic Substances List/Non-Domestic Substances List
EINECS/ELINCS - European Inventory of Existing Commercial Chemical Substances/EU List of Notified Chemical Substances
ENCS - Japan Existing and New Chemical Substances
IECSC - China Inventory of Existing Chemical Substances
KECL - Korean Existing and Evaluated Chemical Substances
PICCS - Philippines Inventory of Chemicals and Chemical Substances
AICS - Australian Inventory of Chemical Substances

15.2. Chemical safety assessment

For this substance a chemical safety assessment has not been carried out

SECTION 16: Other information

Full text of H-Statements referred to under Sections 2 and 3

This substance/mixture does not meet the criteria for classification in accordance with Regulation (EC) No. 1272/2008

Classification procedure: Expert judgment and weight of evidence determination.
Issuing Date: 2017-07-10
Revision Date: 2023-11-16

Disclaimer

The information provided in this Safety Data Sheet is correct to the best of our knowledge, information and belief at the date of its publication. The information given is designed only as a guidance for safe handling, use, processing, storage, transportation, disposal and release and is not to be considered a warranty or quality specification. The information relates only to the specific material designated and may not be valid for such material used in combination with any other materials or in any process, unless specified in the text.

Safety Data Sheet (SDS) According to the REACH Regulation (EC) No. 1907/2006

Issuing Date: 2017-07-10 **Revision Date:** 2022-12-14 **Version:** 3

SECTION 1: Identification of the substance/mixture and of the company/undertaking

1.1. Product identifier

Product No 2118

Product name GAPDH (14C10) Rabbit mAb

Contains

Chemical name	Index No.	CAS No
glycerol (30-60)	Not Listed	56-81-5
sodium azide (<0.02)	011-004-00-7	26628-22-8

1.2. Relevant identified uses of the substance or mixture and uses advised against

Identified uses For research use only

1.3. Details of the supplier of the safety data sheet

Importer
Cell Signaling Technology Europe B.V.
Dellaertweg 9b
2316 WZ Leiden
The Netherlands
TEL: +31 (0)71 7200 200
FAX: +31 (0)71 891 0019

Manufacturer
Cell Signaling Technology, Inc.
3 Trask Lane
Danvers, MA 01923
United States
TEL: +1 978 867 2300
FAX: +1 978 867 2400

Website www.cellsignal.com
E-mail Address info@cellsignal.eu

1.4. Emergency telephone number

CHEMTREC 24 hours a day, 7 days a week, 365 days a year
+1 703 527 3887 (INTERNATIONAL) +1 800 424 9300 (NORTH AMERICA)

Europe 112

SECTION 2: Hazards identification

2.1. Classification of the substance or mixture

Regulation (EC) No. 1272/2008

This substance is classified as not hazardous according to regulation (EC) 1272/2008 [CLP]

2.2. Label elements

Signal word

None.

Hazard statement(s)
None.

Precautionary statement(s)
None.

2.3. Other hazards

May produce an allergic reaction.

For the full text of the H-phrases & EUH-phrases mentioned in this Section, see Section 16

SECTION 3: Composition/information on ingredients

Chemical name	CAS No	Weight-%	EC No	Classification (1272/2008)	REACH Registration Number
glycerol	56-81-5	30-60	200-289-5	-	no data available
sodium azide	26628-22-8	<0.02	247-852-1	Acute Tox. 2 (H300) Aquatic Acute 1 (H400) Aquatic Chronic 1 (H410) (EUH032)	no data available

For the full text of the H-phrases & EUH-phrases mentioned in this Section, see Section 16

SECTION 4: First aid measures

4.1. Description of first aid measures

General advice Use first aid treatment according to the nature of the injury. When symptoms persist or in all cases of doubt seek medical advice.
Inhalation Move to fresh air.
Skin contact Wash skin with soap and water.
Eye contact Rinse thoroughly with plenty of water, also under the eyelids.
Ingestion Clean mouth with water and afterwards drink plenty of water.

4.2. Most important symptoms and effects, both acute and delayed

Symptoms of allergic reaction may include rash, itching, swelling, trouble breathing, tingling of the hands and feet, dizziness, lightheadedness, chest pain, muscle pain, or flushing.

4.3. Indication of any immediate medical attention and special treatment needed

Notes to physician Treat symptomatically.

SECTION 5: Firefighting measures

5.1. Extinguishing media

Suitable Extinguishing Media Use extinguishing measures that are appropriate to local circumstances and the surrounding environment.
Unsuitable Extinguishing Media None.

5.2. Special hazards arising from the substance or mixture

No information available.

5.3. Advice for firefighters

Wear self-contained breathing apparatus and protective suit. Use personal protective equipment.

SECTION 6: Accidental release measures

6.1. Personal precautions, protective equipment and emergency procedures

For non-emergency personnel Avoid contact with skin, eyes and clothing. Use personal protective equipment. For personal protection see section 8.

For emergency responders Use personal protection recommended in Section 8.

6.2. Environmental precautions

Prevent further leakage or spillage if safe to do so. Prevent product from entering drains. Prevent entry into waterways, sewers, basements or confined areas.

6.3. Methods and material for containment and cleaning up

Methods for containment Prevent further leakage or spillage if safe to do so.

Methods for cleaning up Soak up with inert absorbent material. Pick up and transfer to properly labeled containers.

6.4. Reference to other sections

See Sections 8 & 13 for additional information.

SECTION 7: Handling and storage

7.1. Precautions for safe handling

Handle in accordance with good industrial hygiene and safety practice. Wear personal protective equipment. Avoid contact with skin, eyes and clothing. Remove and wash contaminated clothing before re-use.

7.2. Conditions for safe storage, including any incompatibilities

Keep container tightly closed in a dry and well-ventilated place.

7.3. Specific end use(s)

Use as a laboratory reagent.

SECTION 8: Exposure controls/personal protection

8.1. Control parameters

Chemical name	European Union	United Kingdom	France	Spain	Germany
glycerol		STEL 30 mg/m ^{3}<br>TWA 10 mg/m ^{3}	TWA 10 mg/m ^{3}	TWA 10 mg/m ^{3}	Ceiling / Peak: 400 mg/m^3<br>TWA: 200 mg/m^3
sodium azide	TWA 0.1 mg/m ^{3}<br>STEL 0.3 mg/m ^{3}<br>S*	STEL 0.3 mg/m ^{3}<br>TWA 0.1 mg/m ^{3}<br>Skin	TWA 0.1 mg/m ^{3}<br>STEL 0.3 mg/m ^{3}<br>P*	TWA 0.1 mg/m ^{3}<br>STEL 0.3 mg/m ^{3}<br>S*	TWA: 0.2 mg/m^3<br>Ceiling / Peak: 0.4 mg/m^3
**Chemical name**	**Italy**	**Portugal**	**Netherlands**	**Finland**	**Denmark**
glycerol		TWA 10 mg/m ^{3}		TWA 20 mg/m ^{3}	
sodium azide	TWA 0.1 mg/m ^{3}	TWA 0.1 mg/m ^{3}	Huid*	TWA 0.1 mg/m ^{3}	TWA 0.1 mg/m ^{3}

	STEL 0.3 mg/m 3<br>Pelle*	STEL 0.3 mg/m 3<br>Ceiling 0.29 mg/m 3<br>Ceiling 0.11 ppm<br>C(A4)<br>P*	STEL 0.3 mg/m 3<br>TWA 0.1 mg/m 3	STEL 0.3 mg/m 3<br>iho*	H*
**Chemical name**	**Austria**	**Switzerland**	**Poland**	**Norway**	**Ireland**
glycerol		SS-C**<br>TWA 50 mg/m 3<br>STEL 100 mg/m 3	TWA 10 mg/m 3		TWA 10 mg/m 3<br>STEL 30 mg/m 3
sodium azide	H*<br>STEL 0.3 mg/m 3<br>TWA 0.1 mg/m 3	TWA 0.2 mg/m 3<br>STEL 0.4 mg/m 3	TWA 0.1 mg/m 3<br>STEL 0.3 mg/m 3	TWA 0.1 mg/m 3<br>STEL 0.1 mg/m 3	TWA 0.1 mg/m 3<br>STEL 0.3 mg/m 3<br>Skin

8.2. Exposure controls

Appropriate engineering controls
Showers, eyewash stations, and ventilation systems.

Individual protection measures, such as personal protective equipment

**Eye/face protection**	Safety glasses with side-shields
**Skin protection**	Wear protective gloves and protective clothing
**Hand protection**	Impervious gloves.
**Other**	Wear suitable protective clothing.
**Respiratory protection**	When workers are facing concentrations above the exposure limit they must use appropriate certified respirators.

Environmental Exposure Controls
No information available.

SECTION 9: Physical and chemical properties

9.1. Information on basic physical and chemical properties

**Physical state**	Liquid
**Color**	Colorless
**Odor**	No information available

**Property**	**Values**	**Remarks • Method**
**pH**	7.5	@ 20 °C
**Melting point/freezing point**	No information available	No information available
**Boiling point or initial boiling point and boiling range**	No information available	No information available
**Flash point**	No information available	No information available.
**Evaporation rate**	No information available	No information available
**Flammability**	No information available	No information available
**Upper/lower flammability or explosive limits**	No information available	No information available
**Vapor pressure**	No information available	No information available
**Relative vapor density**	No information available	No information available
**Density and/or relative density**	No information available	No information available
**Solubility**	No information available.	No information available
**Partition coefficient: n-octanol/water**	No information available	No information available
**Autoignition temperature**	No information available	No information available
**Decomposition temperature**	No information available	No information available.
**Viscosity**	No information available	No information available
**Explosive properties**	No information available	No information available
**Oxidizing properties**	No information available	No information available

9.2. Other information

**Softening point**	No information available

**Molecular Weight**	No information available
**Solubility in other solvents**	No information available
**VOC content**	No information available
**Liquid Density**	No information available

SECTION 10: Stability and reactivity

10.1. Reactivity

No information available.

10.2. Chemical stability

Stable under normal conditions.

10.3. Possibility of hazardous reactions

**Hazardous polymerization**	Hazardous polymerization does not occur.
**Hazardous reactions**	None under normal processing.

10.4. Conditions to avoid

Extremes of temperature and direct sunlight. Over a period of time, sodium azide may react with copper, lead, brass, or solder in plumbing systems to form an accumulation of the HIGHLY EXPLOSIVE compounds of lead azide & copper azide.

10.5. Incompatible materials

Strong oxidizing agents, Strong acids.

10.6. Hazardous decomposition products

Nitrogen oxides (NOx).

SECTION 11: Toxicological information

11.1. Information on hazard classes as defined in Regulation (EC) No 1272/2008

This product is for experimental uses only. The product has not been completely analyzed and all of the hazards may not be known. Please use caution while handling this product.

Chemical name	LD50 Oral	LD50 Dermal	LC50 Inhalation
glycerol	= 12600 mg/kg (Rat)	> 10 g/kg (Rabbit)	> 570 mg/m^3 (Rat) 1 h
sodium azide	= 27 mg/kg (Rat)	= 20 mg/kg (Rabbit) = 50 mg/kg (Rat)	-

Information on likely routes of exposure

**Inhalation**	Avoid breathing vapors or mists.
**Eye contact**	Avoid contact with eyes.
**Skin contact**	Avoid contact with skin.
**Ingestion**	Ingestion may cause gastrointestinal irritation, nausea, vomiting and diarrhea.

**Symptoms**	Symptoms of allergic reaction may include rash, itching, swelling, trouble breathing, tingling of the hands and feet, dizziness, lightheadedness, chest pain, muscle pain, or flushing.
**Skin corrosion/irritation**	No information available.
**Serious eye damage/eye irritation**	No information available.
**Sensitization**	No information available.
**Mutagenic effects**	No information available.

Carcinogenic effects No information available.
Reproductive toxicity No information available.
STOT - single exposure No information available.
STOT - repeated exposure No information available.
Aspiration Hazard No information available.

11.2. Information on other hazards

No information available.

SECTION 12: Ecological information

12.1. Toxicity

Chemical name	Toxicity to algae	Toxicity to fish	Toxicity to daphnia and other aquatic invertebrates
glycerol	-	LC50 51 - 57 mL/L (Oncorhynchus mykiss) 96 h	EC50 500 mg/L (Daphnia magna) 24 h
sodium azide	EC50 0.35 mg/L (Pseudokirchneriella subcapitata) 96 h	LC50 0.8 mg/L (Oncorhynchus mykiss) 96 h LC50 5.46 mg/L (Pimephales promelas) 96 h LC50 0.7 mg/L (Lepomis macrochirus) 96 h	LC100 1 mg/L (Orconectes rusticus) 96 h

12.2. Persistence and degradability

No information available

12.3. Bioaccumulative potential

Bioaccumulation

Chemical name	Octanol-Water Partition Coefficient
glycerol	-1.76

Bioconcentration factor (BCF) No information available.

12.4. Mobility in soil

No information available.

12.5. Results of PBT and vPvB assessment

No information available.

12.6. Endocrine disrupting properties

This product does not contain any known or suspected endocrine disruptors

12.7. Other adverse effects

No information available

SECTION 13: Disposal considerations

13.1. Waste treatment methods

Waste from residues / unused Dispose of in accordance with local regulations.

**products**	
**Contaminated packaging**	Empty containers should be taken to an approved waste handling site for recycling or disposal.
**Other information**	Waste codes should be assigned by the user based on the application for which the product was used.

SECTION 14: Transport information

IMDG/IMO

**14.1 UN number**	Not regulated
**14.2 UN proper shipping name**	Not regulated
**14.3 Transport hazard class(es)**	Not regulated
**14.4 Packing group**	Not regulated
**14.5 Environmental hazards**	None
**14.6 Special precautions for user**	None
**14.7 Maritime transport in bulk according to IMO instruments**	Not regulated

ADR/RID

**14.1 UN number**	Not regulated
**14.2 UN proper shipping name**	Not regulated
**14.3 Transport hazard class(es)**	Not regulated
**14.4 Packing group**	Not regulated
**14.5 Environmental hazards**	None
**14.6 Special precautions for user**	None

IATA

**14.1 UN number**	Not regulated
**14.2 UN proper shipping name**	Not regulated
**14.3 Transport hazard class(es)**	Not regulated
**14.4 Packing group**	Not regulated
**14.5 Environmental hazards**	None
**14.6 Special precautions for user**	None

SECTION 15: Regulatory information

15.1. Safety, health and environmental regulations/legislation specific for the substance or mixture

Candidate List of Substances of Very High Concern for Authorization Information

This product does not contain Substances of Very High Concern (SVHC).

SEVESO Directive Information

This product does not contain substances identified in the SEVESO Directive.

International inventories

**TSCA 8(b)**	TSCA 8(b)
**DSL/NDSL**	DSL/NDSL
**EINECS/ELINCS**	EINECS/ELINCS
**ENCS**	ENCS
**IECSC**	IECSC
**KECL**	KECL
**PICCS**	PICCS
**AICS**	AICS

International inventories legend

TSCA - United States Toxic Substances Control Act Section 8(b) Inventory
DSL/NDSL - Canadian Domestic Substances List/Non-Domestic Substances List
EINECS/ELINCS - European Inventory of Existing Commercial Chemical Substances/EU List of Notified Chemical Substances

ENCS - Japan Existing and New Chemical Substances
IECSC - China Inventory of Existing Chemical Substances
KECL - Korean Existing and Evaluated Chemical Substances
PICCS - Philippines Inventory of Chemicals and Chemical Substances
AICS - Australian Inventory of Chemical Substances

15.2. Chemical safety assessment

For this substance a chemical safety assessment has not been carried out

SECTION 16: Other information

Full text of H-Statements referred to under Sections 2 and 3

H300 - Fatal if swallowed
H400 - Very toxic to aquatic life
H410 - Very toxic to aquatic life with long lasting effects
EUH032 - Contact with acids liberates very toxic gas

Classification procedure: Expert judgment and weight of evidence determination.
Issuing Date: 2017-07-10
Revision Date: 2022-12-14

Disclaimer

The information provided in this Safety Data Sheet is correct to the best of our knowledge, information and belief at the date of its publication. The information given is designed only as a guidance for safe handling, use, processing, storage, transportation, disposal and release and is not to be considered a warranty or quality specification. The information relates only to the specific material designated and may not be valid for such material used in combination with any other materials or in any process, unless specified in the text.

Safety Data Sheet (SDS) According to the REACH Regulation (EC) No. 1907/2006

Issuing Date: 2017-11-06 **Revision Date:** 2023-02-15 **Version:** 2

SECTION 1: Identification of the substance/mixture and of the company/undertaking

1.1. Product identifier

Product No 7074

Product name Anti-rabbit IgG, HRP-linked Antibody

Contains

Chemical name	Index No.	CAS No
glycerol (30-60)	Not Listed	56-81-5

1.2. Relevant identified uses of the substance or mixture and uses advised against

Identified uses For research use only

1.3. Details of the supplier of the safety data sheet

Importer
Cell Signaling Technology Europe B.V.
Dellaertweg 9b
2316 WZ Leiden
The Netherlands
TEL: +31 (0)71 7200 200
FAX: +31 (0)71 891 0019

Manufacturer
Cell Signaling Technology, Inc.
3 Trask Lane
Danvers, MA 01923
United States
TEL: +1 978 867 2300
FAX: +1 978 867 2400

Website www.cellsignal.com
E-mail Address info@cellsignal.eu

1.4. Emergency telephone number

CHEMTREC 24 hours a day, 7 days a week, 365 days a year
+1 703 527 3887 (INTERNATIONAL) +1 800 424 9300 (NORTH AMERICA)

Europe 112

SECTION 2: Hazards identification

2.1. Classification of the substance or mixture

Regulation (EC) No. 1272/2008

This substance is classified as not hazardous according to regulation (EC) 1272/2008 [CLP]

2.2. Label elements

Signal word

None.

Hazard statement(s)
None.

Precautionary statement(s)
None.

2.3. Other hazards

May produce an allergic reaction.

For the full text of the H-phrases & EUH-phrases mentioned in this Section, see Section 16

SECTION 3: Composition/information on ingredients

Chemical name	CAS No	Weight-%	EC No	Classification (1272/2008)	REACH Registration Number
glycerol	56-81-5	30-60	200-289-5	-	no data available

For the full text of the H-phrases & EUH-phrases mentioned in this Section, see Section 16

SECTION 4: First aid measures

4.1. Description of first aid measures

General advice Use first aid treatment according to the nature of the injury. When symptoms persist or in all cases of doubt seek medical advice.
Inhalation Move to fresh air.
Skin contact Wash skin with soap and water.
Eye contact Rinse thoroughly with plenty of water, also under the eyelids.
Ingestion Clean mouth with water and afterwards drink plenty of water.

4.2. Most important symptoms and effects, both acute and delayed

No information available.

4.3. Indication of any immediate medical attention and special treatment needed

Notes to physician Treat symptomatically.

SECTION 5: Firefighting measures

5.1. Extinguishing media

Suitable Extinguishing Media Use extinguishing measures that are appropriate to local circumstances and the surrounding environment.
Unsuitable Extinguishing Media No information available.

5.2. Special hazards arising from the substance or mixture

Thermal decomposition can lead to release of irritating gases and vapors.

5.3. Advice for firefighters

Wear self-contained breathing apparatus and protective suit. Use personal protective equipment.

SECTION 6: Accidental release measures

6.1. Personal precautions, protective equipment and emergency procedures

For non-emergency personnel Avoid contact with skin, eyes and clothing. Use personal protective equipment. For personal protection see section 8.
For emergency responders Use personal protection recommended in Section 8.

6.2. Environmental precautions

Prevent further leakage or spillage if safe to do so. Prevent product from entering drains. Prevent entry into waterways, sewers, basements or confined areas.

6.3. Methods and material for containment and cleaning up

Methods for containment Prevent further leakage or spillage if safe to do so.
Methods for cleaning up Soak up with inert absorbent material. Pick up and transfer to properly labeled containers.

6.4. Reference to other sections

See Sections 8 & 13 for additional information.

SECTION 7: Handling and storage

7.1. Precautions for safe handling

Handle in accordance with good industrial hygiene and safety practice. Wear personal protective equipment. Avoid contact with skin, eyes and clothing. Remove and wash contaminated clothing before re-use.

7.2. Conditions for safe storage, including any incompatibilities

Keep container tightly closed in a dry and well-ventilated place.

7.3. Specific end use(s)

Use as a laboratory reagent.

SECTION 8: Exposure controls/personal protection

8.1. Control parameters

Chemical name	European Union	United Kingdom	France	Spain	Germany
glycerol		STEL 30 mg/m³<br>TWA 10 mg/m³	TWA 10 mg/m³	TWA 10 mg/m³	Ceiling / Peak: 400 mg/m³<br>TWA: 200 mg/m³
**Chemical name**	**Italy**	**Portugal**	**Netherlands**	**Finland**	**Denmark**
glycerol		TWA 10 mg/m³		TWA 20 mg/m³	
**Chemical name**	**Austria**	**Switzerland**	**Poland**	**Norway**	**Ireland**
glycerol		SS-C**<br>TWA 50 mg/m³<br>STEL 100 mg/m³	TWA 10 mg/m³		TWA 10 mg/m³<br>STEL 30 mg/m³

8.2. Exposure controls

Appropriate engineering controls
Showers, eyewash stations, and ventilation systems.

Individual protection measures, such as personal protective equipment

**Eye/face protection**	Safety glasses with side-shields
**Skin protection**	Wear protective gloves and protective clothing
**Hand protection**	Impervious gloves.
**Other**	Wear suitable protective clothing.
**Respiratory protection**	When workers are facing concentrations above the exposure limit they must use appropriate certified respirators.

Environmental Exposure Controls
No information available.

SECTION 9: Physical and chemical properties

9.1. Information on basic physical and chemical properties

**Physical state**	Liquid
**Color**	Colorless
**Odor**	No information available

Property	Values	Remarks • Method
**pH**	7.5	@ 20 °C
**Melting point/freezing point**	No information available	No information available
**Boiling point or initial boiling point and boiling range**	No information available	No information available
**Flash point**	No information available	No information available.
**Evaporation rate**	No information available	No information available
**Flammability**	No information available	No information available
**Upper/lower flammability or explosive limits**	No information available	No information available
**Vapor pressure**	No information available	No information available
**Relative vapor density**	No information available	No information available
**Density and/or relative density**	No information available	No information available
**Solubility**	No information available.	No information available
**Partition coefficient: n-octanol/water**	No information available	No information available
**Autoignition temperature**	No information available	No information available
**Decomposition temperature**	No information available	No information available.
**Viscosity**	No information available	No information available
**Explosive properties**	No information available	No information available
**Oxidizing properties**	No information available	No information available

9.2. Other information

**Softening point**	No information available
**Molecular Weight**	No information available
**Solubility in other solvents**	No information available
**VOC content**	No information available
**Liquid Density**	No information available

SECTION 10: Stability and reactivity

10.1. Reactivity

No information available.

10.2. Chemical stability

Stable under normal conditions.

10.3. Possibility of hazardous reactions

**Hazardous polymerization**	Hazardous polymerization does not occur.
**Hazardous reactions**	None under normal processing.

10.4. Conditions to avoid

Extremes of temperature and direct sunlight.

10.5. Incompatible materials

No information available.

10.6. Hazardous decomposition products

None under normal use conditions.

SECTION 11: Toxicological information

11.1. Information on hazard classes as defined in Regulation (EC) No 1272/2008

This product is for experimental uses only. The product has not been completely analyzed and all of the hazards may not be known. Please use caution while handling this product.

Chemical name	LD50 Oral	LD50 Dermal	LC50 Inhalation
glycerol	= 12600 mg/kg (Rat)	> 10 g/kg (Rabbit)	> 570 mg/m^3 (Rat) 1 h

Information on likely routes of exposure

**Inhalation**	Avoid breathing vapors or mists.
**Eye contact**	Avoid contact with eyes.
**Skin contact**	Avoid contact with skin.
**Ingestion**	Ingestion may cause gastrointestinal irritation, nausea, vomiting and diarrhea.

**Symptoms**	No information available.
**Skin corrosion/irritation**	No information available.
**Serious eye damage/eye irritation**	No information available.
**Sensitization**	No information available.
**Mutagenic effects**	No information available.
**Carcinogenic effects**	No information available.
**Reproductive toxicity**	No information available.
**STOT - single exposure**	No information available.
**STOT - repeated exposure**	No information available.
**Aspiration Hazard**	No information available.

11.2. Information on other hazards

No information available.

SECTION 12: Ecological information

12.1. Toxicity

Chemical name	Toxicity to algae	Toxicity to fish	Toxicity to daphnia and other aquatic invertebrates
glycerol	-	LC50 51 - 57 mL/L (Oncorhynchus mykiss) 96 h	EC50 500 mg/L (Daphnia magna) 24 h

12.2. Persistence and degradability

No information available

12.3. Bioaccumulative potential

Bioaccumulation

Chemical name	Octanol-Water Partition Coefficient
glycerol	-1.76

Bioconcentration factor (BCF) No information available.

12.4. Mobility in soil

No information available.

12.5. Results of PBT and vPvB assessment

No information available.

12.6. Endocrine disrupting properties

This product does not contain any known or suspected endocrine disruptors

12.7. Other adverse effects

No information available

SECTION 13: Disposal considerations

13.1. Waste treatment methods

**Waste from residues / unused products**	Dispose of in accordance with local regulations.
**Contaminated packaging**	Empty containers should be taken to an approved waste handling site for recycling or disposal.
**Other information**	Waste codes should be assigned by the user based on the application for which the product was used.

SECTION 14: Transport information

IMDG/IMO

**14.1 UN number**	Not regulated
**14.2 UN proper shipping name**	Not regulated
**14.3 Transport hazard class(es)**	Not regulated
**14.4 Packing group**	Not regulated
**14.5 Environmental hazards**	None

**14.6 Special precautions for user**	None
**14.7 Maritime transport in bulk according to IMO instruments**	Not regulated

ADR/RID

**14.1 UN number**	Not regulated
**14.2 UN proper shipping name**	Not regulated
**14.3 Transport hazard class(es)**	Not regulated
**14.4 Packing group**	Not regulated
**14.5 Environmental hazards**	None
**14.6 Special precautions for user**	None

IATA

**14.1 UN number**	Not regulated
**14.2 UN proper shipping name**	Not regulated
**14.3 Transport hazard class(es)**	Not regulated
**14.4 Packing group**	Not regulated
**14.5 Environmental hazards**	None
**14.6 Special precautions for user**	None

SECTION 15: Regulatory information

15.1. Safety, health and environmental regulations/legislation specific for the substance or mixture

Candidate List of Substances of Very High Concern for Authorization Information

This product does not contain Substances of Very High Concern (SVHC).

SEVESO Directive Information

This product does not contain substances identified in the SEVESO Directive.

International inventories

**TSCA 8(b)**	-
**DSL/NDSL**	Complies
**EINECS/ELINCS**	-
**ENCS**	-
**IECSC**	Complies
**KECL**	-
**PICCS**	-
**AICS**	Complies

International inventories legend

TSCA - United States Toxic Substances Control Act Section 8(b) Inventory
DSL/NDSL - Canadian Domestic Substances List/Non-Domestic Substances List
EINECS/ELINCS - European Inventory of Existing Commercial Chemical Substances/EU List of Notified Chemical Substances
ENCS - Japan Existing and New Chemical Substances
IECSC - China Inventory of Existing Chemical Substances
KECL - Korean Existing and Evaluated Chemical Substances
PICCS - Philippines Inventory of Chemicals and Chemical Substances
AICS - Australian Inventory of Chemical Substances

15.2. Chemical safety assessment

For this substance a chemical safety assessment has not been carried out

SECTION 16: Other information

Full text of H-Statements referred to under Sections 2 and 3

This substance/mixture does not meet the criteria for classification in accordance with Regulation (EC) No. 1272/2008

Classification procedure: Expert judgment and weight of evidence determination.
Issuing Date: 2017-11-06
Revision Date: 2023-02-15

Disclaimer

The information provided in this Safety Data Sheet is correct to the best of our knowledge, information and belief at the date of its publication. The information given is designed only as a guidance for safe handling, use, processing, storage, transportation, disposal and release and is not to be considered a warranty or quality specification. The information relates only to the specific material designated and may not be valid for such material used in combination with any other materials or in any process, unless specified in the text.

Version: 5   Revision Date: 03/12/2021

Purified anti-β-Amyloid, 1-16 Antibody (Previously Covance catalog# SIG-39320)

**Catalog# / Size**	803004 / 25 µL<br>803001 / 200 µL<br>803002 / 500 µL<br>803003 / 1 mL
**Clone**	6E10
**Regulatory Status**	RUO
**Other Names**	AAA, ABETA, ABPP, AD1, APPI, CTFgamma, CVAP, PN-II, PN2, Amyloid beta A4 protein, preA4, protease, peptidase nexin-II, beta-amyloid peptide, alzheimer disease amyloid protein, cerebral vascular amyloid peptide, APP, Amyloid Precursor Protein
**Previously**	Signet Catalog# 9320-02<br>Signet Catalog# 9320-05<br>Signet Catalog# 9320-10<br>Covance Catalog# SIG-39320
**Isotype**	Mouse IgG1, κ
**Description**	Alzheimer's disease is characterized by the accumulation of aggregated Aβ peptides in senile plaques and vascular deposits. Aβ peptides are derived from amyloid precursor proteins (APP) through sequential proteolytic cleavage of APP by β-secretases and γ-secretases generating diverse Aβ species. Aβ can aggregate to form soluble oligomeric species and insoluble fibrillar or amorphous assemblies. Some forms of the aggregated peptides are toxic to neurons.

Product Details

**Verified Reactivity**	Human
**Antibody Type**	Monoclonal
**Host Species**	Mouse
**Formulation**	Phosphate-buffered solution (no preservatives or carrier proteins).
**Preparation**	The antibody was purified by affinity chromatography.
**Concentration**	1 mg/mL
**Storage & Handling**	The antibody solution should be stored undiluted between 2°C and 8°C. Please note the storage condition for this antibody has been changed from -20°C to between 2°C and 8°C. You can also check your vial or your CoA to find the most accurate storage condition for this antibody.
**Application**	WB - *Quality tested*<br>Direct ELISA, IHC-P - *Verified*<br>IHC-F, EM, ICC - *Reported in the literature, not verified in house*
**Recommended Usage**	Each lot of this antibody is quality control tested by western blotting. For western blotting, the suggested use of this reagent is 1 µg/mL. For Direct ELISA, the suggested use of this reagent is 0.002 - 0.02 µg/mL. For immunohistochemistry on formalin-fixed paraffin-embedded tissue sections, the suggested use of this reagent is 0.2 - 5.0 µg/mL. It is recommended that the reagent be titrated for optimal performance for each application.
**Application Notes**	This antibody is reactive to amino acid residue 1-16 of beta amyloid. The epitope lies within amino acids 3-8 of beta amyloid (EFRHDS).<br><br>This antibody clone has been reported for use in immunohistochemistry of free-floating sections[2,13].
**Additional Product Notes**	View more applications data for this product in our Scientific Poster Library.
**Application References**	

1. Thakker DR, *et al*. 2009. *Proc. Natl. Acad. Sci. USA.* 106(11):4501-6. **(IHC)** PubMed

(PubMed link indicates BioLegend citation)

2. Oddo S, *et al.* 2005. *Proc. Natl. Acad. Sci. USA.* 102(8):3046-51. **(IHC-other)** PubMed
3. Herzig M, *et al.* 2004. *Nat. Neuro.* 7(9):954-959. **(WB)** PubMed
4. Zheng Y, *et al.* 2012. *PLoS One* 6:39035. **(IHC-F)** PubMed
5. Abramowksi D, *et al. J Neurosci.* 32:1273. **(WB)** PubMed
6. Forny-Germano L, *et al.* 2014. *J. Neurosci.* 34:13629. **(WB, IHC)** PubMed
7. Gowert NS, *et al.* 2014. *PLoS One* 2:e90523. **(ICC, EM)** PubMed
8. Sandoval-Hernández A, *et al.* 2015. *PLoS One*. 10: 0145467. **(IHC-F)**
9. Kumar R, *et al.* 2016. *Brain.* 139:174-92 **(WB)**
10. Miyamoto T, *et al.* 2016. J. Biol. Chem. 291:1719-34. **(WB)**
11. Saito S, *et al.* 2017. *Acta Neuropathol. Commun.* 5:26-9. **(IHC-P)** PubMed
12. Omata Y, *et al.* 2016. *Aging (Albany NY)* 8(3):427. **(IHC-P)** PubMed
13. Peng W, *et al.* 2016. *Neurobiol. Dis.* 93:215. **(IHC-other)** PubMed
14. Mandler M, *et al.* 2015. *PLoS One.* e0115237. **(WB, IHC, ELISA)** PubMed

Product Citations

1. Abud EM *et al.* 2017. Neuron. 94(2):278-293 . PubMed
2. Wang X, *et al.* 2019. Cell Res. 29:787. PubMed
3. Eede P, *et al.* 2020. EMBO Rep. 21:e48530. PubMed
4. Sogorb-Esteve A, *et al.* 2018. Mol Neurobiol. 55:5047. PubMed
5. Turnbull MT, *et al.* 2018. Front Mol Neurosci. 11:51. PubMed
6. Lam AB, *et al.* 2021. Cell Rep. 36:109753. PubMed
7. Joly S, *et al.* 2021. Int J Mol Sci. 22:. PubMed
8. Rupawala H, *et al.* 2022. Brain Commun. 4:fcac192. PubMed
9. Lee NK, *et al.* 2020. Int J Mol Sci. :21. PubMed
10. Hsu CN, *et al.* 2021. PLOS Computational Biology. 17(5):e1008967. PubMed
11. Zhang H, *et al.* 2020. J Neurosci. 40:5347. PubMed
12. Shi Y, *et al.* 2020. J Alzheimers Dis. 76:1375. PubMed

RRID

AB_2715854 (BioLegend Cat. No. 803004)
AB_2564653 (BioLegend Cat. No. 803001)
AB_2564654 (BioLegend Cat. No. 803002)
AB_2564652 (BioLegend Cat. No. 803003)

Antigen Details

Structure

Amyloid precursor protein is a 770 amino acid protein with a molecular mass of ~100 kD. According to the UniProtKB database, APP (ID# P05067) has 11 isoforms (34 to ~90 kD) and the 770 form has been designated as the canonical form. Isoform APP695 is the predominant form expressed in neuronal tissue. Isoforms APP751 and APP770 are widely expressed in non-neuronal cells. Isoform APP751 is the most abundant form in T-lymphocytes. Aβ denotes peptides of 36-43 amino acids generated from cleavage of APP by secretases. Aβ has an apparent molecular mass of about 4 kD.

Distribution

Tissue distribution: Primarily nervous system, but also adipose tissue, intestine, muscle. Cellular distribution: Cytosol, endosomes, nucleus, plasma membrane, extracellular, and golgi apparatus.

Function

The normal function of Aβ is not well understood. Several potential physiological roles have been proposed, including: activation of kinase enzymes; protection against oxidative stress; regulation of cholesterol transport; transcription factor, and as an anti-microbial agent.

Biology Area

Cell Biology, Neurodegeneration, Neuroscience, Protein Misfolding and Aggregation

Molecular Family

APP/β-Amyloid

Antigen References

1. Kumar A, *et al.* 2015. Pharmacol. Rep. 67(2):195.
2. Sadigh-Eteghad S, *et al.* 2015. Med. Princ. Pract. 24(1):1
3. Hampel H, *et al.* 2015. *Expert Rev. Neurother.* 15(1):83.
4. Puig KL, *et al.* 2012. *Exp. Gerontol.* 48(7): 608.
5. Selkoe DJ, *et al.* 2016. *EMBO Mol. Med.* 8(6):595.
6. Walsh DM, *et al.* 2007. *J. Neurochem.* 101(5):1172.

Gene ID

351

Related Protocols

- Western Blotting Protocol

- Sandwich ELISA Protocol
- Immunohistochemistry Protocol for Paraffin-Embedded Sections

Other Formats

Alexa Fluor® 488 anti-β-Amyloid, 1-16, Anti-β-Amyloid, 1-16, Biotin anti-β-Amyloid, 1-16 , Purified anti-β-Amyloid, 1-16 , HRP anti-β-Amyloid, 1-16, Alexa Fluor® 594 anti-β-Amyloid, 1-16, Alexa Fluor® 647 anti-β-Amyloid, 1-16, Ultra-LEAF™ Purified anti-β-Amyloid, 1-16, Spark YG™ 570 anti-β-Amyloid, 1-16

Product Data

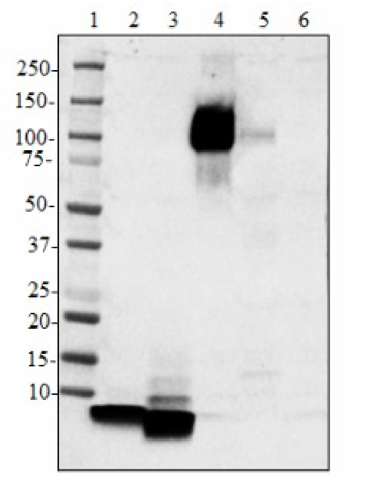

Western blot of purified anti-β-amyloid, 1-16 antibody (clone 6E10). Lane 1: Molecular weight marker; Lane 2: 50 ng of the human Aβ1-40 peptide; Lane 3: 50 ng of the Aβ1-42 peptide; Lane 4: 50 ng of the recombinant human APP751 protein; Lane 5: 20µg of the human brain lysate; Lane 6: 50 ng of the rodent Aβ1-42 peptide. The blot was incubated with 1 µg/mL of the primary antibody overnight at 4°C, followed by incubation with the HRP goat anti-mouse IgG antibody (Cat. No. 405306). Enhanced chemiluminescence was used as the detection system.

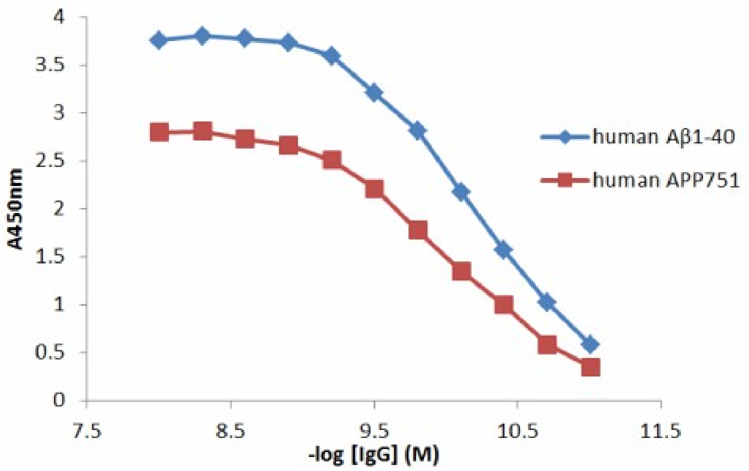

Direct ELISA of purified anti-β-amyloid 1-16 (clone 6E10) antibody binding to the plate-immobilized human Aβ1-40 and the recombinant human APP751. ELISA was performed by coating the wells with 100 ng of peptide or recombinant protein. The wells were then incubated with the primary antibody at 37°C for 45 minutes, followed by incubation with HRP labeled goat anti-mouse IgG secondary antibody. TMB (3, 3', 5, 5' tetramethylbenzidine, Cat. No. 421501) was used as the detection system.

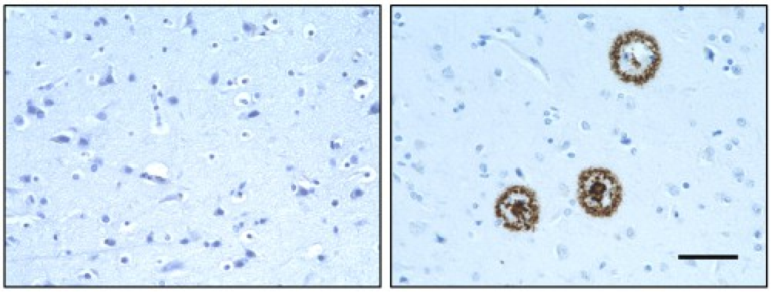

IHC staining of purified anti-β-Amyloid, 1-16 antibody (clone 6E10) on the formalin-fixed paraffin-embedded normal human (left panel) and Alzheimer's disease (right panel) brain tissues. Following antigen retrieval using 70% formic acid for 20 minutes, the tissues were incubated with 1µg/mL of the primary antibody for 1 hour at room temperature. BioLegend's Ultra-Streptavidin (USA) HRP Detection Kit (Multi-Species, DAB, Cat. No. 929901) was used for detection followed by hematoxylin counterstaining, according to the protocol provided. The images were captured with a 40X objective. Scale bar: 50 µm

For research use only. Not for diagnostic use. Not for resale. BioLegend will not be held responsible for patent infringement or other violations that may occur with the use of our products.

*These products may be covered by one or more Limited Use Label Licenses (see the BioLegend Catalog or our website, www.biolegend.com/ordering#license). BioLegend products may not be transferred to third parties, resold, modified for resale, or used to manufacture commercial products, reverse engineer functionally similar materials, or to provide a service to third parties without written approval of BioLegend. By use of these products you accept the terms and conditions of all applicable Limited Use Label Licenses. Unless otherwise indicated, these products are for research use only and are not intended for human or animal diagnostic, therapeutic or commercial use.

8999 BioLegend Way, San Diego, CA 92121 www.biolegend.com
Toll-Free Phone: 1-877-Bio-Legend (246-5343) Phone: (858) 768-5800 Fax: (877) 455-9587

Goat anti-Mouse IgG (H+L) Secondary Antibody, Biotin

Product Details	
Size	2 mL
Species Reactivity	Mouse
Host/Isotype	Goat / IgG
Class	Polyclonal
Type	Secondary Antibody
Conjugate	Biotin
Form	Lyophilized
Concentration	1.3 mg/mL
Purification	Antigen affinity chromatography
Storage buffer	PBS, pH 7.6, with 15mg/mL BSA
Contains	0.05% sodium azide
Storage conditions	4° C
RRID	AB_228305

Applications	Tested Dilution	Publications
Western Blot (WB)	1:5,000-1:20,000	-
Immunohistochemistry (IHC)	1:500-1:5,000	2 Publications
Immunocytochemistry (ICC/IF)	1:500-1:5,000	-
Flow Cytometry (Flow)	1:200-1:1,000	-
ELISA (ELISA)	1:20,000-1:400,000	-
Immunoprecipitation (IP)	1:500-1:5,000	-
Miscellaneous PubMed (Misc)	-	3 Publications

Product Specific Information

Concentration may vary slightly from lot-to-lot, see lot-specific datasheet for exact concentration.

Product # 31800 has been successfully used in Western blot, IF, ICC, IHC, IP and FACS applications.

Product # 31800 reacts with the heavy chains of mouse IgG and with the light chains common to most mouse immunoglobulins, but does not react against non-immunoglobulin serum proteins. However, this antibody may cross-react with immunoglobulins from other species.

Store product at 4°C until opened. To extend the shelf-life of this product, add an equal volume of glycerol to make a final concentration of approximately 50% glycerol and store at -20°C.

Reconstitute with 2.0 mL of distilled water.

Country of Origin: USA

Product Images For Goat anti-Mouse IgG (H+L) Secondary Antibody, Biotin

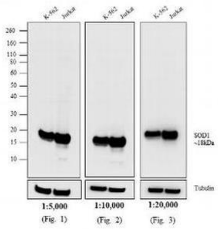

Mouse IgG (H+L) Secondary Antibody (31800) in WB
Western blot analysis was performed on whole cell extracts (30 µg lysate) of K-562 (Lane 1) and Jurkat (Lane 2). The blots were probed with Anti-SOD1 Mouse Monoclonal Antibody (Product # MA1-105, 0.5µg/mL) and detected by chemiluminescence using Goat anti-Mouse IgG (H+L) Secondary Antibody, Biotin (Product # 31800) at dilutions 1:5,000 (Fig. 1), 1:10,000 (Fig. 2) and 1:20,000 (Fig. 3). A 18 kDa band corresponding to SOD1 was observed. Known quantity of protein samples were electrophoresed using Novex® NuPAGE®12 % Bis-Tris gel (Product # NP0342BOX), XCell SureLock™ Electrophoresis System (Product # EI0002) and Novex® Sharp Pre-Stained Protein Standard (Product # LC5800). Resolved proteins were then transferred onto a nitrocellulose membrane with iBlot® 2 Dry Blotting System (Product # IB21001). The membrane was probed with the relevant primary and secondary antibody after blocking with 5 % skimmed milk. This is followed by incubating the membrane with Poly-HRP Streptavidin (Product # N200, 1:10,000). Chemiluminescent detection was performed using Pierce™ ECL Western Blotting Substrate (Product # 32106).

89 References

Th9 Cytokines Inhibit Proliferation, Promote Apoptosis, and Immune Escape in Thyroid Carcinoma Cells. Appl Biochem Biotechnol (2024)

Decoy peptides effectively inhibit the binding of SARS-CoV-2 to ACE2 on oral epithelial cells. Heliyon (2023)

Ovarian ER cistrome and transcriptome reveal chromatin interaction with LRH-1. BMC Biol (2023)

Th9 cytokines inhibit proliferation, promote apoptosis, and immune escape in thyroid carcinoma cells Research Square (2023)

RUNX1 loss renders hematopoietic and leukemic cells dependent on IL-3 and sensitive to JAK inhibition. J Clin Invest (2023)

For Research Use Only. Not for use in diagnostic procedures. Not for resale without express authorization. Products are warranted to operate or perform substantially in conformance with published Product specifications in effect at the time of sale, as set forth in the Production documentation, specifications and/or accompanying package inserts ("Documentation"). No claim of suitability for use in applications regulated by FDA is made. The warranty provided herein is valid only when used by properly trained individuals. Unless otherwise stated in the Documentation, this warranty is limited to one year from date of shipment when the Product is subjected to normal, proper and intended usage. This warranty does not extend to anyone other than the Buyer. Any model or sample furnished to Buyer is merely illustrative of the general type and quality of goods and does not represent that any Product will conform to such model or sample. NO OTHER WARRANTIES, EXPRESS OR IMPLED, ARE GRANTED INCLUDING WITHOUT LIMITATION, IMPLIED WARRANTIES OF MERCHANTABILITY, FITNESS FOR ANY PARTICULAR PURPOSE, OR NON INFRINGEMENT. BUYER'S EXCLUSIVE REMEDY FOR NON-CONFORMING PRODUCTS DURING THE WARRANTY PERIOD IS LIMITED TO REPAIR, REPLACEMENT OF OR REFUND FOR THE NON-CONFORMING PRODUCT(S) AT SELLER'S SOLE OPTION. THERE IS NO OBLIGATION TO REPAIR, REPLACE OR REFUND FOR PRODUCTS AS THE RESULT OF (I) ACCIDENT, DISASTER OR EVENT OF FORCE MAJEURE, (II) MISUSE, FAULT OR NEGLIGENCE OF OR BY BUYER, (III) USE OF THE PRODUCTS IN A MANNER FOR WHICH THEY WERE NOT DESIGNED, OR (IV) IMPROPER STORAGE AND HANDLING OF THE PRODUCTS. Unless otherwise expressly stated on the Product or in the documentation accompanying the Product, the Product is intended for research only and is not to be used for any other purpose, including without limitation, unauthorized commercial uses, in vitro diagnostic uses, ex vivo or in vivo therapeutic uses, or any type of consumption by or application to human or animals.

Cell Line Authentication Service
STR Profile Report

GENETIC TESTING BIOTECHNOLOGY

Sample Submitted By: Dr. Liegang Liu
Huazhong University of Science & Technology
Email Address: lgliu@mails.tjmu.edu.cn
Sales Order: 240122A
Cell Line Designation: Hep-G2
Date Sample Received: Jan 22th, 2024
Report Date: Jan 22th, 2024

Methodology: Twenty-one short tandem repeat (STR) loci plus the Amelogenin locus were amplified using the commercially available SiFaSTR™ 23 plex Kit. The cell line sample was processed using the ABI Prism® 3130 XL Genetic Analyzer. Data were analyzed using GeneMapper® ID v3.2 software (Applied Biosystems). Appropriate positive and negative controls were run and confirmed for each sample submitted.

Data Interpretation: Cell lines were authenticated using Short Tandem Repeat (STR) analysis as described in 2021 in ANSI Standard (ASN-0002) by the ATCC Standards Development Organization (SDO) and in Jamie L. Almeida et al., Authentication of Human and Mouse Cell Lines by Short Tandem Repeat (STR) DNA Genotype Analysis. Assay Guidance Manual. PMID: 23805434. Bookshelf ID: NBK144066.

GTB™ performs STR Profiling following ISO 9001:2008 and ISO/IEC 17025:2005 quality standards.

There are no warranties with respect to the services or results supplied, express or implied, including, without limitation, any implied warranty of merchantability or fitness for a particular purpose. Genetic Testing Biotechnology (GTB) is not liable for any damages or injuries resulting from receipt and/or improper, inappropriate, negligent or other wrongful use of the test results supplied, and/or from misidentification, misrepresentation, or lack of accuracy of those results. Your exclusive remedy against GTB and those supplying materials used in the services for any losses or damage of any kind whatsoever, whether in contract, tort, or otherwise, shall be, at GTB's option, refund of the fee paid for such service or repeat of the service.

NOTE: According to the recommendations of *IJC* on cell line authentication, the report is valid for 3 years since the issue date.

Technical Questions?
GTB Technical Support
+86-512-67486171
service@jsdna.org
Section 505, Yixin BLD
SIP, Suzhou, 215123
Jiangsu, P.R. China

Ordering Questions?
order@jsdna.org
GTB Corporation
+86-512-62806339
Section 303, Yixin BLD
SIP, Suzhou, 215123
Jiangsu, P.R. China

GENETIC TESTING BIOTECHNOLOGY

Cell Line Authentication Service
STR Profile Report

Sales Order: 240122A

	Test Results for Submitted Sample		ExPASy Reference Database Profile	
Loci	Query Profile: Hep-G2		Database Profile: Hep-G2*	
Amelogenin	X	Y		
D3S1358	15	16	15	16
D5S818	11	12	11	12
D2S1338	19	20		
TPOX	8	9	8	9
CSF1PO	10	11	10	11
Penta D	9	13		
TH01	9		9	
vWA	17		17	
D7S820	10		10	
D21S11	29	31	29	31
Penta E	15	20		
D10S1248	13			
D8S1179	15	16	15	16
D1S1656	11	12		
D18S51	13	14	13	14
D12S391	21	25		
D6S1043	13			
D19S433	15.2			
D16S539	12	13	12	13
D13S317	9	13	9	13
FGA	22	25	22	25

The allele match algorithm compares the 13 core STR loci only, even though alleles from all loci will be reported when available.

Note: Loci highlighted in grey (13 core STR loci) can be made public to verify cell identity. In order to protect the identity of the donor, ***please do not publish*** *the allele calls from all the STR loci tested.*

The sample match is based on the reference data available at the time of comparison.

Problematic cell line: Misclassified. Originally thought to be a hepatocellular carcinoma cell line but shown to be from an hepatoblastoma.

Explanation of Test Results

Cell lines with ≥80% match are derived from the same donor. Cell lines with between a 70% to 79% match require further profiling for authentication of relatedness. Cell lines with <70% match are very unlikely to be from the same donor.

- [ ] The submitted sample profile is human, but not a match for any profile in the ExPASy STR database.
- [x] The submitted profile is an exact match for the following human cell line(s) in the ExPASy STR database (13 core STR loci): Hep-G2
- [ ] The submitted profile is similar to the following ExPASy human cell line(s):

e-Signature, Technician:

e-Signature, Reviewer:

More information

Addendum: Electropherogram for the customer's sample set 1 of 1

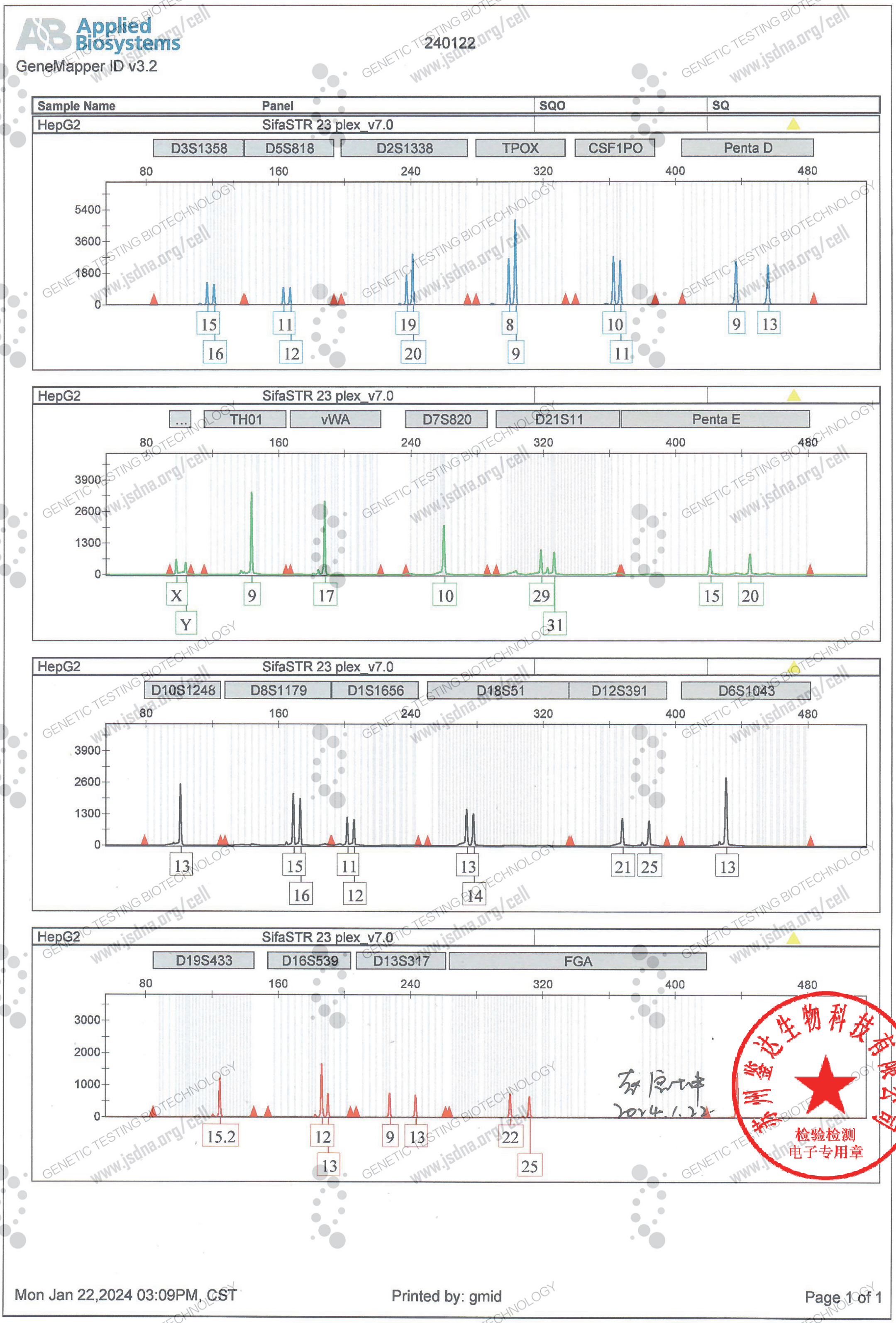

GENETIC TESTING BIOTECHNOLOGY

Cell Line Authentication Service
STR Profile Report

Export table

Accession	Name	N° Markers	Score	Amel	CSF1PO	D2S1338	D3S1358	D5S818	D7S820	D8S1179	D13S317	D16S539	D18S51	D19S433	D21S11	FGA	Penta D	Penta E	TH01	TPOX	vWA
NA	Query	NA	NA		10,11		15,16	11,12	10	15,16	9,13	12,13	13,14		29,31	22,25			9	8,9	17
CVCL_0027 *Best*	Hep-G2	13	100.00%	X,Y	10,11	19,20	15,16	11,12	10	15,16	9,13	12,13	13,14	15.2	29,31	22,25	9,13	15,20	9	8,9	17
CVCL_0027 *Worst*	Hep-G2	13	97.87%	X,Y	10,11	19,20	15,16	11,12	10	15,16,17	9,13	12,13	13,14	16	29,31	22,25	9,13	15,20	9	8,9	17
CVCL_L855	Hep-G2/2.2.15	13	100.00%	X,Y	10,11		15,16	11,12	10	15,16	9,13	12,13	13,14		29,31	22,25	9,13	15,20	9	8,9	17
CVCL_1098	Hep-G2/C3A	13	95.65%	X,Y	10,11		15,16	11,13	10	15,16	9,13	12,13	13,14		29,31	22,25	9,13	15,20	9	8,9	17
CVCL_1320 *Best*	JVM-3	13	63.64%	X,Y	10,12	21,23	16	11	10	15	13	11,12	13,16	14	29,31	24,26	12,13	14,16	9,9.3	8,9	17,19
CVCL_1320 *Worst*	JVM-3	13	62.22%	X,Y	10,12	21,23	16	11	10	15	13	11,12	13,16	14	29,31	24,26	12,13	14,16	9,9.3	8,9	17,18,19
CVCL_RM92	CBiPSC6.2	13	62.22%	X	11		15,18	11,12	10,12	9,15	9,11	11,13	15		29	22,25	2.2,11	12,14	9,9.3	8	17,18
CVCL_A8SW	TMOi001-A-4	13	62.22%	X	11		15,18	11,12	10,12	9,15	9,11	11,13	15		29	22,25	2.2,11	12,14	9,9.3	8	17,18
CVCL_A8Q0	HT29-5M21	13	61.90%	X	11,12		15	11,12	10	10	11	11,12	13		29	20,22	11	14,16	6,9	8,9	17
CVCL_0320 *Best*	HT-29	13	60.87%	X	11,12	19,23	15,17	11,12	10	10,16	11	11,12	13	14	29,30	20,22	11,13	14,16	6,9	8,9	17,19
CVCL_0320 *Worst*	HT-29	13	56.52%	X	11,12	19,23	15,17	11,12	10	10	11,12	11,12	13	14	29,30	20,22	11,13	14,16	6,9	8,9	17,19
CVCL_A8P7	HT29-5F12	13	60.87%	X	11,12		15,17	11,12	10	10,16	11,12	11,12	13		29,30	20,22	11,13	14,16	6,9	8,9	17
CVCL_A8P9	HT29-5M12	13	60.47%	X	11,12		15,17	11,12	10	10	11	11,12	13		29	20,22	11	14,16	6,9	8,9	17
CVCL_1D66 *Best*	U-CH10	13	60.47%	X	10,11	19	15,16	11	10	8,13	9,13	11,13	13	13,15	29,31	24	9,13	18	8,9	11	16
CVCL_1D66 *Worst*	U-CH10	13	59.09%	X	10,11	19	15,16	11	9,10	8,13	9,13	11,13	13	13,15	29,31	24	9,13	18	8,9	11	16
